# Chemoenzymatic total synthesis of the antibiotic (−)-13-deoxytetrodecamycin using the Diels–Alderase TedJ

**DOI:** 10.1039/d5sc05480j

**Published:** 2025-08-26

**Authors:** S. Joe Russell, Catherine R. Back, Christopher Perry, Kaiman A. Cheung, Laurence Maschio, Sacha N. Charlton, Nicholas R. Lees, Monserrat Manzo-Ruiz, Martin A. Hayes, Marc W. van der Kamp, Paul R. Race, Christine L. Willis

**Affiliations:** a School of Chemistry, University of Bristol Cantock's Close Bristol BS8 1TS UK chris.willis@bristol.ac.uk; b School of Biochemistry, University Walk, University of Bristol Bristol BS8 1TD UK; c School of Natural and Environmental Sciences, Newcastle University Newcastle Upon Tyne NE1 7RU UK paul.race1@newcastle.ac.uk; d Discovery Sciences, Biopharmaceuticals R&D, AstraZeneca Pepparedsleden 1 SE-431 83 Mölndal Sweden

## Abstract

The tetrodecamycins are tetracyclic natural products that exhibit potent antimicrobial activity against a multitude of drug-resistant pathogens. These compounds are structurally distinguished by the presence of a tetronate ring and *trans*-decalin with six contiguous asymmetric centres united by a seven-membered oxygen heterocycle. Herein we describe the first total synthesis of the antibiotic (−)-13-deoxytetrodecamycin. Our strategy is predicated on an enantioselective [4 + 2]-cycloaddition catalysed by the FAD-dependent Diels–Alderase TedJ, forming the *trans*-decalin with concomitant creation of two rings and four contiguous stereocenters with exquisite selectivity under mild conditions. In complementary studies, *in vitro* enzyme assays, X-ray crystallography and computational modelling are used to provide molecular insights into the TedJ catalysed reaction. These studies illustrate the power of adopting a chemoenzymatic approach for the enantioselective synthesis of a target compound which would be difficult to achieve using non-biological methods and provide a practical demonstration of the use of Diels–Alder biocatalysts in total synthesis. This approach has potentially widespread value in the global challenge of discovery and development of new antibiotics.

## Abstract 2

A biocatalytic Diels–Alder reaction creates *trans*-decalin I with four new stereocenters in the synthesis of the antibiotic (−)-13-deoxytetrodecamycin. X-ray crystallography and modelling provide insights into the biocatalytic [4 + 2]-cycloaddition.

## Graphical abstract

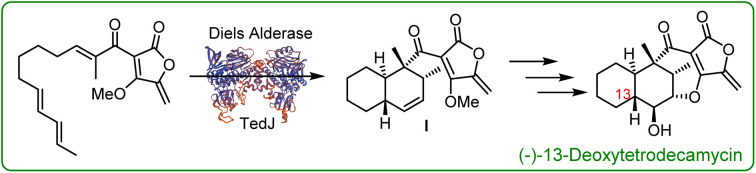

## Introduction

**Scheme 1:**
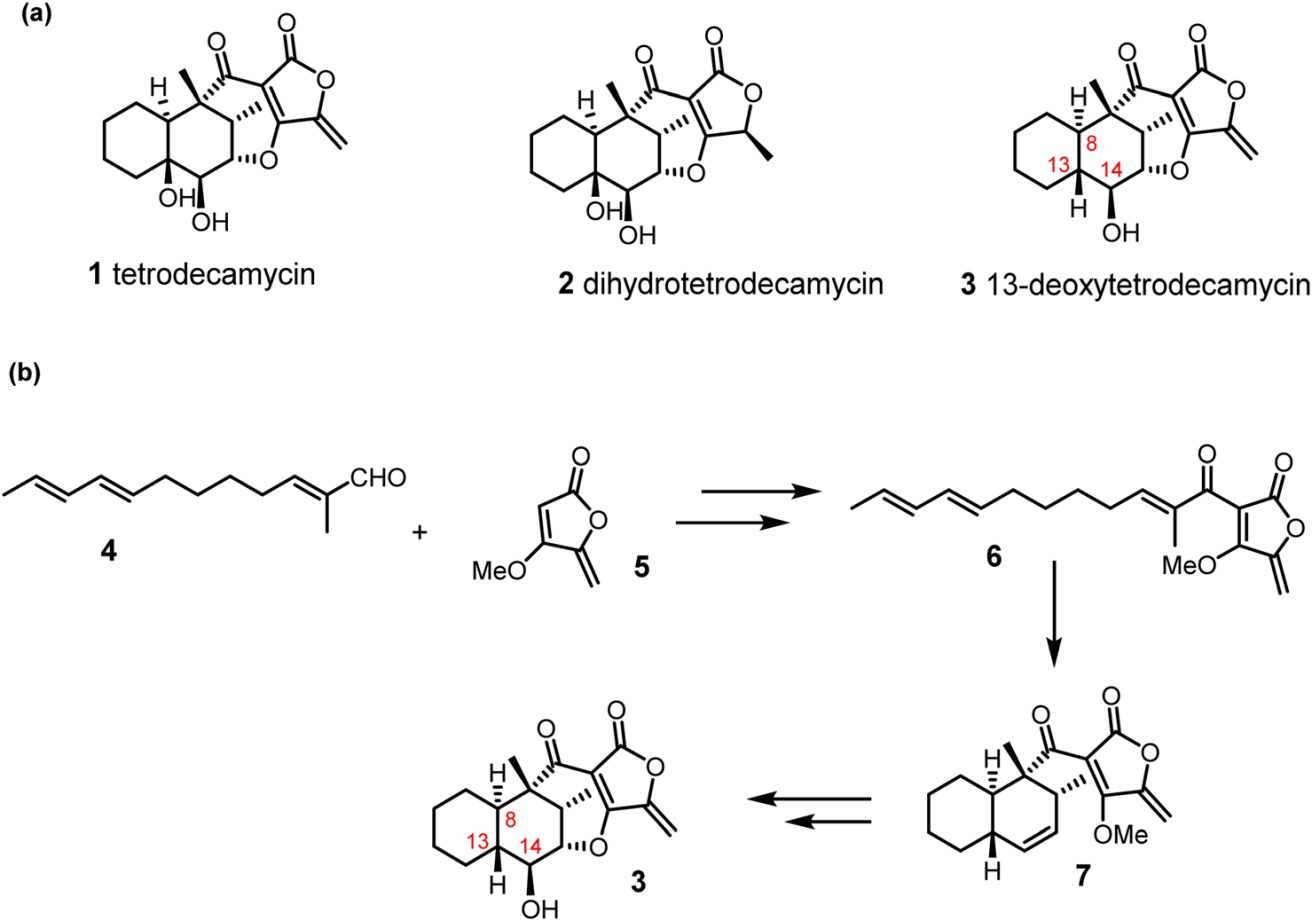
(a). Structures of three tetrodecamycins. (b). Outline of our initially proposed synthetic strategy to 13-deoxytetrodecamycin 3.

The tetrodecamycins are a family of polyketide derived natural products assembled on an unusual 6,6,7,5-tetracyclic framework encompassing a *trans*-decalin and tetronate ring united by a 7-membered oxygen heterocycle (Scheme 1). Tetrodecamycin 1 and dihydrotetrodecamycin 2 were first isolated from extracts of *Streptomyces nashvillensis* MJ885-mF8, from a soil sample collected in Japan and their structures were elucidated by spectroscopic methods and X-ray crystallography.^1^ Tetrodecamycin showed antibacterial activity against both the Gram-negative microorganism *Photobacterium damselae* subsp. *piscicida*, the causative agent of pseudotuberculosis in fish, as well as Gram-positive bacteria including methicillin-resistant *Staphylococcus aureus* (MRSA). Dihydrotetrodecamycin 2 which lacks the *exo*-alkene, exhibits no antibacterial activity.^1,2^ Although the mechanism of antibacterial action remains unknown, it has been proposed that conjugate addition onto the exocyclic alkene by a functional cysteine residue on the target protein may be important for activity.^3^

The unusual structure of tetrodecamycin and its biological activity have attracted the attention of synthetic chemists and studies towards its synthesis have been reported.^3,4^ However, there has been only one total synthesis of tetrodecamycin 1 described in the literature,^5^ with analogues having also been prepared for SAR studies.^6^ In 2015, a further member of the tetrodecamycin family, 13-deoxytetrodecamycin 3, was isolated in low titres from extracts of *Streptomyces* WAC04657.^7^ The structure was investigated using spectroscopic methods and comparison with NMR data for tetrodecamycin. 13-Deoxytetrodecamycin 3 exhibits potent activity against Gram-positive organisms including MSRA and multi drug-resistant *S. aureus* ATCC BAA-44 in the range MIC 1 to 8 μg ml^−1^.^7^ There is an urgent need to develop new antibiotics against Gram-positive and Gram-negative pathogens and efficient methods for their synthesis are required. Hence our goal was to develop a flexible strategy for the total synthesis of the antibiotic 13-deoxytetrodecamycin 3.

**Scheme 2:**
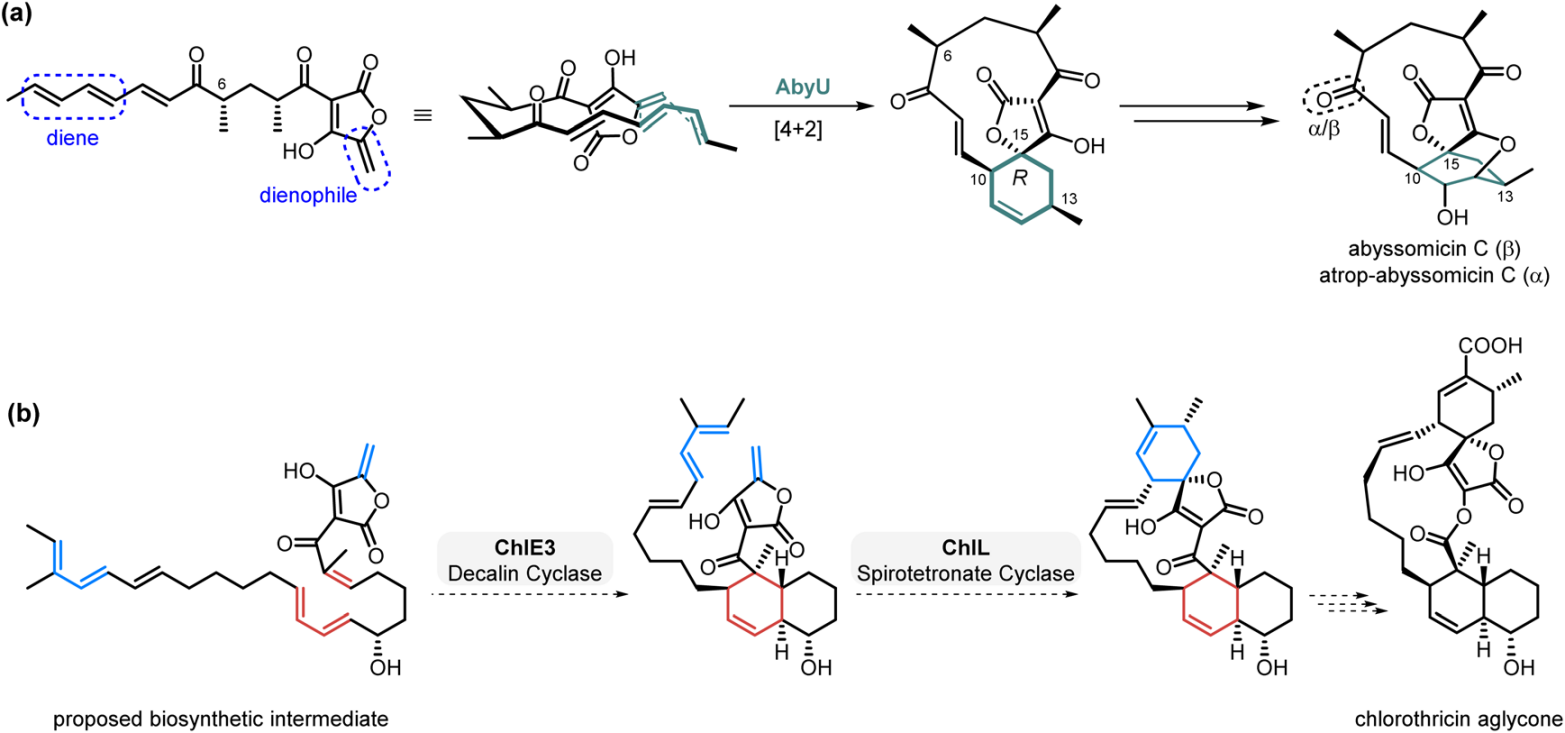
Diels–Alderases in spirotetronate biosynthesis. (a). Spirocyclisation in the biosynthesis of the antibiotic abyssomicin C catalysed by AbyU. (b). Proposed [4 + 2]-cycloadditions in chlorothricin biosynthesis.

An outline of our initially proposed synthetic strategy to 13-deoxytetrodecamycin 3 is shown in Scheme 1b. A key step is the selective intramolecular cycloaddition to convert 6 to the novel *trans*-decalin 7. The Diels–Alder reaction, involving the [4 + 2]-cycloaddition of a dienophile and conjugated diene, is one of the most valuable methods for the synthesis of six-membered rings generating up to four new stereocenters in a single step.^8^ This transformation commonly requires heat, pressure or Lewis acid catalysis, and achieving high enantioselectivity remains a challenge.^9^ It has long been speculated that Diels–Alder reactions are important in biosynthetic pathways forming structurally diverse natural products,^10^ but it is only recently that enzymes which catalyse [4 + 2]-cycloadditions have been isolated and characterised *e.g.* AbyU^11^ and AbmU^12^ in the biosynthesis of the class I spirotetronate antibiotics abyssomicin C and neoabyssomicin B respectively, as well as the Class II spirotetronates which contain an additional *trans*-decalin moiety *e.g.* versipelostatin^13^ and chlorothricin (Scheme 2).^14^

The tetrodecamycin biosynthetic gene cluster (BGC) has been identified, and Nodwell and co-workers proposed a biosynthetic route by analogy to pathways to other tetronate natural products in which the carbon skeleton is assembled *via* a type I polyketide synthase and the tetronate formed by a glycerol-derived C_3_ unit.^2,15^ Interestingly the tetrodecamycin pathway enzyme TedJ shows homology to previously identified Diels–Alderases^16,17^ and Nodwell and co-workers proposed that it may catalyse a [4 + 2]-cycloaddition to selectively form the decalin moiety. More recent studies by Jiao, Ge, Zhang and co-workers are in accord with this proposal and they reported that the cytochrome P450 TedH catalyses epoxidation, ether bridge formation and 13-hydroxylation in tetrodecamycin biosynthesis.^18^

Natural Diels–Alderases, including those found in spirotetronate biosynthetic pathways, have rarely been successfully deployed in the sustainable synthesis of bioactive compounds. This is despite their considerable potential to provide ‘green’, atom efficient routes to target molecules.^19^ Herein we describe the first total synthesis of 13-deoxytetrodecamycin 3, which has been achieved using a chemoenzymatic strategy incorporating the natural Diels–Alderase TedJ. Furthermore, structural, functional and computational studies have been used to provide a molecular framework for the TedJ catalysed reaction, establishing a foundation for future studies seeking to employ [4 + 2]-cyclases in asymmetric synthesis, accessed either *via* enzyme discovery, rational engineering, directed evolution, or a combination thereof.

## Results and discussion

### Synthesis of 6 and 7 and *in vitro* assays with TedJ

**Scheme 3:**
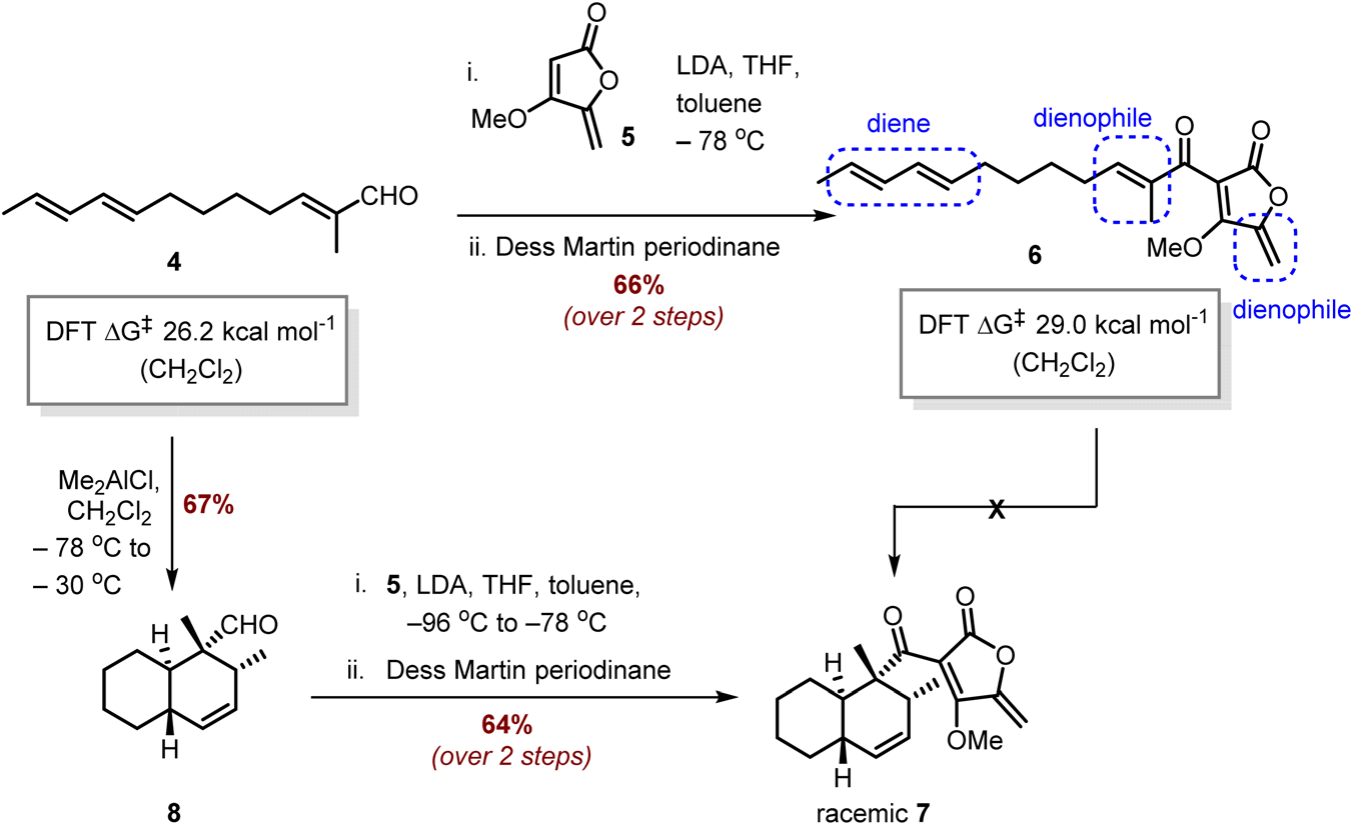
Synthesis of tetronate 6 and racemic cycloaddition product 7.

The first goal was to prepare tetronate 6 to investigate the proposed [4 + 2] cycloaddition. The *O*-methylated analogue was selected based on previous studies, which have shown that *O*-methyl tetronate analogues are substrates for the Diels–Alderase AbyU and obviate difficulties in characterising the corresponding tetronic acids which tautomerise.^11^ To begin aldehyde 4 was prepared *via* modification of previously described methods^4^ and coupled to tetronate 5. After extensive optimisation it was found that treatment of tetronate 5 with lithium diisopropylamide (LDA), then addition of aldehyde 4 at low temperature gave a mixture of diastereomeric alcohols, which on oxidation with Dess–Martin periodinane (DMP) delivered the required Diels–Alder substrate 6 in 66% yield over the two steps (Scheme 3). However, attempts to convert 6 to decalin 7 under standard conditions commonly used for Diels–Alder reactions, including heat and Lewis acids, were unsatisfactory leading to either recovered starting material or a complex mixture of products. Tetronate 6 has two electron deficient alkenes which may compete as dienophiles with the diene component in this Diels–Alder reaction and may account for the problems with this reaction. In contrast, the less sterically encumbered aldehyde 4 contains a single dienophile and may react more cleanly. Further insights into the potential Diels–Alder reactions were gained from density functional theory (DFT) calculations on non-catalysed reactions of both dienes 4 and 6 (details in the SI). Their ground and transition states were optimised at the M06-2X/6-31G(d) level, including implicit solvation in dichloromethane or water. Free energy barriers for cyclisation (calculated at the M06-2X/6-311+G(d,p) level) were 26.2 (CH_2_Cl_2_)/21.2 (H_2_O) kcal mol^−1^ for aldehyde 4, whilst the analogous barriers for tetronate 6 were 29.0 (CH_2_Cl_2_)/25.1(H_2_O) kcal mol^−1^.

Armed with this knowledge, the [4 + 2]-cycloaddition of unsaturated aldehyde 4 was investigated and our optimised reaction conditions were with Me_2_AlCl at low temperature giving *trans*-decalin 8 in 67% isolated yield (Scheme 3). These results were consistent with our DFT calculations that indicated a lower kinetic barrier for the conversion of 4 to 8 compared with cyclisation of 6 to 7. The structure of 8 was confirmed by NMR and was consistent with an *endo*-selective [4 + 2]-cycloaddition.^4*e*^ Coupling aldehyde 8 with tetronate 5 followed by oxidation of the resultant diastereomeric alcohols with DMP gave the novel *trans*-decalin 7 in 64% yield over the two steps.

Next, we turned our attention to the enzyme catalysed [4 + 2]-cycloaddition of 6. This biotransformation would be of particular value as it would be expected to provide a single enantiomer of 7 for use as an intermediate in the total synthesis of (−)-13-deoxytetradecamycin 3. Based on a comparative sequence analysis with known decalin forming [4 + 2]-cyclases, the *tedJ* gene of the *Streptomyces* WAC04657 tetrodecamycin biosynthetic gene cluster was identified as encoding a probable flavin dependent Diels–Alderase. This gene was subsequently cloned and an *N*-terminally hexa-histidine tagged variant of TedJ recombinantly over-expressed in *E. coli*, followed by purification to homogeneity (Fig. S1, SI). Purified TedJ was light yellow in colour and exhibited an absorbance spectrum consistent with that of a flavoprotein (Fig. S3, SI). Denaturation of TedJ by heating to 60 °C in MeOH for 30 min, followed by mass spectrometric (MS) analysis of the resulting material, revealed the presence of FAD (Fig. S4 and S5, SI), confirming its identity as a non-covalently bound cofactor in TedJ. Flavoenzymes which catalyse reactions without a change in the oxidation level of the cofactor have been reported^20,21^ and it seems likely that FAD in TedJ may play a solely structural role in the [4 + 2]-cycloaddition, as has been proposed for PyrE3, which catalyses decalin formation in pyrroindomycin biosynthesis.^16,17^

**Fig. 1:**
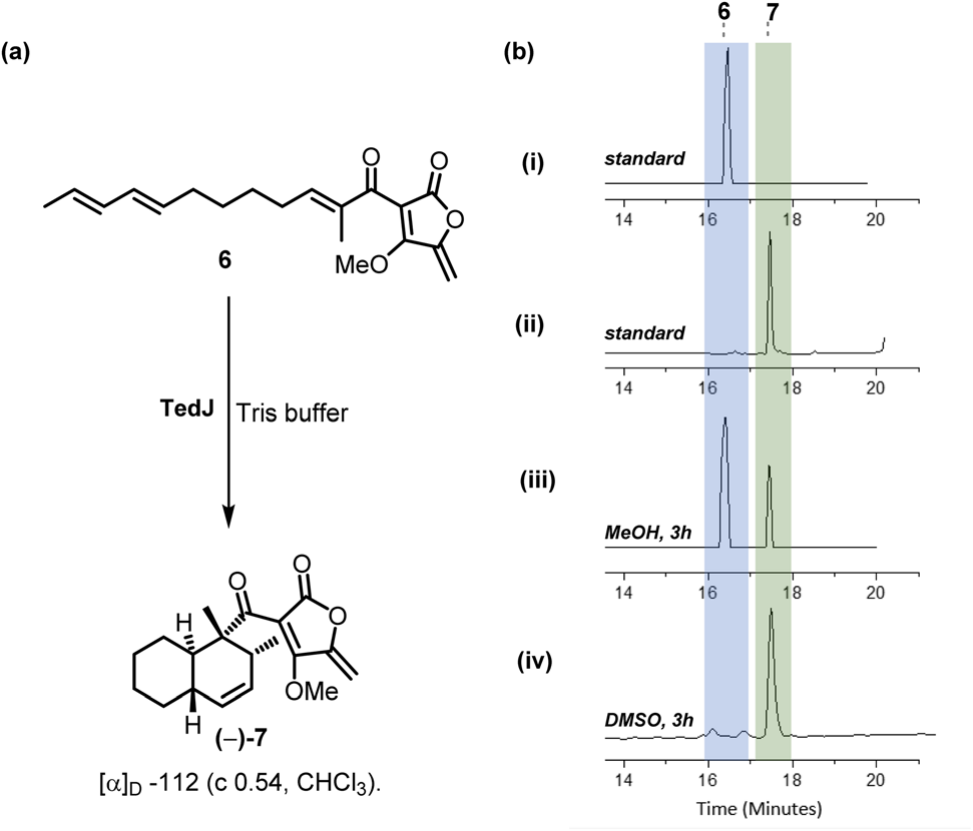
*In vitro* assays with substrate 6 and TedJ. (a) Cycloaddition of substrate 6 to *trans*-decalin (−)-7. (b) Reverse phase LC-MS chromatograms (i) synthetic standard of substrate 6; (ii) synthetic standard of product 7; (iii) analysis after 3 h using substrate 6 dissolved in MeOH; (iv) analysis after 3 h using substrate dissolved in DMSO.

For *in vitro* assays, tetronate 6 (0.2 mM) in 10% methanol co-solvent was incubated with purified recombinant TedJ at 25 °C in Tris-HCl buffer at pH 7.0 for 3 h. Analysis of the resultant mixture by reverse-phase LC-MS indicated the presence of starting material 6 and a new product with the same retention time as the synthetic standard 7 (Fig. 1). There was no evidence for cyclisation occurring in control experiments without enzyme. Under the same assay conditions except in DMSO co-solvent, complete consumption of substrate 6 with TedJ was observed after 3 h. These results are likely attributed to an improved substrate solubility in DMSO.

Without the need for any added cofactors, the *in vitro* reaction was scaled up using substrate 6 and TedJ and the product purified by flash column chromatography. NMR analysis confirmed that TedJ catalysed the intramolecular [4 + 2]-cycloaddition of 6, producing *trans*-decalin 7 (17 mg, 44% isolated yield) with the creation of two carbocyclic rings and four contiguous stereocenters, a transformation which failed in our hands under standard chemical conditions (heat or with Lewis acids). Importantly, not only had a single diastereomer been formed, but comparison of the product 7 with racemic material by chiral HPLC using a 0.2 : 99.8 EtOAc : hexane isocratic method confirmed that a single enantiomer, (−)-7, [*α*]_D_ −112 (*c* 0.54 in CHCl_3_) had been isolated from the enzymatic reaction (Fig. S6, SI).

### Total synthesis 13-deoxytetrodecamycin 3

**Scheme 4:**
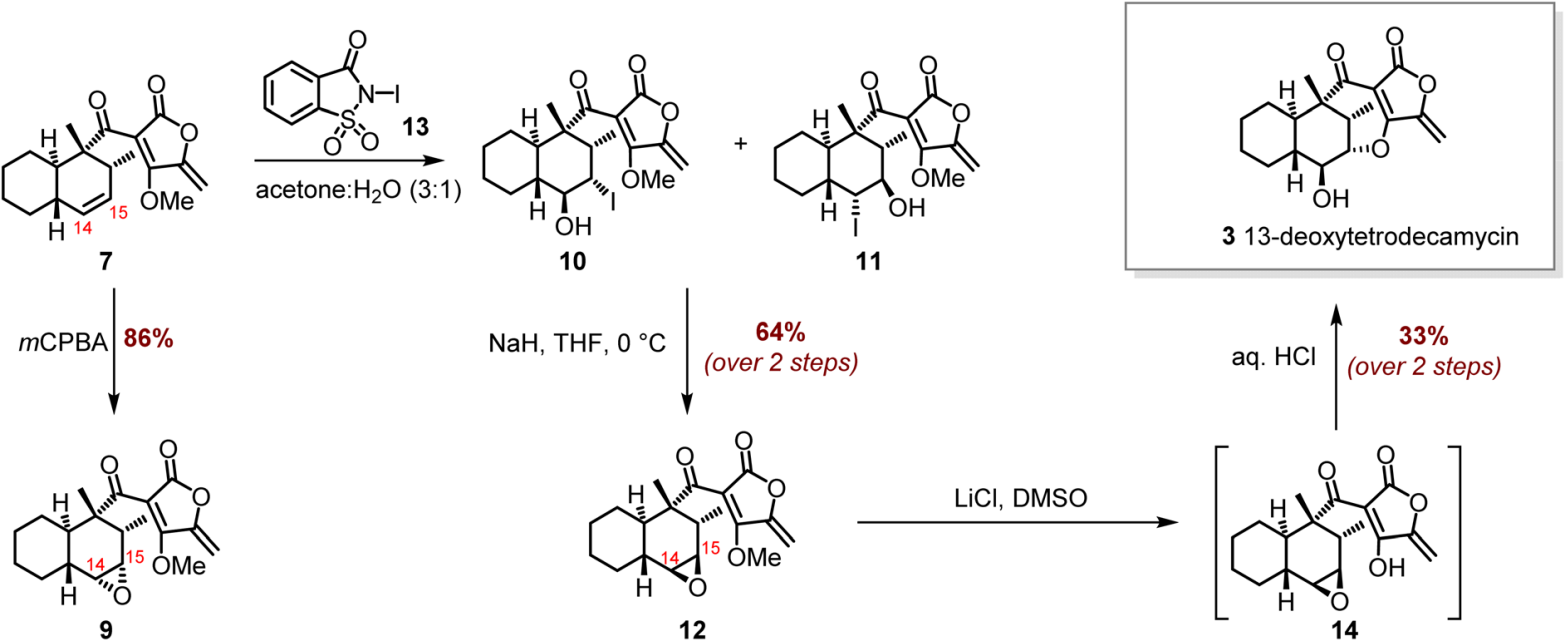
Completing the total synthesis of 13-deoxytetrodecamycin 3*via* selective epoxidation of *trans*-decalin 7.

To complete the total synthesis of 13-deoxytetrodecamycin 3 from Diels–Alder product 7, it was necessary to construct the 7-membered heterocycle uniting the decalin and tetronate rings and install the 14-hydroxyl group with the requisite stereochemistry. The synthetic route was initially optimised using racemic 7. The first step required a stereoselective epoxidation of the 14,15-alkene in *trans*-decalin 7 (Scheme 4). Treatment of 7 with *meta*-chloroperbenzoic acid (*m*CPBA) gave a single product with the correct mass for an epoxide ([C_19_H_24_O_5_+Na]^+^ 355.1532 Da). NMR data supported the selective epoxidation of the 14,15-alkene (Scheme 4) as the signals assigned to the alkene protons (*δ*5.50 and *δ*5.26) in the starting material 7 were no longer apparent in the product spectrum, and there were new signals at *δ*2.89 (1H, dd, *J* 5.5, 4.0) and 2.68 (1H, dd, *J* 4.0, 1.5) assigned to 15-H and 14-H respectively. However, further analysis of the spectral data along with molecular modelling studies indicated that epoxidation had occurred on the same face as the tetronate ring to form 9, *i.e.* a diastereomer of the intermediate required for the total synthesis of 13-deoxytetrodecamycin 3.

Thus, for the synthesis of the required 14,15-epoxide 12, we reasoned that as with epoxidation of alkene 7, an intermediate iodonium ion would also form selectively on the α-face of the molecule. Subsequent attack by water from the β-face would give vicinal iodohydrins 10 and 11, which in turn could be converted to the required epoxide 12. Treatment of 7 with widely used *N*-iodosuccinimide returned starting material, indicating that a more reactive electrophile was required. Hence, *N*-iodosaccharin 13 was prepared as previously described^22^ and on reaction with 7 gave a mixture of iodohydrins 10 and 11 which was used directly in the next step (Scheme 4). The crude mixture was treated with NaH, giving the required 14,15-epoxide 12 in 64% isolated yield over the 2 steps. The ^1^H-NMR spectrum of 12 displayed signals at *δ*2.77 (d, *J* 4.0) and *δ*2.51 (d, *J* 4.0) assigned to 15-H and 14-H respectively. Demethylation of the methyl ether in 12 using LiCl in DMSO gave 14, which was immediately treated with HCl completing the first total synthesis of 13-deoxytetrodecamycin 3. When *trans*-decalin (−)-7, obtained from the biotransformation with TedJ, was carried through this same sequence of epoxide formation-demethylation-cyclisation, it gave (−)-13-deoxytetrodecamycin 3. The ^1^H- and ^13^C-NMR data of the synthetic material were in accord with those reported for the isolated natural product (Table S4, SI) and the optical rotation [*α*]_D_ −14.0 (*c* 0.75 CHCl_3_) correlated well with that reported for the natural product [*α*]_D_ −17.6 (*c* 0.65, CHCl_3_).^7^

### Crystal structure of TedJ and mechanistic implications

**Fig. 2:**
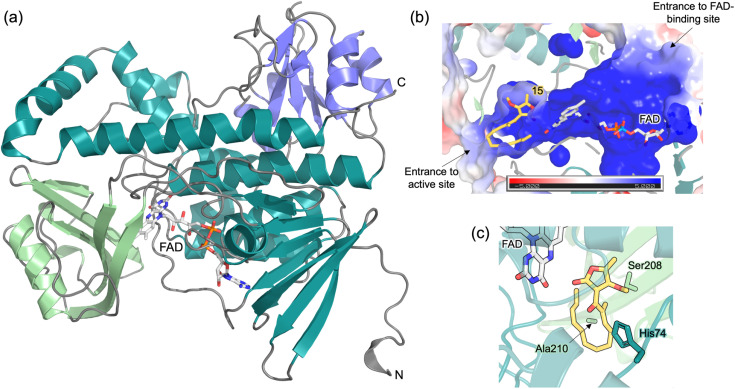
Crystal structure of TedJ. (a) Overall fold of Chain A shown in ribbon representation (PDB ID: 8 R1R). The FAD-binding domain is coloured teal, the central domain is pale green, and the C-terminal thioredoxin-like domain is blue. Loops are coloured dark grey. FAD is labelled and coloured pale grey and by atom. The location of the N and C termini of the protein are indicated. (b) Cut through view of TedJ coloured by electrostatic potential, showing the enzyme active site and non-covalently bound FAD molecule. Substrate 6 (not present in the crystal structure) is positioned as predicted by molecular docking studies and is coloured yellow, with FAD coloured pale grey. (c) View of the TedJ active site showing the predicted binding mode of 6. Residues His74, Ser208 and Ala210 are highlighted and labelled. 6 is coloured with yellow carbon atoms and FAD with pale grey carbon atoms.

To gain further insight into the TedJ catalysed [4 + 2]-cycloaddition, the crystal structure of this enzyme was determined to a resolution of 1.6 Å. Crystals of TedJ were grown from sitting-drop vapor diffusion experiments comprising purified recombinant TedJ supplemented with a crystal seed stock of ChlE3, a homologous protein from *Streptomyces antibioticus* (SI methods).^16^ The TedJ crystal structure was determined by molecular replacement using that of PyrE3 (PDB ID: 5XGV^17^) from the biosynthetic pathway of the pentacyclic tetramate pyrroindomycin as the search model. TedJ is homodimeric, with the dimer interface positioned along symmetry axes between individual asymmetric units within the crystal. The enzyme possesses a mixed α/β structure, consisting of three domains; an FAD-binding domain, a central domain and a C-terminal thioredoxin-like domain (Fig. 2a). Both the FAD-binding domain and the central domain are structurally analogous to those of the decalin-forming enzyme PyrE3, whereas the thioredoxin-like domain differs in the degree of loop and helical decoration of its central beta-sheet core. The enzyme active site is situated between the FAD-binding domain and the central domain (Fig. 2b) and resides at the base of a solvent exposed channel. Analysis using Foldseek^23^ identifies Tmn9, a 2-polyprenyl-6-methoxyphenol hydroxylase-like FAD-dependent oxidoreductase from *Streptomyces* sp. NRRL 11266 (PDB ID: 6UI5),^24^ as TedJ's closest structural homologue.

Molecular docking was used to provide further insights into the mechanism and selectivity of TedJ. The DFT optimised *endo*-transition state of 6 was docked into the TedJ crystal structure and the highest ranked docking result was relaxed using molecular mechanics energy minimisation with the Enlighten2 tools,^25^ yielding the likely reactive pose (Fig. 2b, see SI for further details). The pose of 6 in TedJ is similar to that reported for PyrE3. This is expected due to the significant sequence and structural similarities of TedJ and PyrE3, which each catalyse the formation of *trans*-decalins, albeit employing structurally distinct substrates.^17^ A number of protein–ligand interactions are conserved between the TedJ–6 and PyrE3 complexes. The tetronate and ketonic carbonyls of 6 were found to interact with His74, Ser208 and FAD in TedJ (Fig. 2c), analogous to the interactions of His74, Met201 and FAD in PyrE3 with its substrate. Notably, in this suggested reactive pose, the side-chain of Ala210 points towards the s-*cis* diene such that there is good stereo-complementarity (Fig. 2c). Inspection of the TedJ–6 complex reveals that steric constraints imposed by the side chains of residues H74, L77, R218, R262, and R358, appear critical in defining the stereochemical outcome of the TedJ catalysed reaction. The relative positioning of these residues, reinforced by the rigidity of the enzyme active site, would preclude the generation of alternative diastereomers or regioisomers, an observation fully in keeping with our *in vitro* assay data. Analogous to the proposed role of Ala45 in PyrE3, Ala210 would disfavour the orientation of the diene in an *exo*-selective manner. Thus, transition state docking and relaxation provide a mechanistic explanation for the stereoselectivity of the TedJ catalysed reaction.

## Conclusions

Biocatalysts are playing an increasingly important role in sustainable synthesis, but as noted by Reetz in 2013, it is of particular importance to develop the use of enzyme catalysed transformations which are difficult or impossible to achieve using non-biological methods.^26^ Herein we reveal that TedJ is a robust and efficient flavin-containing Diels–Alderase, which catalyses formation of the *trans*-decalin in tetrodecamycin biosynthesis *via* an intramolecular [4 + 2]-cycloaddition. TedJ was isolated, purified to homogeneity and its structure determined by X-ray crystallography. Its function was confirmed by *in vitro* assays using substrate 6, prepared in 2 steps and 66% overall yield from aldehyde 4. Interestingly, attempts to prepare *trans*-decalin 7*via* a chemical [4 + 2]-cycloaddition of 6 were unsuccessful under standard chemical conditions (heat or Lewis acids) generally employed for Diels–Alder reactions giving either no reaction or a complex mixture of products. In contrast, incubation of 6 with TedJ in buffer containing DMSO co-solvent at room temperature gave clean conversion to the enantiopure *trans*-decalin (−)-7 with complete stereocontrol.

It is proposed that FAD plays an exclusively structural role in TedJ, with no change in redox state during the reaction. Hence, without the need for additional cofactors, the TedJ-catalysed biotransformation was readily scaled up to generate *trans*-decalin (−)-7 as a single enantiomer and in sufficient amounts to complete the first total synthesis of the broad-spectrum antibiotic (−)-13-deoxytetrodecamycin 3. Stereoselective formation of epoxide 12*via* the precursor iodohydrins 10 and 11, followed by demethylation and acid-mediated cyclisation gave 3 with spectroscopic data and optical rotation value that correlated well with those of the natural product. These studies add a valuable new enzyme to the toolbox of biocatalysts for chemoenzymatic synthesis, unlocking a reaction with exquisite regio- and stereoselectivity under mild conditions, and facilitating access to a potent antimicrobial agent of potential clinical value. Elucidation of the X-ray crystal structure of TedJ, in combination with computational modelling, has provided important insights into the catalytic mechanism of this enzyme, establishing a foundation for the further development of this Diels–Alderase as a biocatalyst for use in synthesis. In future, the versatility and utility of TedJ may be further enhanced through structure guided rational enzyme engineering or directed evolution.

## Author contributions

M. A. H., M. W. v. d. K., P. R. R and C. L. W. supervised the experimental work and acquired funding. Protein studies including over-expression and purification of TedJ, protein crystallisation and X-ray crystallography was conducted by C. R. B., L. M., and M. M.-R.; synthetic work including spectroscopy and purification of TedJ for bioassays was carried out by S. J. R., C. P., N. R. L and S. N. C; computational studies were performed by K. A. C. and M. W. v. d. K. The manuscript was prepared with input from all the authors, led by P. R. R. and C. L. W.

## Conflicts of interest

There are no conflicts to declare.

## Supplementary Material

SC-OLF-D5SC05480J-s001

## Data Availability

The data supporting this article have been reported as part of the SI. Supplementary information is available and includes experimental procedures, analytical and spectral data, NMR spectra, computational methods, protein expression, purification, crystallisation and X-ray. See DOI: https://doi.org/10.1039/d5sc05480j.
