# Supplementary material for: Chemoenzymatic total synthesis of the antibiotic (−)-13-deoxytetrodecamycin using the Diels–Alderase TedJ
Source: Chem Sci. 2025 Aug 26;16(36):16993–9. doi: 10.1039/d5sc05480j (PMC12380092; doi:10.1039/d5sc05480j)
Supplement: SC-016-D5SC05480J-s001 [file SC-016-D5SC05480J-s001.pdf]

Supplementary Information (SI) for Chemical Science.
This journal is © The Royal Society of Chemistry 2025

Supporting Information for:

Chemoenzymatic Total Synthesis of the Antibiotic (–)-13-Deoxytetrodecamycin using the Diels-Alderase TedJ

S. Joe Russell, Catherine R. Back, Christopher Perry, Kaiman A. Cheung, Laurence Maschio, Sacha N. Charlton, Nicholas R. Lees, Martin A. Hayes, Marc W. van der Kamp, Monserrat Manzo-Ruiz

Paul R. Race and Christine L. Willis

Contents

1.Biological Procedures S3
 1.1. Gene Cloning S3
 1.2. Protein Expression & Purification S3
 1.3. Protein Crystallisation S5
 1.4. Diffraction Data Collection and Structure Determination S5
 1.5. TedJ Denaturation Assays S6
 1.6. LCMS Analysis S8
 1.7. Analytical Scale Enzymatic Assays S8
 1.8. Large Scale Enzymatic Assays S10
2. Computational Methods S11
 2.1. Conformational Searches and DFT S11
 2.2. Optimized Transition States S12
 2.3. Molecular Docking in TedJ S17
3. Synthetic Methods S17
 3.1. General Experimental S17
 3.2. Chemical Synthesis S18
4. Spectra S31
5. References S48

1. Biological Procedures

1.1. Gene Cloning

A DNA fragment corresponding to the *ted*J gene was PCR-amplified from commercially sourced synthetic DNA, codon optimized for *E. coli* (Eurofins MWG), using the primers AAGTTCTGTTTCAGGGCCCGATGACTGATCCGGTGATC (forward) and ATGGTCTAGAAAGCTTTAGCGCGGGGTTCCGAA (reverse). The PCR product was ligated into the vector pOPINF[1] using the In-Fusion™ system (Clontech) following the manufacturer's protocol. The resulting plasmid encodes an N-terminally hexa-histidine-tagged variant of TedJ. The construct was verified by DNA sequencing.

1.2. Protein Expression & Purification

The protein expression vector incorporating *ted*J was used to transform chemically competent *E. coli* BL21 (DE3) cells and grown at 37 °C on solid Luria-Bertani (LB) agar supplemented with carbenicillin. Single colonies were used to inoculate 10 mL liquid LB medium cultures supplemented with carbenicillin and grown overnight at 37 °C. Overnight cultures were used to inoculate 1 L LB medium supplemented with carbenicillin and the cells were grown at 37 °C, with shaking, to an $OD_{600\text{nm}}$ = 0.4–0.6. TedJ protein expression was induced by addition of IPTG (1 mM final concentration) followed by incubating the cultures at 20 °C for 16 h with shaking. Cells were harvested by centrifugation and resuspended in buffer (50 mM Tris, 150 mM NaCl, 20 mM imidazole, pH 8.0). Cells were lysed using a cell disruptor (Z Plus Series cell disruptor, Constant Systems Ltd.) at 25,000 psi and the lysate filtered through a 0.45 µm syringe filter. The lysate was applied to a 5 mL His-Trap HP chelating column (pre-loaded with nickel, GE Healthcare) and eluted with an imidazole gradient (20 – 500 mM) over 10 column volumes. Fractions were subjected to SDS-PAGE analysis and those containing recombinant TedJ protein were pooled, concentrated and then passed through a Hi-Load 16/60 Superdex 75 column (GE Healthcare) pre-equilibrated with buffer (20 mM Tris, 150 mM NaCl, pH 8.0). Fractions were analyzed by SDS-PAGE and those containing TedJ protein were pooled and concentrated to 10 mg/mL.

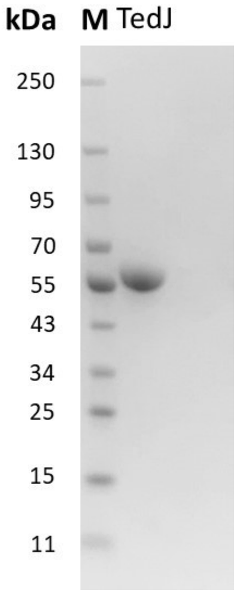

Figure S1. SDS-PAGE analysis of purified, recombinant TedJ

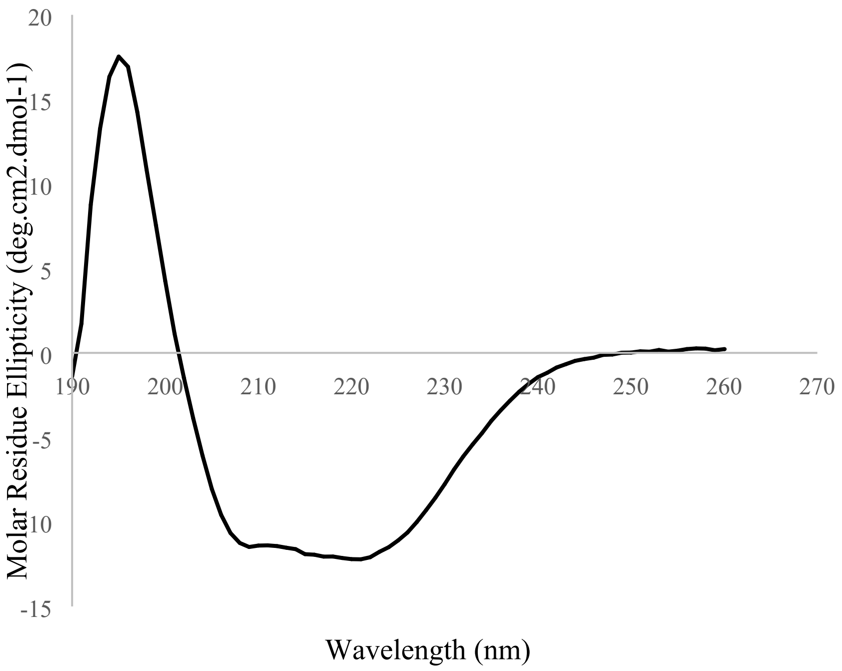

Figure S2. Circular dichroism spectra of purified, recombinant TedJ.

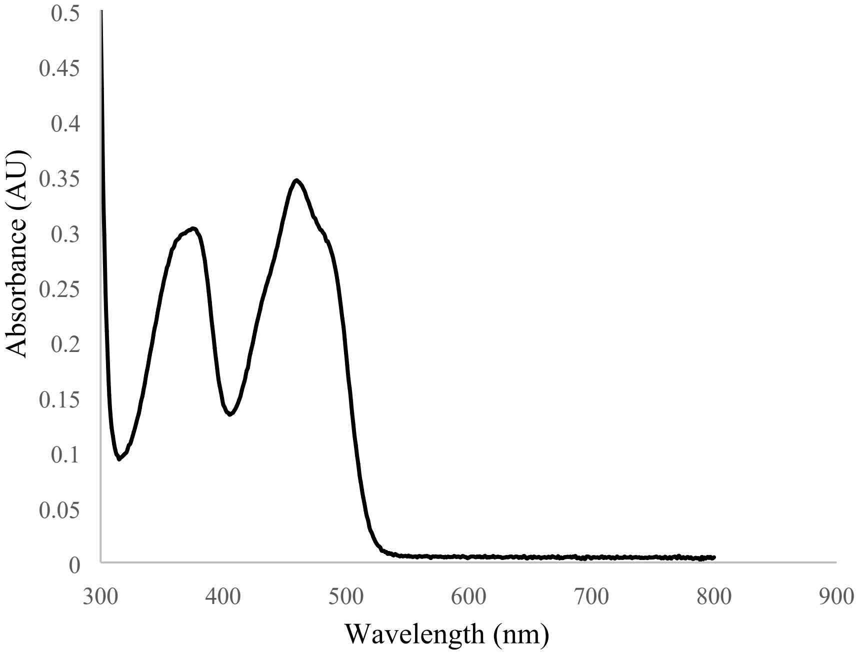

Figure S3. Spectrophotometric analysis of purified, recombinant TedJ.

1.3. Protein Crystallization

Conditions supporting the growth of crystals of TedJ were identified using the sitting drop method of vapor diffusion at 20 °C and commercially available screens (Molecular Dimensions), with the addition of a seed stock (1:100 dilution) of crushed crystals of ChlE3, a homologous protein from *Streptomyces antibioticus*[2] to a solution of TedJ at 10 mg/mL. Crystals took two months to grow. Diffraction quality crystals of TedJ were grown in 1.4 M sodium acetate trihydrate, 0.1 M sodium cacodylate, pH 6.5. Crystals selected for diffraction data collection were mounted in appropriately sized cryoloops (Hampton Research) and immediately flash-cooled in liquid nitrogen without additional cryoprotection prior to analysis.

1.4. Diffraction Data Collection and Structure Determination

Diffraction data were collected at Diamond Light Source, UK, on beamline I24. Data were auto-processed, merged and scaled with Xia2/3dii[3,4] using the Diamond Light Source Automatic Software Pipeline within the ISPyB system. The structure of TedJ was initially determined in space group $P4_12_12$, with one copy of the molecule in the asymmetric unit, to 1.6 Å resolution. The structure was determined using molecular replacement with the structure of PyrE3 (PDB ID: 5XGV)[5] as a search model, using MOLREP[6] as implemented in the CCP4i2 suite.[7] Iterative rounds of manual model building and refinement using COOT[8] and Refmac5[9] were used to refine the structure. Data collection, phasing, and refinement statistics for TedJ are provided in Table S1. Protein structure graphics were prepared using PyMOL Molecular Graphics System, Version 2.0 Schrödinger, LLC.

Table S1. Summary of X-ray data collection and refinement statistics.

	TedJ
**Data collection**	
Beamline wavelength (Å)	0.99987
Space group	$P4_12_12$
Cell dimensions	
a, b, c (Å)	70.25, 70.25, 203.7
a, b, g, (°)	90, 90, 90
Resolution (Å)	101.85-1.59 (1.62-1.59) [a]
R_{merge}	0.153 (4.83) [a]
No. of reflections	1756659 (73671) [a]
No. of unique reflections	69717 (3433) [a]
$I/\sigma I$	13.27 (0.8) [a]
$CC_{1/2}$	1.0 (0.358) [a]
Completeness (%)	100.0 (100.0) [a]
Redundancy	25.2 (21.46) [a]
**Refinement**	
R_{work}/R_{free}	0.207/0.244
No. of atoms	
Protein	3601
Ligand/ion	53
Water	171
B factors Å^2	
Protein	35.99
Ligand/ion	27.64
Water	36.19
Root mean square deviations	
Bond lengths (Å)	0.0095
Bond angles (°)	1.61
Ramachandran favoured (%)	98.6
Ramachandran outliers (%)	0.41

[a] Values in parentheses are for highest resolution shell

1.5. TedJ Denaturation Assays

TedJ (1 mg) was dissolved in HPLC grade MeOH (3 mL) and heated to 50 °C for 1 h. The resulting suspension was filtered through cotton wool, and the protein-free solution analyzed *via* reverse-phase HPLC-LCMS. A single peak (Figure S4a) was observed with a mass (ESI, m/z $[M+H]^+$ = 786) corresponding to FAD, as confirmed via comparison with a commercially sourced FAD standard analyzed using the same conditions (Figure S4, (b)).

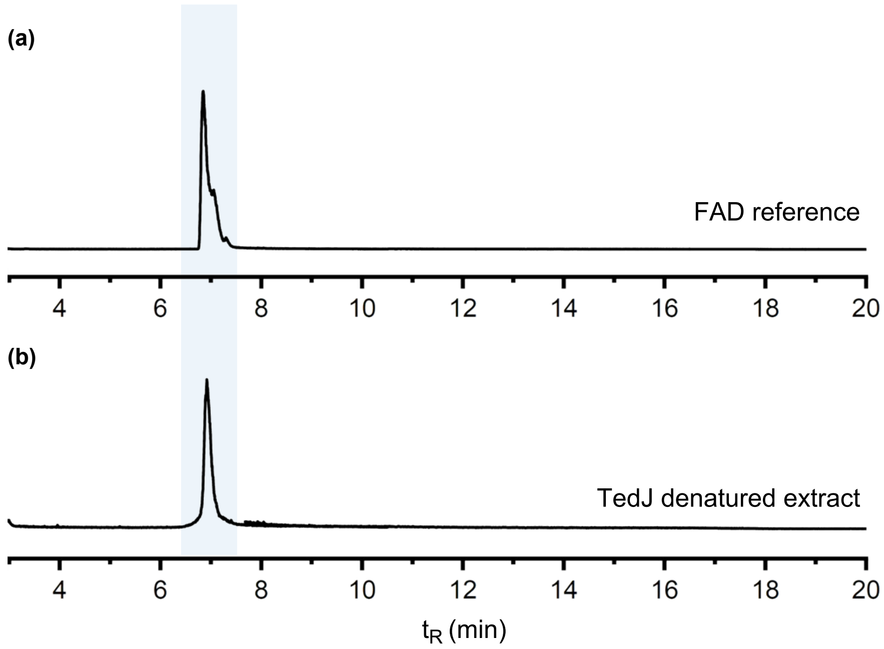

Figure S4. HPLC-MS traces FAD denaturation studies. (a) ELSD trace of commercial FAD standard (b) ELSD trace of denatured TedJ extract.

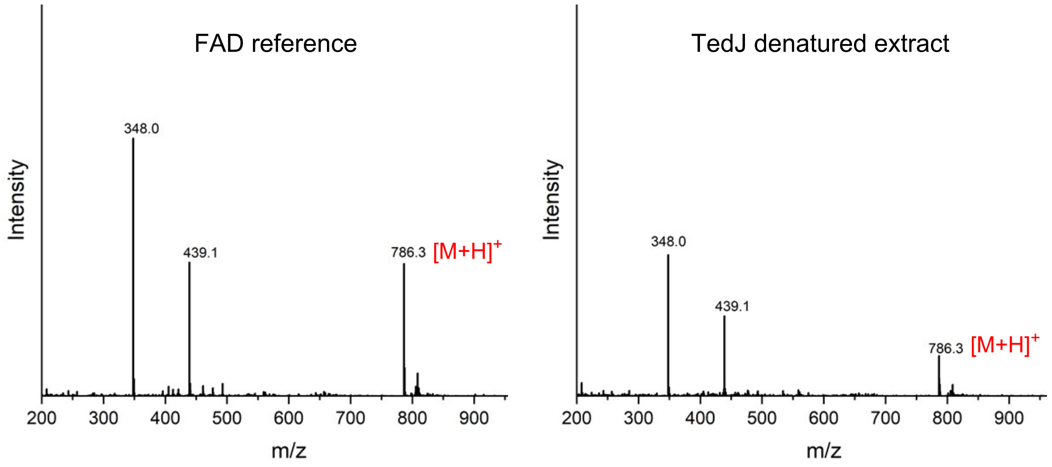

Figure S5. Mass spectra of FAD reference and TedJ denatured extract (ESI MS positive ion mode).

1.6. LCMS Analysis

Analytical samples were dissolved in MeOH and 10 µL was subjected to LCMS. LCMS analysis was conducted using a Waters 2795HT HPLC system incorporating a Waters 998 diode array detector (detection between 200 – 400 nm) and a Waters 2424 evaporative light scattering detector (ELSD). Mass spectrometry was carried out using an Electrospray (ES) Waters ZQ mass spectrometer with detection between 150 and 800 m/z units in negative and positive modes, Chromatography was carried out using a Phenomenex LUNA column (2.6 µm, C18, 100 Å, 4.6 × 250 mm) with a Phenomenex Security Guard column (Luna C5 300 Å). Samples were eluted at a flow rate of 1 ml min^{-1} with a gradient of 5 – 95 v/v% MeCN in H_2O (HPLC grade, both solvents supplemented with +0.04% formic acid) according to the following timepoints:

Table S2. Summary of HPLC method.

Time/minutes	Acetonitrile v/v%
**0 – 2**	5
**2 – 15**	5 – 95 linear gradient
**15 – 17**	95
**17 – 18**	95 – 5 linear gradient
**18 – 20**	5

1.7. Analytical Scale Enzymatic Assays

Assays were undertaken at a final volume of 200 µL comprising TedJ (40 µM), DMSO (10 µL), substrate (0.2 mM), Tris-HCl buffer (150 mM, pH 7.0). The reaction was incubated at room temperature for between 1 and 3 hours before addition of EtOAc (200 µL) and centrifugation. Negative controls were performed using the assay conditions described above but with the appropriate volume of buffer instead of enzyme. The organic layer was collected and added to an LCMS vial containing an insert before evaporation by nitrogen flow. The resulting residue was resuspended in HPLC grade MeOH for analysis.

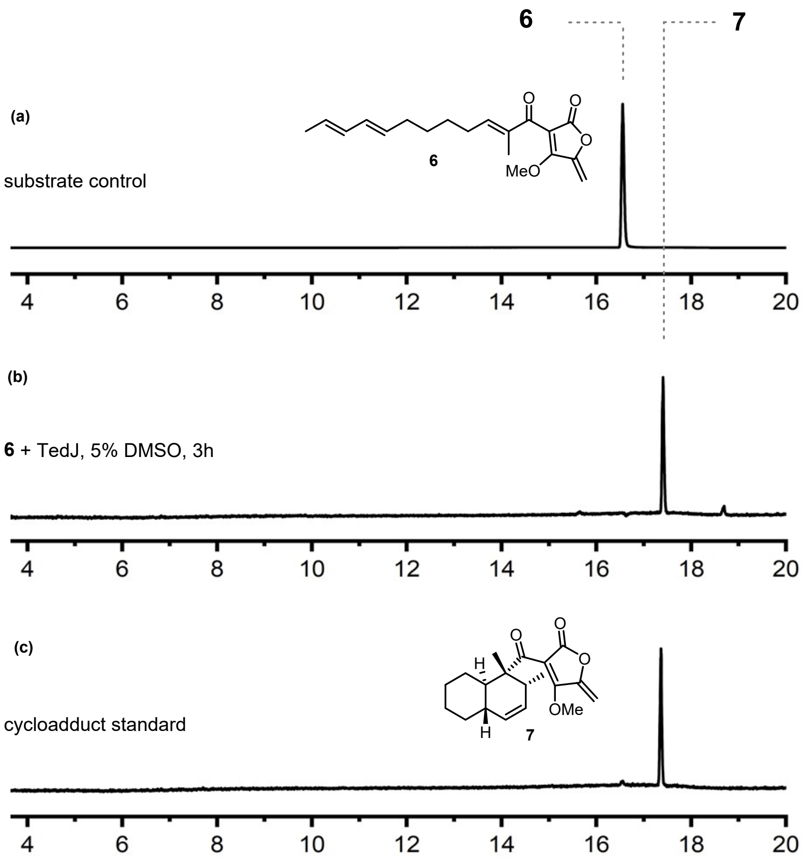

Figure S5. HPLC-MS traces for analytical enzymatic assays with substrate **15** and TedJ. (a) Standard of substrate **6**. (b) Enzyme assay with TedJ after 3 h. (c) Synthetic standard of cyclized product **7**.

1.8. Large Scale Enzymatic Assays

Large scale assays were undertaken at a final volume of 163.9 mL comprising TedJ (10 µM), substrate **6** (0.75 mM, 0.12 mmol), DMSO (5% by volume, 8195 µL), Tris-HCl buffer (50 mM, pH 7.0) and water. The reaction was incubated at 30°C with shaking at 25 rpm for 16 hours before the aqueous layer was extracted using EtOAc (4 × 40 mL). The organic phases were combined, dried over $MgSO_4$ and concentrated *in vacuo*. Purification by silica flash chromatography (10% EtOAc:petroleum ether 40 – 60) gave decalin **(−)-7** (17 mg, 44% yield) as a white solid $[\alpha]_D^{27}$ -112 (*c* 0.54, $CHCl_3$). Spectral data were in accord with synthetic racemate **7** prepared as described below. Starting material **6** (16 mg) was also recovered. Enantiopurity was determined through chiral HPLC analysis of the assay scale-up product, employing an Agilent Technologies 1260 Infinity HPLC. For chiral HPLC, an analytical (*R*,*R*)-WHELK-O 1 column (250 × 4.6 mm ID) was used, eluting with an isocratic method consisting of 0.8% EtOAc:Hexane (HPLC grade solvents) at a flow rate of 1 mL min^{-1}. Enantiopurity was determined by comparison of the integration values of racemic material (**Figure S6a**) against enzyme-cyclized product integration values.

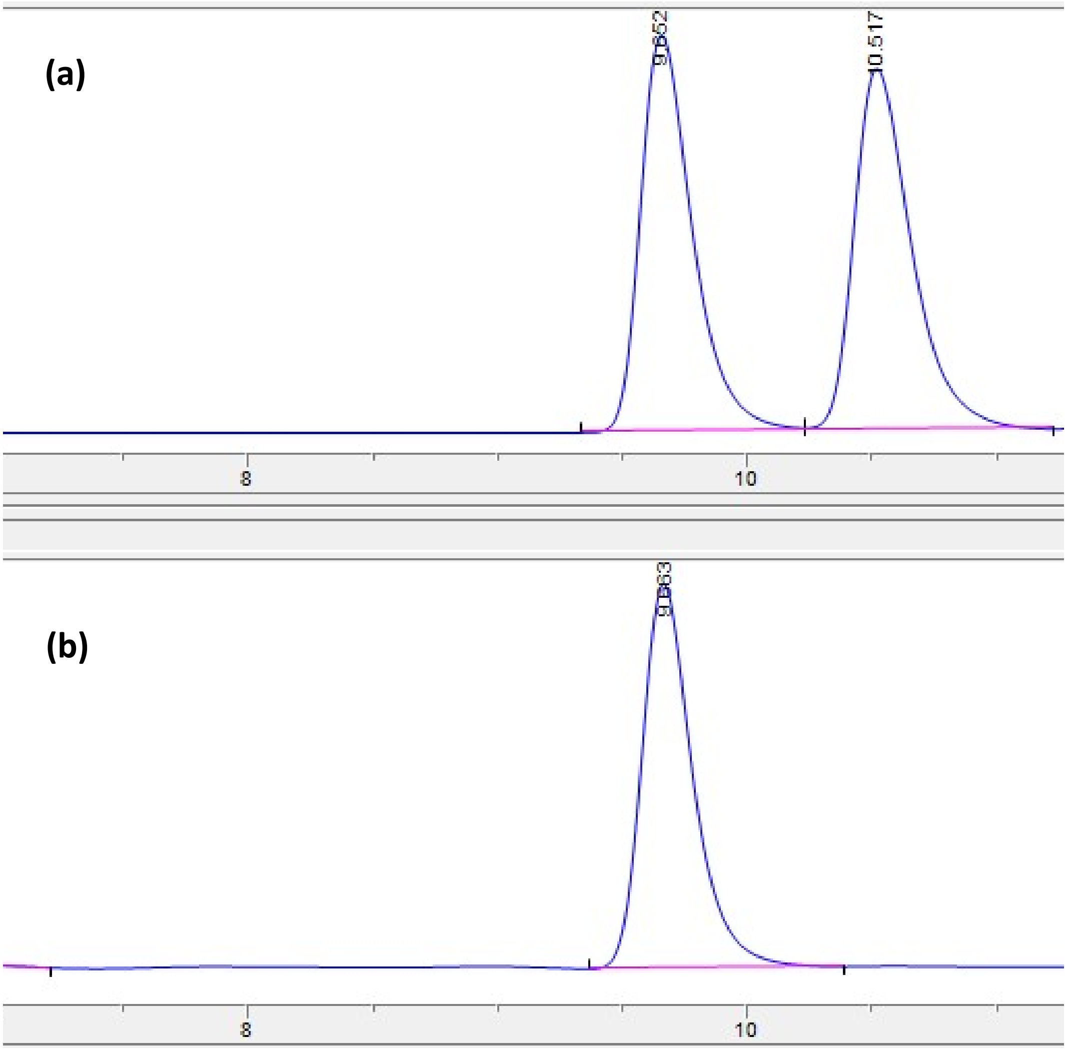

Figure S6. Chiral HPLC traces**.** (**a**) racemate **7** obtained by chemical synthesis. (**b**) enantiopure **7** as obtained by large scale enzymatic assay with TedJ.

2. Computational Methods

2.1. Conformational Searches and DFT

DFT calculations were carried out using the Gaussian 16 (Revision A.03) software package.[10] The M06-2X functional[11] was chosen due to its accuracy for describing molecular geometries and organic reactions, including Diels-Alder reactions.[12,13]

Initial structures of aldehyde **4** and tetronate **6** were generated in ChemDraw 22 and Chem3D, and briefly minimized using the built-in MM2 forcefield minimizer. Initial transition state guesses for **4** and **6** were obtained at the M06-2X/6-31g(d)/SMD(H_2O) level using the Berny optimization algorithm.[14] The resulting developing bond lengths (tetronate **6**: 1.971 and 2.774 Å, aldehyde **4**: 2.024 and 2.624 Å) were used for constrained conformational searches of transition states.

The initial precursor and transition state structures were subjected to conformational searches using Spartan 20 (MMFF forcefield, 10000 steps, retaining unique structures with an energy <40 kcal mol $^{-1}$) (Table S3).[15]

Table S3. Number of structures obtained during conformation searches of aldehyde **16**, tetronate **15** and their respective transition states.

Conformational Ensemble		Number of structures
Aldehyde 4	Starting material	370
	Transition state	13
Tetronate 6	Starting material	1590 (300*)
	Transition state	19

* For tetronate **15**, only the 300 lowest energy structures were carried forward.

The resulting conformational ensembles were reoptimized at the M06-2X/6-31g(d) level of theory incorporating Solvent Modelling by Density (SMD) in water or DCM, using the Berny optimization algorithm for transition states. Single-point energies of the resulting lowest energy structures were recalculated using the larger 6-311+g(d,p) basis set at the same level of theory. Free energies (298.15 K and 1 atmosphere) were evaluated according to thermochemical methods outlined by Ochterski.[16] Transition states were verified by frequency calculations, indicating the expected single imaginary frequency relating to the concerted formation of incipient C-C bonds in the Diels-Alder reaction. Reported energies in the body section are relative to the energy of the starting materials, whilst absolute energies in Hartree are reported in the supporting information.

2.2. Optimized Transition States

Tetronate 6
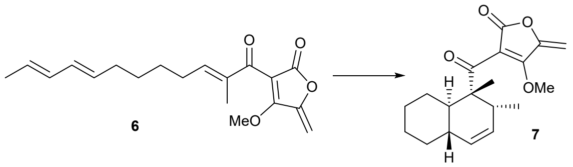

SMD(H_2O) 6	SMD(DCM) 6
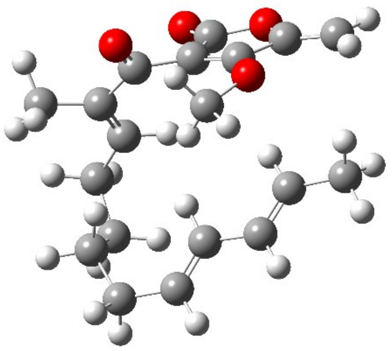	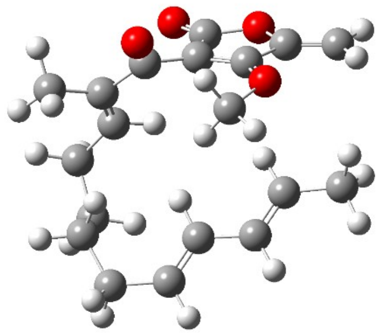

Electronic Energy (EE)	-1039.157675	Electronic Energy (EE)	-1039.169025
Zero-point Energy Correction	0.394284	Zero-point Energy Correction	0.394419
Thermal Correction to Energy	0.418007	Thermal Correction to Energy	0.418298
Thermal Correction to Enthalpy	0.418951	Thermal Correction to Enthalpy	0.419243
Thermal Correction to Free Energy	0.341924	Thermal Correction to Free Energy	0.341568
EE + Zero-point Energy	-1038.763391	EE + Zero-point Energy	-1038.774605
EE + Thermal Energy Correction	-1038.739668	EE + Thermal Energy Correction	-1038.750726
EE + Thermal Enthalpy Correction	-1038.738724	EE + Thermal Enthalpy Correction	-1038.749782
EE + Thermal Free Energy Correction	-1038.815751	EE + Thermal Free Energy Correction	-1038.827456

C -2.01853300 3.62006600 -0.43216000	C -1.93960100 3.68183500 -0.38539800
C -0.80347600 2.74828700 -0.41180800	C -0.74159100 2.78629000 -0.37585600
C 0.44126300 3.15201300 -0.12711600	C 0.51617700 3.16831500 -0.12010500
C 1.57606400 2.23276900 -0.08453800	C 1.63721100 2.23197500 -0.08725400
C 2.85323300 2.61123100 0.05918600	C 2.92188200 2.58932400 0.04096200
C 4.05343300 1.70482600 0.12302300	C 4.10694700 1.66261600 0. 10319200
C 3.75205900 0.21080400 0.00642400	C 3.78084100 0.17141200 0.01179600
C 3.36194500 -0.22549600 -1.40917600	C 3.37945000 -0.28677700 -1.39393700
C 2.69569400 -1.61378900 -1.43724900	C 2.69803900 -1.66807700 -1.39253200
C 1.36701800 -1.56791700 -0.74944500	C 1.36110200 -1.59788100 -0.71980000
C 1.04918500 -2.08711500 0.45091500	C 1.01183100 -2.10622200 0.47511600
C -0.28583600 -1.82132100 1.03363700	C -0.33470300 -1.82109200 1.03531400
C -1.37062900 -1.22388200 0.20297100	C -1.39606600 -1.20447700 0.18298400
O -0.51784600 -2.08557600 2.20926300	O -0.58817100 -2.07919700 2.20277300
C -2.23412700 -0.20957000 0.49034800	C -2.26036900 -0.19231600 0.46700500
C -3.23996700 -0.12932400 -0.57799700	C -3.25015200 -0.10187900 -0.61881000
O -2.97304400 -1.15128800 -1.47306800	O -2.97355800 -1.10432600 -1.51565300
C -1.84494400 -1.82625400 -1.04438400	C -1.85737100 -1.79538100 -1.08400000
O -1.42205100 -2.76418200 -1.67673800	O -1.43036000 -2.72364600 -1.71567500
C -4.22827300 0.74214300 -0.72897800	C -4.24026200 0.77033000 -0.76463000
C 1.97844900 -2.85142400 1.35183300	C 1.90725900 -2.88836200 1.39524600
O -2.28801000 0.70212100 1.43543000	O -2.33308000 0.71204600 1.41886700
C -1.17360400 0.84462400 2.33051000	C -1.24495600 0.83253700 2.34465000
H -2.55088100 3.53916900 -1.38704800	H -2.47685100 3.61715400 -1.33910800
H -2.72514400 3.31323000 0.34957400	H -2.64798000 3.38335100 0.39788800
H -1.75816800 4.66940400 -0.26514400	H -1.65928100 4.72560100 -0.21458200
H -0.95297400 1.69262200 -0.64725200	H -0.91955500 1.73209200 -0.59696700
H 0.63798300 4.20402100 0.08522600	H 0.73504900 4.21883900 0.07792100
H 1.32341300 1.17564200 -0.18533500	H 1.37028900 1.17772500 -0.17950300
H 3.06566300 3.67804800 0.14665900	H 3.15401300 3.65296500 0.11880000
H 4.76174300 2.00293500 -0.66392100	H 4.81232600 1.93790400 -0.69496700
H 4.57619500 1.90223900 1.06861000	H 4.64288000 1.86411300 1.04091300
H 4.63268700 -0.36168500 0.32016000	H 4.65380800 -0.40880400 0.33386400
H 2.95199200 -0.04508700 0.71358900	H 2.97911400 -0.06078600 0.72527800
H 2.68055000 0.50511800 -1.86034800	H 2.70488900 0.44383100 -1.85600600
H 4.25713200 -0.24903500 -2.04066500	H 4.27186500 -0.33055000 -2.02897200
H 2.53616300 -1.91097200 -2.48018900	H 2.54616700 -1.99147400 -2.42930700
H 3.36194100 -2.35137100 -0.98137800	H 3.35342100 -2.40118000 -0.91352400
H 0.60951200 -0.98688400 -1.27683700	H 0.61797700 -1.01617000 -1.26571400
H -4.37753200 1.51438800 0.01641600	H -4.39969900 1.53031100 -0.00900400
H -4.89559400 0.68877100 -1.58149400	H -4.90128500 0.72795200 -1.62291500
H 2.92680200 -3.07851400 0.86468100	H 2.86957700 -3.11768300 0.93652800
H 2.18752300 -2.27310200 2.25830200	H 2.08795900 -2.32614700 2.31765200
H 1.51689700 -3.79088400 1.67203900	H 1.42742100 -3.82773700 1.68824800
H -1.28388400 1.83343100 2.77269500	H -1.37460900 1.80364500 2.82077300
H -1.21243200 0.06988300 3.09681600	H -1.28817600 0.02811000 3.08012800
H -0.23569800 0.78600800 1.76976400	H -0.29197300 0.80490100 1.80641600

SMD(H_2O) 6-**TS**

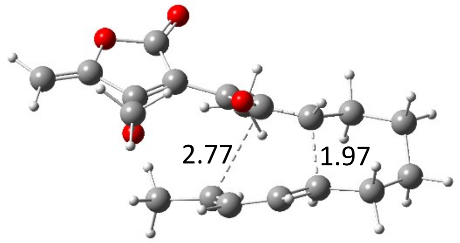

Electronic Energy (EE)	-1039.121230
Zero-point Energy Correction	0.394443
Thermal Correction to Energy	0.416531
Thermal Correction to Enthalpy	0.417475
Thermal Correction to Free Energy	0.345411
EE + Zero-point Energy	-1038.726787
EE + Thermal Energy Correction	-1038.704699
EE + Thermal Enthalpy Correction	-1038.703754
EE + Thermal Free Energy Correction	-1038.775818
Imaginary Frequency (cm^{-1})	-429.85

C	-0.86148800	0.95331800	0.01832200
C	-0.28923000	-1.07099600	1.82701200
C	-2.16694800	0.66162800	-0.43997800
C	-0.60731300	-2.02409100	0.90370500
H	-1.06067300	-0.39288300	2.18048900
C	-1.79915400	-1.99162000	0.14633100
C	-2.81167900	-1.04060500	0.31622100
C	-3.30385100	1.60120800	-0.07892500
H	-2.20384400	0.29216800	-1.46367300
C	-4.63532700	1.20154100	-0.71059500
C	-5.14006600	-0.13830600	-0.18420400
H	-5.37832300	1.98216200	-0.51524200
H	-4.51095200	1.14297400	-1.80023300
C	-4.13405200	-1.26619000	-0.39984000
H	-2.90924900	-0.61718200	1.31763700
H	-3.94174200	-1.39955100	-1.47271700
H	-5.34775500	-0.04826200	0.89079800
H	-6.08639600	-0.39905500	-0.66998800
H	0.14917600	-2.75624600	0.62784300
H	-1.89843900	-2.70331700	-0.67172200
C	0.18688700	0.28017000	-0.68645800
C	-0.63442100	1.94857300	1.12364900
H	-1.37241500	1.80923700	1.92159300
H	-0.74018900	2.98138600	0.76840500
H	0.35606700	1.84965000	1.57460400
O	-0.04872300	-0.58269800	-1.56378100
C	1.62060300	0.55419200	-0.37334500
C	2.60834900	-0.36209900	-0.28810900
C	2.26853800	1.86706700	-0.24344500
C	3.88110200	0.31426900	-0.03155200
O	2.50871500	-1.70017100	-0.34845700
O	1.81862100	2.98387400	-0.32882100
C	5.08302200	-0.20544000	0.18626900
H	5.94511200	0.42270900	0.37850400
H	5.20032600	-1.28333200	0.17611700
H	-3.41448600	1.66502400	1.01087600
H	-3.01986700	2.60604300	-0.41943500
O	3.62218500	1.67756700	-0.02785000
H	-4.56553200	-2.20759100	-0.03895100
C	1.01853400	-1.00437700	2.54544300
H	1.74891200	-1.70042100	2.12385500
H	1.43593900	0.00939900	2.50657800
H	0.87934200	-1.24326900	3.60749800

SMD(DCM) 6-**TS**

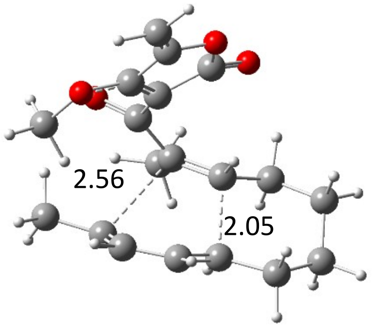

Electronic Energy (EE)	-1039.128400
Zero-point Energy Correction	0.395592
Thermal Correction to Energy	0.417367
Thermal Correction to Enthalpy	0.418311
Thermal Correction to Free Energy	0.347130
EE + Zero-point Energy	-1038.732808
EE + Thermal Energy Correction	-1038.711033
EE + Thermal Enthalpy Correction	-1038.710089
EE + Thermal Free Energy Correction	-1038.781270
Imaginary Frequency (cm^{-1})	-477.16

C	0.78280300	0.61713300	1.12958300
C	0.77753900	2.66218300	-0.41514400
C	1.49495000	-0.37275200	0.42307500
C	0.59732700	2.00535900	-1.61022300
H	1.73504100	2.56921800	0.08501900
C	1.39739400	0.92166200	-1.99939400
C	2.43504300	0.39628600	-1.23126800
C	2.68875500	-1.01222200	1.12148500
H	0.90326900	-1.08432200	-0.14405800
C	3.42903600	-2.06499700	0.30318800
C	4.18030300	-1.44859800	-0.87038600
H	4.13006500	-2.59526400	0.95754500
H	2.71106800	-2.81113900	-0.06496000
C	3.24065700	-0.73835500	-1.83861200
H	3.00147700	1.08048400	-0.59649700
H	2.54488100	-1.46848800	-2.27376000
H	4.91999200	-0.73154100	-0.48783600
H	4.73783000	-2.22060600	-1.41232600
H	-0.26781100	2.25353000	-2.22337900
H	1.08895000	0.35660400	-2.87889700
C	-0.67633600	0.63165500	1.17272300
C	1.46110600	1.39804600	2.22840700
H	2.47376700	1.70308700	1.94403600
H	1.54880900	0.79325600	3.14172100
H	0.88487800	2.29114100	2.48033100
O	-1.30697900	1.41922400	1.88515200
C	-1.45635900	-0.43878800	0.45299000
C	-2.61868300	-0.39120400	-0.25614300
C	-1.23848300	-1.86887400	0.74943200
C	-3.08140300	-1.76488700	-0.51226900
O	-3.36139800	0.57227200	-0.75955900
O	-0.40793200	-2.41343400	1.42843800
C	-4.13578000	-2.16628800	-1.21119600
H	-4.36952900	-3.21960000	-1.31615400
H	-4.77617700	-1.43080700	-1.68304500
H	3.39824500	-0.24183700	1.44702600
H	2.29217800	-1.47653800	2.03430300
O	-2.21708000	-2.61811400	0.12472800
H	3.82517200	-0.33103100	-2.67424200
C	-0.13156400	3.72713900	0.10218400
H	-0.85184500	4.04933400	-0.65682700
H	-0.69529500	3.35229000	0.97133400
H	0.43785200	4.60034700	0.43741900

C	2.84440900	-2.25188500	-1.63890100	C	-2.94327200	1.92707500	-0.56147300
H	2.14047400	-1.87958100	-2.38638300	H	-1.89669200	2.02652600	-0.85113200
H	3.86875300	-1.98506500	-1.91157000	H	-3.57630600	2.52972800	-1.21196200
H	2.75188200	-3.33170500	-1.53321300	H	-3.07090400	2.20861200	0.48452900

Aldehyde 4

SMD(H_2O) 4

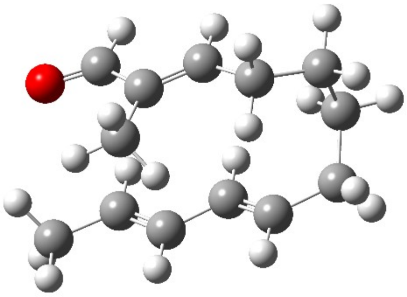

Electronic Energy (EE)	-582.493592
Zero-point Energy Correction	0.300028
Thermal Correction to Energy	0.315883
Thermal Correction to Enthalpy	0.316827
Thermal Correction to Free Energy	0.257823
EE + Zero-point Energy	-582.193564
EE + Thermal Energy Correction	-582.177709
EE + Thermal Enthalpy Correction	-582.176764
EE + Thermal Free Energy Correction	-582.235769

C 3.68601200 1.51139500 -0.02885100
C 2.27002100 1.43955600 -0.50648800
C 1.19759300 1.75788000 0.23035600
C -0.17578800 1.60374600 -0.23744600
C -1.25175400 1.80900700 0.53369800
C -2.69076600 1.63238600 0.11255800
C -2.96903900 0.50492000 -0.89350800
C -3.15221200 -0.87192400 -0.24743900
C -1.95653500 -1.38056500 0.56305400
C -0.72571100 -1.59861800 -0.26000700
C 0.53841600 -1.55060100 0.19867900
C 1.59571400 -1.80288100 -0.78518200
O 2.79382500 -1.81346300 -0.53694700
C 0.92423100 -1.24138700 1.61625200
H 4.15375600 0.51977000 -0.06279300
H 4.29069800 2.16737600 -0.66593200
H 3.73982300 1.88179600 0.99911300
H 2.11377700 1.07872700 -1.52473000
H 1.33438200 2.11022300 1.25461400
H -0.29037200 1.25822700 -1.26511500
H -1.08731100 2.15812700 1.55450400
H -3.30259900 1.46517400 100809600
H -3.04475400 2.58437700 -0.30692100
H -3.88895500 0.74287500 -1.43877300
H -2.17434600 0.46250700 -1.64780100
H -4.02659100 -0.83604600 0.41345100
H -3.38092700 -1.60431100 -1.03189800
H -2.22950900 -2.34250800 1.02186200
H -1.73467400 -0.70222500 1.39585600

SMD(DCM) 4

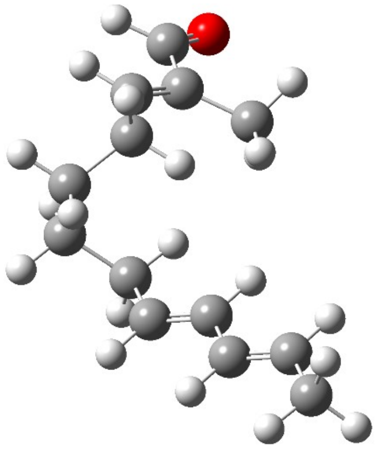

Electronic Energy (EE)	-582.506113
Zero-point Energy Correction	0.300072
Thermal Correction to Energy	0.316191
Thermal Correction to Enthalpy	0.317136
Thermal Correction to Free Energy	0.255693
EE + Zero-point Energy	-582.206041
EE + Thermal Energy Correction	-582.189921
EE + Thermal Enthalpy Correction	-582.188977
EE + Thermal Free Energy Correction	-582.250420

C -5.73089900 -1.31557200 0.52439600
C -4.28082600 -1.21857300 0.16584100
C -3.66443900 -0.09697400 -0.22734800
C -2.24815200 -0.02407100 -0.57501300
C -1.62973700 1.09274200 -0.98128700
C -0.17734100 1.18673600 -1.34327900
C 0.57193100 2.24804700 -0.52311100
C 0.49465400 2.03129400 0.99005000
C 0.96380500 0.63808400 1.44096100
C 2.36496500 0.35208800 1.00200700
C 2.81465000 -0.69747500 0.29320300
C 4.25714700 -0.71304900 -0.01758200
O 4.80435400 -1.59432000 -0.65023600
C 2.01951500 -1.86229600 -0.22047300
H -6.24766700 -2.03875300 -0.11798100
H -5.85922800 -1.66452600 1.55612800
H -6.23120500 -0.34785500 0.42396700
H -3.69748800 -2.13804200 0.23444200
H -4.23538800 0.83027300 -0.30169400
H -1.67710900 -0.95220200 -0.49935900
H -2.20812300 2.01711800 -1.05268300
H 0.29693700 0.20553700 -1.21474200
H -0.08118700 1.44581700 -2.40637400
H 1.62175000 2.25694200 -0.84160400
H 0.16410700 3.23871900 -0.75994300
H 1.10040800 2.79534600 1.49268800
H -0.53782000 2.17064400 1.33141300
H 0.27226700 -0.12629200 1.07485600
H 0.92908500 0.60084200 2.53806500

H -0.87999900 -1.83762700 -1.31495000	H 3.10119500 1.10707100 1.29087600
H 1.24396600 -1.99471300 -1.81629800	H 4.83726200 0.15018900 0.36684000
H 1.99494600 -1.03112400 1.68041100	H 2.43527900 -2.80024900 0.16287400
H 0.70051300 -2.08221900 2.28201000	H 2.08244600 -1.91398500 -1.31287800
H 0.37648300 -0.36818700 1.98682800	H 0.96800500 -1.80792000 0.06680400

SMD(H_2O) 4-**TS**	SMD(DCM) 4-**TS**
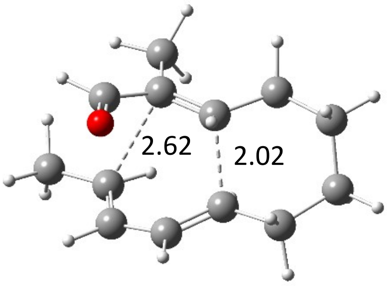	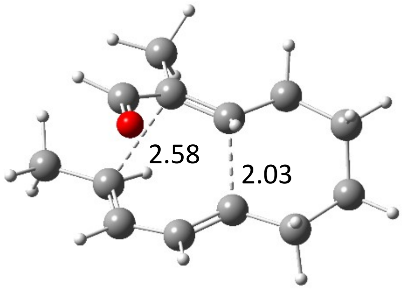

	SMD(H_2O) 4-**TS**	SMD(DCM) 4-**TS**
Electronic Energy (EE)	-582.464007	-582.470560
Zero-point Energy Correction	0.300923	0.300937
Thermal Correction to Energy	0.314901	0.314931
Thermal Correction to Enthalpy	0.315845	0.315875
Thermal Correction to Free Energy	0.261985	0.261962
EE + Zero-point Energy	-582.163085	-582.169623
EE + Thermal Energy Correction	-582.149107	-582.155629
EE + Thermal Enthalpy Correction	-582.148163	-582.154685
EE + Thermal Free Energy Correction	-582.202023	-582.208598
Imaginary Frequency (cm^{-1})	-449.59	-467.63

C	-0.67398700	1.20255200	0.27896300	C	-0.70323400	1.17762700	0.28413400
C	-2.00142700	-0.70671500	-0.93636100	C	-1.96970200	-0.70394200	-0.95151700
C	0.64118900	0.73378900	0.44497200	C	0.62001400	0.72946500	0.43020100
C	-1.58750900	-1.68285700	-0.06875700	C	-1.56740100	-1.69102400	-0.08909800
H	-1.28644200	-0.27052500	-1.62738600	H	-1.25051500	-0.27709400	-1.64416400
C	-0.23516500	-1.87699300	0.26666200	C	-0.21959000	-1.89288700	0.25402600
C	0.81283500	-1.14387500	-0.29170900	C	0.82689400	-1.15173800	-0.29532100
C	1.76883300	1.43180700	-0.29097800	C	1.73373400	1.44420100	-0.31178300
H	0.88405000	0.40947500	1.45612700	H	0.86791400	0.40402300	1.43975500
C	3.14700800	0.83844300	-0.00952800	C	3.12288700	0.87599400	-0.02620600
C	3.28517400	-0.58595900	-0.53819400	C	3.28876600	-0.55332200	-0.53577100
H	3.91403700	1.47697300	-0.46089500	H	3.87985600	1.52174800	-0.48529900
H	3.32406900	0.84386100	1.07454700	H	3.30053600	0.89887400	1.05777400
C	2.23983900	-1.53179800	0.04779100	C	2.25803100	-1.51347800	0.05538500
H	0.67180400	-0.77618100	-1.30932100	H	0.69213000	-0.79148700	-1.31642600
H	2.34837400	-1.58151700	1.13939500	H	2.36316500	-1.55028000	1.14808200
H	3.18312000	-0.57622300	-1.63202600	H	3.19376800	-0.55932900	-1.63064500
H	4.28630100	-0.97077200	-0.31616500	H	4.29665000	-0.91548200	-0.30453400
H	-2.34003300	-2.20764700	0.51753500	H	-2.32675500	-2.21180800	0.49200900
H	-0.00668200	-2.54420400	1.09687700	H	0.00202600	-2.55601700	1.08903200
C	-1.62719400	0.84092200	1.29171400	C	-1.62537900	0.80336100	1.33664300
C	-1.03936100	2.20462300	-0.77957600	C	-1.09777900	2.21186100	-0.73299200
H	-0.75617000	1.87061800	-1.78494600	H	-0.85271700	1.91119900	-1.75910500
H	-0.52909000	3.16192400	-0.61016200	H	-0.58073100	3.16400500	-0.55221900
H	-2.11659900	2.39842300	-0.77774700	H	-2.17356400	2.41079700	-0.68949000
O	-1.37012300	0.12712900	2.27450900	O	-1.33215200	0.08762400	2.29120900
H	1.57228200	1.42330100	-1.37120700	H	1.53831400	1.42595300	-1.39285000
H	1.75588700	2.48747800	0.01364900	H	1.70672000	2.50261900	-0.01663000
H	2.41990200	-2.54576800	-0.33077000	H	2.46040700	-2.52796400	-0.31154300
C	-3.44088700	-0.38498500	-1.18764700	C	-3.40817700	-0.38096900	-1.21654200
H	-4.08669400	-0.81070100	-0.41446300	H	-4.06045000	-0.79673300	-0.44283600
H	-3.60490400	0.69759400	-1.22344900	H	-3.57346200	0.70109200	-1.26520300
H	-3.75838600	-0.78139400	-2.16062700	H	-3.72332400	-0.78848300	-2.18621700
H	-2.64992700	1.24452600	1.16085100	H	-2.65713800	1.20247900	1.23389100

2.3. Molecular Docking in TedJ

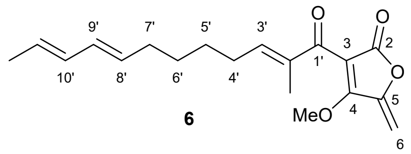

6

The crystal structure of TedJ was used for docking studies, together with the optimized transition state of **15**. During docking, bonds C(9')–C(10'), and the distance between C(3') and C(8') were treated as rigid, with all other formal single bonds treated as rotatable. Docking was performed using AutoDock Vina 1.2.3,[17] centered on a point halfway between the Cα atoms of residues Leu70 and Val206 using a grid of 16 × 16 × 18 Å with exhaustiveness set to 50. Side-chains of residues Leu70, His74, Leu77, Trp79, Leu97 and Ser208 were treated as flexible.

The highest ranked docked pose from AutoDock Vina (including flexible residues) was combined with the crystal structure of TedJ. The resulting complex was relaxed using the preparation and relaxation protocols from Enlighten2.[18] For FAD parameterisation, GAFF forcefield and topology files were generated for FAD using Antechamber from AmberTools20 (with AM1-BCC partial charges).[19,20]

3. Synthetic Methods

3.1. General Experimental

All reagents were sourced from commercial suppliers and used without further purification, with the exception of (+)-B-chlorodiisopinocampheylborane ((+)-DIP-Cl), which was dried over 3 Å molecular sieves. Moisture or air sensitive reactions were carried out in oven or flame dried glassware under a positive pressure of nitrogen using standard Schlenk syringe-septa techniques. Anhydrous solvents THF, Et_2O and CH_2Cl_2 were dried by passing through a modified Grubbs system of alumina columns manufactured by Anhydrous Engineering. All stated temperatures below ambient are the temperatures of cooling baths, unless otherwise specified.

Routine reaction monitoring was achieved using thin layer chromatography on aluminum backed silica plates (Merck DC Alufolien Kieselgel 50 F_{254}). Detection of chemical species was enabled through irradiation with a UV light source (λ_{max} 254 & 366 nm) or through staining using potassium permanganate solution (5 %) as a reactive stain. Flash column chromatography was performed using 40-63 µm silica gel as the stationary phase (Fischer Scientific or Aldrich), with a suitable eluent.

^1H and ^{13}C NMR spectra were recorded using a Jeol JNM-ECS 400 (Jastec Magent), Bruker Nano 400, Jeol Jeol JNM-ECZ 400 (Varian magnet), Varian VNMRS 500 and Bruker Neo 600 Cryo spectrometer at ambient temperature. Chemical shifts (δ) are quoted in parts per million (ppm) with coupling constants (*J*) quoted in Hertz (Hz) and are referenced to the residual solvent peak for both proton and

carbon chemical shifts. ^{1}H NMR is reported in the following format: chemical shift/ppm, integration, multiplicity (s = singlet, d = doublet, t – triplet, q = quartet, m = multiplet, br = broad), coupling constant (*J*), assignment. Optical rotation ([α_D]) was measured using a Bellingham and Stanley Ltd ADP220 polarimeter irradiating with sodium D line (λ=589 nm) with all values quoted in (° mL)(g dm)$^{-1}$. Melting points were determined using an Electrothermal IA6301 melting point apparatus. Samples for mass spectrometry were submitted in MeOH or CH_2Cl_2. Mass spectra were recorded on an Apex IV 7 Tesla FT-ICR (Electrospray ionization) or VG Analytical Quattro mass spectrometer (electron ionization and chemical ionization), with methane used as the reagent gas for chemical ionization.

3.2. Chemical Synthesis

1-(*tert*-Butyldimethylsilyoxy)-3-iodopropane (SI-3)

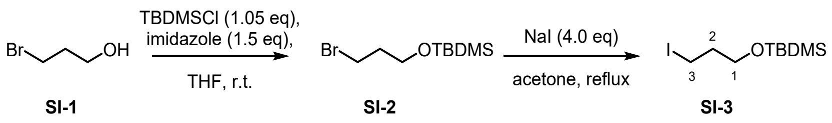

Under an atmosphere of nitrogen, 3-bromopropan-1-ol **SI-1** (1.50 mL, 16.6 mmol, 1 eq) was dissolved in CH_2Cl_2 (30 mL) and cooled to 0 °C. TBDMSCl (2.63 g, 17.4 mmol, 1.05 eq) and imidazole (1.69 g, 24.9 mmol, 1.5 eq) were added sequentially, the reaction stirred at 0 °C for 30 minutes before warming to room temperature for a further hour. The reaction was quenched with sat. aq. NH_4Cl (40 mL) and extracted with CH_2Cl_2 (3 × 25 mL). The combined organic layers were washed with brine (20 mL), dried over $MgSO_4$, and concentrated *in vacuo* to give a quantitative yield of protected alcohol **SI-2** which was used immediately used in the subsequent Finkelstein reaction.

Sodium iodide (9.95 g, 66.4 mmol, 4 eq) was added to a solution bromide **SI-2** (4.20 g, 16.7 mmol, 1 eq) in acetone (25 mL) and the solution was heated to reflux for 4 h. Following cooling, the solvent was removed *in vacuo*. The residue was dissolved in CH_2Cl_2 (40 mL), washed with brine (3 × 20 mL), dried over $MgSO_4$ and concentrated *in vacuo* to give **SI-3** as a yellow oil (4.96 g, 99% yield). **δ_H** (400 MHz, $CDCl_3$) 3.67 (2H, t, *J* 5.5, 1-H_2), 3.28 (2H, t, *J* 6.5, 3-H_2), 1.99 (2H, app. pent., *J* 6.0, 2-H_2), 0.89 (9H, s, SiC(CH_3)$_3$), 0.07 (6H, s, Si(CH_3)$_2$). **δ_C** (100 MHz, $CDCl_3$) 62.4 (C-1), 36.2 (C-3), 26.1 (SiC(CH_3)$_3$), 18.4 (Si*C*(CH_3)$_3$), 3.9 (C-1), -5.2 (Si(CH_3)$_2$).

All data are in accordance with the literature.[21]

(3*E*,5*E*)-1-Bromohepta-3,5-diene (SI-6)

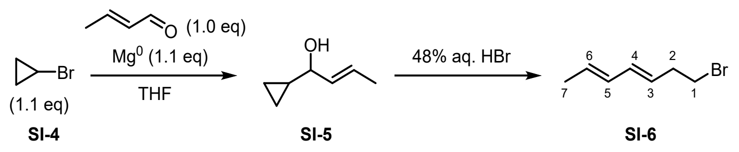

Under an atmosphere of nitrogen, a solution of bromocyclopropane **SI-4** (500 mg, 4.14 mmol, 1.1 eq) in THF (2.5 mL) was added dropwise to magnesium turnings (100 mg, 4.14 mmol, 1.1 eq) in THF (1.5 mL) at a rate to maintain a gentle reflux. The mixture was stirred at room temperature for 30 min, prior to cooling in an ice bath to 0 °C. Crotonaldehyde (0.31 mL, 3.76 mmol, 1 eq) in THF (2.5 mL) was added dropwise to the Grignard reagent over 20 minutes. The resulting mixture was stirred and allowed to warm to room temperature over a further hour before addition of sat. aq. NH_4Cl (5 mL). The aqueous solution was extracted with Et_2O (3 ×10 mL), the organic layers washed with brine (3 × 3 mL), dried over $MgSO_4$ and concentrated *in vacuo* to give crude alcohol **SI-5**, which was used in the next step without further purification.

Alcohol **SI-5** was cooled to 0 °C in an ice bath before concentrated aq. HBr (48 % HBr, 0.6 mL) was added dropwise with vigorous stirring over 5 minutes. The reaction was monitored *via* TLC (25% EtOAc:hexane), and the reaction was shown complete after a further 10 minutes. The solution was diluted with H_2O (5 mL) and extracted with pentane (4 x 5 mL). The combined organic layers were washed sequentially with sat. aq. $NaHCO_3$ (2 × 6 mL) and brine (2 × 6 mL) before drying over $MgSO_4$. The slurry was filtered through silica plug in pentane and concentrated *in vacuo* at 0 °C to produce bromide **SI-6** as a clear oil (478 mg, 73% yield as a 9:1 ratio of 3*E*:3*Z* isomers). This mixture was used in the next stages of the synthesis and subsequently this *E,Z*-ratio was maintained until tetronate-coupled **15** was purified by $AgNO_3$-impregnated silica flash column chromatography. **δ_H** (400 MHz, $CDCl_3$) 6.13-5.98 (2H, m, 4-H and 5-H), 5.71-5.61 (1H, m, 6-H), 5.55-5.46 (1H, m, 3-H), 3.38 (2H, *J* 7.0, 1-H_2), 2.61 (2H, app. q, *J* 7.0, 2-H_2), 1.74 (3H, d, *J* 7.0, 7-H_3). **δ_C** (100 MHz, $CDCl_3$) 133.3 and 131.2 (C-4 and C-5), 129.1 (C-6), 127.5 (C-3), 36.1 (C-2), 32.6 (C-1), 18.2 (C-7).

All data are in accordance with the literature.[22]

(6*E*,8*E*)-1-(*tert*-Butyldimethylsilyloxy)deca-6,8-diene (SI-7)

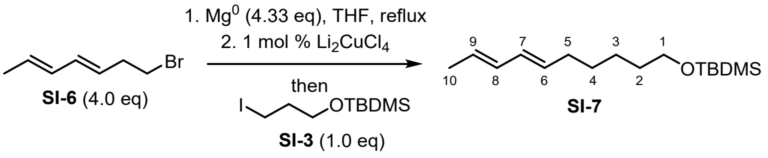

Under an atmosphere of nitrogen, a solution of bromodiene **SI-6** (2.00 g, 11.5 mmol, 4 eq) in THF (10 mL) was added dropwise to freshly milled magnesium turnings (300 mg, 12.4 mmol, 4.33 eq) and I_2 (one small crystal) in THF (10 mL). The solution was heated to reflux for 30 min until complete consumption of magnesium was observed and then cooled to 0 °C (10 minutes cooling), after which a solution of Li_2CuCl_4 (0.1 M in THF, 1.15 mL, 0.115 mmol, 0.04 eq) was added dropwise over 5 minutes. The mixture was stirred at 0 °C for a further 10 minutes, before addition of alkyl iodide **SI-3** (857 mg,

2.85 mmol, 1 eq). The reaction mixture was stirred at 0 °C for 2 h, after which a color change was observed from gray to gray-red. Sat. aq. NH_4Cl (40 mL) was added and the aqueous layer subsequently extracted with Et_2O (3 × 30 mL). The combined organic layers were washed with brine (30 mL), dried over $MgSO_4$ and concentrated *in vacuo* to yield crude diene. Purification by column chromatography (1% EtOAc:Petroleum ether 40 – 60) gave diene **SI-7** as a colorless oil in (667 mg, 87% yield as a 9:1 mixture of 6*E*:6*Z* isomers). $\boldsymbol{\delta_H}$ (400 MHz, $CDCl_3$) 6.06-5.93 (2H, m, 7-H and 8-H), 5.62-5.50 (2H, m, 9-H and 6-H), 3.59 (2H, t, *J* 6.5, 1-H_2), 2.05 (2H, app. q, *J* 7.0, 5-H_2), 1.73 (3H, d *J* 6.5, 10-H_3), 1.51 (2H, pent., *J* 7.0, 2-H_2), 1.39-1.30 (4H, m, 3-H_2 and 4-H_2), 0.89 (9H, s, $SiC(CH_3)_3$), 0.04 (6H, s, $Si(CH_3)_2$). $\boldsymbol{\delta_C}$ (100 MHz, $CDCl_3$) 132.2 (C-6), 131.8 (C-8), 130.5 (C-7), 126.9 (C-9), 63.4 (C-1), 32.9 (C-2), 32.7 (C-5), 29.4 (C-4), 26.1 (C-3), 25.5 ($SiC(CH_3)_3$), 18.5 ($SiC(CH_3)_3$), 18.2 (C-10), -5.1 ($Si(CH_3)_2$). **HRMS** (APCI) calc. $[C_{16}H_{31}OSi + H]^+$ 269.2295, observed 269.2283.

(6*E*,8*E*)-Deca-6,8-dien-1-ol (SI-8)

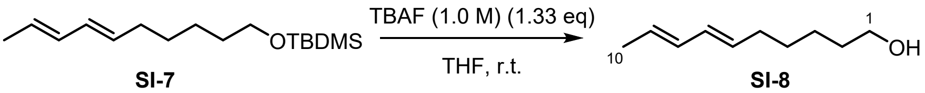

Under an atmosphere of nitrogen, TBDMS ether **SI-7** (631 mg, 2.35 mmol, 1 eq) was dissolved in THF (14 mL). Tetrabutylammonium fluoride (1 M in THF, 3.12 mL, 3.12 mmol, 1.33 eq) was added at 0 ˚C. The reaction mixture was allowed to warm to room temperature and stir for 16 h. Sat. aq. NH_4Cl solution (60 mL) was added and the aqueous layer was extracted with Et_2O (3 × 30 mL). The combined organic extracts were washed with brine (2 × 20 mL), dried over $MgSO_4$, filtered and concentrated *in vacuo*. Purification by column chromatography (10% EtOAc:petroleum ether 40 – 60) gave dienol **SI-8** as a colourless oil (356 mg, 98% yield). $\boldsymbol{\delta_H}$ (400 MHz, $CDCl_3$) 6.06-5.91 (2H, m, 7-H and 8-H) 5.62-5.48 (2H, m, 6-H and 9-H), 3.64 (2H, dt, *J* 10.5, 3.0, 10-H_2) 2.07 (2H, app. q, *J* 7.0, 5-H_2), 1.73 (3H, d, *J* 6.6, 10-H_3), 1.57 (2H, pent, *J* 6.9, 2-H_2), 1.45-1.31 (4H, m, 3-H and 4-H), 1.21 (1H, t, *J* 5.1, CH_2OH). $\boldsymbol{\delta_C}$ (100 MHz, $CDCl_3$) 131.9 (C-6), 131.8 (C-8), 130.6 (C-7), 127.1 (C-9), 63.1 (C-1), 32.8 (C-2), 32.6 (C-5), 29.3 (C-4), 25.4 (C-3), 18.2 (C-10). **IR** (ν_{max}/cm^{-1}) (neat) 3319, 3014, 2927, 2855, 1626. **HRMS** (APCI) calc. $[C_{10}H_{18}O + H]^+$ 155.1430, observed 155.1424.

All data are in accordance with the literature.[23]

(6*E*,8*E*)-Deca-6,8-dienal (SI-9)

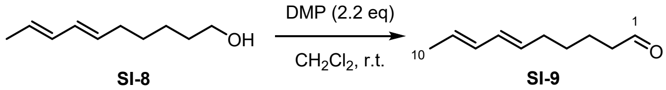

Under an atmosphere of nitrogen, alcohol **SI-8** (358 mg, 2.32 mmol, 1 eq) was dissolved in CH_2Cl_2 (20 mL). Dess-Martin Periodinane (DMP) (2.16 g, 5.10 mmol, 2.2 eq) was added and the resulting mixture stirred at room temperature for 2 h. A mixture of 10 m/v% aq. $Na_2S_2O_3$ and sat. aq. $NaHCO_3$ (1:1, 15 mL) was added to the reaction mixture then extracted with CH_2Cl_2 (3 ×20 mL). The combined organic layers were washed with brine (20 mL), dried over $MgSO_4$ and concentrated *in vacuo*. Purification by column chromatography (2% EtOAc:petroleum ether 40 – 60) gave aldehyde **SI-9** as a colorless oil (300 mg, 85% yield as a 6:1 mixture of 6*E*:6*Z* isomers). $\boldsymbol{\delta_H}$ (400 MHz, $CDCl_3$) 9.76 (1H, t, *J* 2.0, 1-H), 6.05-5.92 (2H, m, 7-H and 8-H), 5.63-5.47 (2H, m, 6-H and 9-H), 2.42 (2H, td, *J* 7.5, 2.0, 2-H_2), 2.08 (2H, app. q, *J* 7.0, 5-H_2), 1.73 (3H, d, *J* 6.5, 10-H_3), 1.64 (2H, app. pent., *J* 7.5, 3-H_2), 1.46-1.39 (2H, m, 4-H_2). $\boldsymbol{\delta_C}$ (100 MHz, $CDCl_3$) 202.9 (C-1), 131.6 (C-8), 131.2 (C-9), 131.0 (C-6), 127.4 (C-7), 43.9 (C-2), 32.3 (C-5), 29.0 (C-4), 21.7 (C-3), 18.2 (C-10). **IR** (ν_{max}/cm^{-1}) (neat) 3015, 2927, 2855, 2718, 1723. **HRMS** (APCl) calc. $[C_{10}H_{16}O + H]^+$ 153.1274, observed 153.1270.

All data are in accordance with the literature.[24]

Ethyl (2*E*,8*E*,10*E*)-2-methyldodeca-2,8,10-trienoate (SI-10)

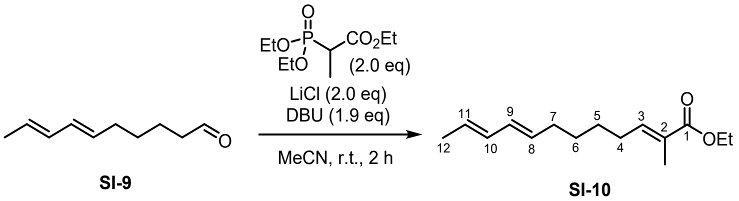

Under an atmosphere of nitrogen, lithium chloride (77 mg, 1.81 mmol, 2 eq) was added to a flame-dried flask then heated under vacuum for 10 minutes until completely dry. Triethyl 2-phosphonopropionate (0.39 mL, 1.81, 2 eq) in MeCN (10 mL) was added followed by DBU (0.26 mL, 1.72 mmol, 1.9 eq) added and the resultant solution stirred for 10 minutes at room temperature. Aldehyde **SI-9** (138 mg, 0.91 mmol, 1 eq) in anhydrous MeCN (5 mL) was added and the reaction mixture stirred for 2 h, following which TLC analysis showed reaction completion. Sat. aq. NH_4Cl solution (20 mL) was added and extracted with Et_2O (4 × 10 mL). The combined organic phase was washed with brine (15 mL), dried over $MgSO_4$ and concentrated *in vacuo*. Purification by column chromatography (2% EtOAc:petroleum ether 40 – 60) gave dienoate **SI-10** as a yellow oil (152 mg, 72% yield). $\boldsymbol{\delta_H}$ (400 MHz, $CDCl_3$) 6.74 (1H, tq, *J* 7.5, 1.5, 3-H), 6.06-5.94 (2H, m, 10-H and 11-H), 5.62-5.48 (2H, m, 11-H and 8-H), 4.18 (2H, q, *J* 7.0, OCH_2CH_3), 2.16 (2H, app. q, *J* 7.0, 4-H_2), 2.06 (2H, app. q, *J* 7.0, 7-H_2), 1.82 (3H, s, 13-H_3), 1.73 (3H, d, *J* 7.0, 12-H_3), 1.50-1.38 (4H, m, 5-H_2 and 6-H_2), 1.29 (3H, t, *J* 7.0, OCH_2CH_3). $\boldsymbol{\delta_C}$ (100 MHz, $CDCl_3$) 168.5 (C-1), 142.3 (C-3), 131.71 (C-10), 131.68 (C-11), 130.7 (C-8), 127.9 (C-2), 127.2 (C-9), 60.5 (OCH_2CH_3), 32.5 (C-7), 29.2 (C-4), 28.7 (C-5), 28.2 (C-6), 18.1 (C-12), 14.4

(OCH_2CH_3). **IR** (ν_{max}/cm^{-1}) (neat) 2928, 2856, 1709, 1650. **HRMS** (ESI) calc. $[C_{15}H_{24}O_2 + Na]^+$ 258.1669, observed 259.1673.

All data are in accordance with the literature.[23]

(2*E*,8*E*,10*E*)-2-Methyldodeca-2,8,10-trien-1-ol (SI-11)

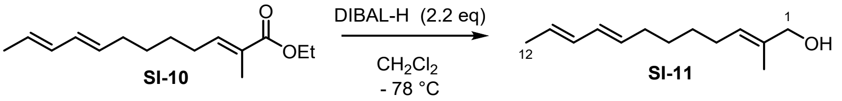

Under an atmosphere of nitrogen, ester **S-10** (152 mg, 0.64 mmol, 1 eq) was dissolved in CH_2Cl_2 (5 mL) and cooled to -78 ˚C. DIBAL-H solution (1.42 mL, 1.42 mmol, 2.2 eq, 1.0 M in hexane) was added dropwise and the solution stirred for 1 h. The reaction was quenched by slow dropwise addition of 1 M HCl (4.9 mL) and diluted with Et_2O (10 mL). The layers were separated, and the aqueous layer further extracted with Et_2O (3 × 10 mL), combined organics washed with brine (15 mL), dried over $MgSO_4$ and concentrated *in vacuo*. Purification by column chromatography (25% EtOAc:petroleum ether 40 – 60) gave alcohol **SI-11** as a colorless oil (108 mg, 86% yield). $\boldsymbol{\delta_H}$ (400 MHz, $CDCl_3$) 6.06-5.91 (2H, m, 9-H and 10-H), 5.62-5.49 (2H, m, 11-H and 8-H), 5.40 (1H, t, *J* 7,0, 3-H), 4.00 (2H, d, *J* 5.0, 1-H_2), 2.09-1.98 (4H, m, 4-H_2 and 7-H_2), 1.73 (3H, d, *J* 7.0, 12-H_3), 1.66 (3H, s, 2-CH_3), 1.44-1.42 (4H, m, 5-H_2 and 6-H_2), 1.26 (1H, s, CH_2OH). $\boldsymbol{\delta_C}$ (100 MHz, $CDCl_3$) 134.8 (C-2), 132.0 (C-8), 131.8 (C-10), 130.5 (C-9), 127.0 (C-11), 126.6 (C-3), 69.2 (C-1), 32.6 (C-7), 29.21 and 29.15 (C-5 and C-6), 27.6 (C-4), 18.1 (C-12), 13.8 (C-13). **IR** (ν_{max}/cm^{-1}) (neat) 3324, 3016, 2923, 2854, 1670.

All data are in accordance with the literature.[23]

(2*E*,8*E*,10*E*)-2-Methyldodeca-2,8,10-trienal (4)

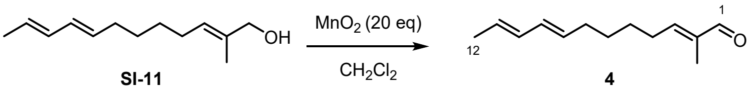

Under an atmosphere of nitrogen, alcohol **SI-11** (203 mg, 1.03 mmol, 1 eq) was dissolved in CH_2Cl_2 (15 mL). MnO_2 (1.81 g, 20.9 mmol, 20 eq) was added portionwise and the reaction stirred overnight at room temperature. The reaction mixture was filtered through a silica plug, eluting with CH_2Cl_2, then concentrated *in vacuo*. Purification by column chromatography (2% EtOAc:petroleum ether 40 – 60) gave aldehyde **4** as a yellow oil (166 mg, 83% yield). $\boldsymbol{\delta_H}$ (400 MHz, $CDCl_3$) 9.39 (1H, s, 1-H) 6.48 (1H, td, *J* 7.5, 1.5, 3-H), 6.06-5.91 (2H, m, 9-H and 10-H), 5.65-5.48 (2H, m, 8-H and 11-H), 2.35 (2H, app. q, *J* 7.0, 4-H_2), 2.09 (2H, app. q, *J* 7.0, 7-H_2), 1.74 (3H, s, 2-CH_3) 1.73 (3H, d, *J* 7.0, 12-H_3), 1.55-1.41 (4H, m, 5-H_2 and 6-H_2). $\boldsymbol{\delta_C}$ (100 MHz, $CDCl_3$) 195.5 (C-1), 154.5 (C-3), 139.8 (C-2), 131.7 (C-8), 131.3 (C-10),

130.9 (C-9), 127.4 (C-11), 32.4 (C-7), 29.2 and 29.0 (C-5 and C-6), 28.0 (C-4), 18.2 (C-12), 9.3 (C-13). **IR** (ν_{max}/cm^{-1}) (neat) 3014, 2924, 2855, 2708, 1686, 1644.

All data are in accordance with the literature.[23]

5-((Dimethylamino)methylene)-4-methoxyfuran-2(*5H*)-one (SI-13)

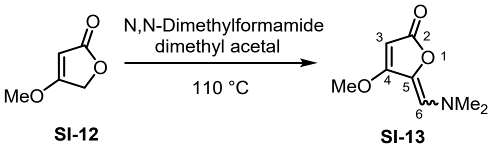

Under an atmosphere of nitrogen, 4-methoxy-2(5*H*)-furanone **SI-12** (2.00 g, 17.5 mmol, 1 eq) was dissolved in *N*,*N*-dimethylformamide dimethyl acetal (12.5 mL) and heated to 110 °C, with removal of the MeOH via distillation. After 6 h, the reaction mixture was cooled and concentrated *in vacuo*. Purification by column chromatography (50% EtOAc:petroleum ether 40 – 60) gave lactone **SI-13** as a pale-yellow solid (2.84 g, 96% yield). **δ_H** (400 MHz, $CDCl_3$) 6.03 (1H, s, 6-H), 4.88 (1H, s, 3-H), 3.84 (3H, s, OCH_3), 3.09 (6H, s, $N(CH_3)_2$). **δ_C** (100 MHz, $CDCl_3$) 171.5 (C-2), 170.1 (C-4), 122.8 (C-6), 120.4 (C-5), 80.8 (C-3), 58.6 (OCH_3), 42.6 ($N(CH_3)_2$). **HRMS** (ESI) calc. $[C_8H_{11}NO_3 + H]^+$ 170.0812, observed 170.0809.

All data are in accordance with the literature.[25]

5-((Dimethylamino)methyl)-4-methoxyfuran-2(*5H*)-one (SI-14)

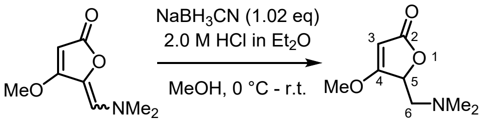

Under an atmosphere of nitrogen, lactone **SI-13** (500 mg, 2.96 mmol, 1 eq) was dissolved in MeOH (3 mL) and cooled to 0 °C. HCl (2.0 M in Et_2O, 0.11 mL) was added (color change observed pale yellow to pale blue) followed by addition of $NaBH_3CN$ (190 mg, 3.2 mmol, 1.02 eq) in four portions (color change observed, pale blue to bright yellow-green). The solution was maintained acidic (*ca* pH 2) *via* further addition of HCl in Et_2O (*ca* 3 mL) for 15 minutes, monitored using pH paper, affording a white suspension. The mixture was stirred at room temperature for an additional hour. Excess solvent was removed *in vacuo* and the white residue dissolved in H_2O (5 mL) before basification to pH 12 with NaOH (5.0 M). The aqueous layer was extracted with EtOAc (5 × 15 mL), the combined organic layers dried over $MgSO_4$ and concentrated *in vacuo* to afford amino-lactone **SI-14** as a yellow oil (410 mg, 81% yield). **δ_H** (400 MHz, $CDCl_3$) 5.09 (1H, d, *J* 1.0, 3-H), 4.88 (1H, dd, *J* 7.5, 1.0, 5-H), 3.89 (3H, s, OCH_3), 2.90 (1H, dd, *J* 14.0, 2.5, 6-*H*H), 2.50 (1H, dd, *J* 14.0, 7.5, 6-H*H*), 2.35 (6H, s, $N(CH_3)_2$). **δ_C** (100 MHz,

CDCl$_3$) 181.1 (C-2), 172.5 (C-4), 89.4 (C-3), 78.7 (C-5), 60.6 (C-6 or OCH$_3$), 59.6 (C-6 or OCH$_3$), 46.2 (N(CH$_3$)$_2$). **HRMS** (ESI) calc. [C$_8$H$_{13}$NO$_3$ + H]$^+$ 172.0968, observed 172.0971.

All data are in accordance with the literature.[26]

4-Methoxy-5-methylenefuran-2(*5H*)-one (5)

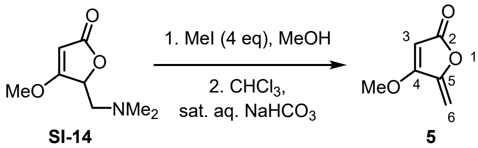

Under an atmosphere of nitrogen, amine **SI-14** (606 mg, 3.54 mmol, 1 eq) was dissolved in MeOH. MeI (0.89 mL, 14.2 mmol, 4 eq) was added over 5 minutes and the reaction stirred overnight at room temperature. The solvent was removed *in vacuo* before addition of CHCl$_3$ (5 mL) and sat. aq. NaHCO$_3$ solution (7 mL) and the reaction was stirred for additional 2 h. The mixture was diluted with H$_2$O (10 mL), the aqueous layer washed with CH$_2$Cl$_2$ (4 × 10 mL). The organic layers were combined, washed with brine (10 mL), dried over MgSO$_4$ and concentrated *in vacuo* to afford crude product. Purification by column chromatography (25% EtOAc:petroleum ether 40 – 60) gave tetronate **5** as a white crystalline solid (407 mg, 91% yield). **δ$_H$** (400 MHz, CDCl$_3$) 5.25 (1H, dd, *J* 1.5, 0.5, 3-H), 5.07 (1H, dd, *J* 2.5, 1.5, 6-H*H*), 5.04 (1H, dd, *J* 2.5, 0.5, 6-*H*H), 3.93 (3H, s, OCH$_3$). **δ$_C$** (100 MHz, CDCl$_3$) 169.9 (C-2), 168.4 (C-4), 149.8 (C-5), 92.6 (C-6), 90.1 (C-3), 59.4 (OCH$_3$). **MS** (ESI) calc. [C$_6$H$_6$O$_3$ + H]$^+$ 126.0, observed 126.0.

All data are in agreement with the literature.[27]

4-Methoxy-3-((2'E,8'E,10'E)-2'-methyldodeca-2',8',10'-trienoyl)-5-methylenefuran-2(*5H*)-one (6)

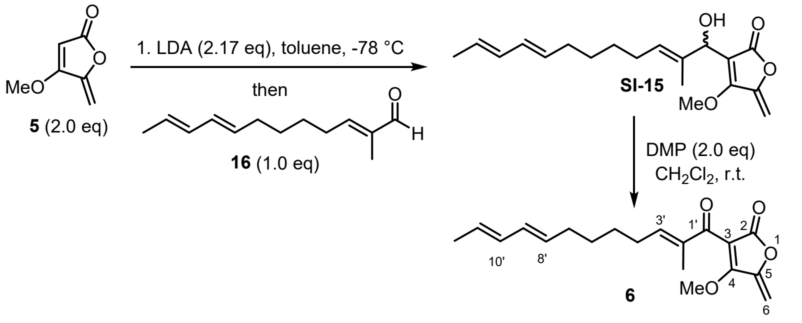

Under an atmosphere of nitrogen, diisopropylamine (0.56 mL, 3.96 mmol, 2.2 eq) was dissolved in toluene (24 mL) and cooled to -78 °C. *n*-BuLi (1.56 mL, 2.5 M in hexanes, 3.91 mmol, 2.17 eq) was added dropwise to the cooled solution over 10 minutes, and the resultant mixture stirred for 1 h. A solution of methyl tetronate **5** (454 mg, 3.60 mmol, 2 eq) in a mixture of toluene (4.20 mL) and THF (3.60 mL) was added dropwise to the LDA solution over 10 minutes. The reaction mixture was stirred for a further 5 minutes, during which a color change was observed from colorless to deep brown. A

solution of aldehyde **4** (346 mg, 1.80 mmol, 1 eq) in toluene (7 mL) was added dropwise to the flask over 15 minutes before the resulting solution was stirred overnight at -78 °C. The reaction was quenched by slow addition of sat. aq. NH_4Cl (20 mL) and the mixture warmed to room temperature. H_2O (10 mL) was added, and the aqueous phase extracted with EtOAc (4 × 15 mL). The combined organic layers were washed with brine, dried over $MgSO_4$, and concentrated *in vacuo* to near dryness before addition of dry CH_2Cl_2 (20 mL). The reaction was purged with N_2 and DMP (1.53 g, 3.61 mmol, 2 eq) was added. The reaction mixture was stirred for a further 2 h at room temperature. Reaction was quenched with sat. aq. $Na_2S_2O_3$ solution (20 mL) and sat. aq. $NaHCO_3$ solution (20 mL) and the aqueous layer extracted with Et_2O (4 × 20 mL). The organic layers were combined, washed with sat. aq. $Na_2S_2O_3$ solution (10 mL) and brine (10 mL) then dried over $MgSO_4$, filtered, and concentrated *in vacuo*. Purification by column chromatography (15% EtOAc:petroleum ether 40 – 60) gave tetronate **6** as a colorless oil (355 mg, 62% yield over 2 steps). **δ_H** (400 MHz, $CDCl_3$) 6.58 (1H, tq, *J* 7.5, 1.5, 3'-H), 6.05-5.93 (2H, m, 9'-H and 10'-H), 5.63-5.46 (2H, m, 8'-H and 11'-H), 5.16 (2H, app. q, *J* 7.0, 6-H_2), 3.91 (3H, s, -OCH_3) 2.31 (2H, app. q, *J* 7.0, 4'-H_2), 2.07 (2H, app. q, *J* 7.0, 7'-H_2), 1.88 (3H, s, 2'-CH_3), 1.73 (3H, d, *J* 6.0, 12'-H_3), 1.51-1.41 (4H, m, 5'-H_2 and 6'-H_2). **δ_C** (100 MHz, $CDCl_3$) 191.2 (C-1'), 166.3 (C-2), 165.5 (C-4), 151.4 (C-3'), 149.0 (C-5), 138.4 (C-2'), 131.6 (C-8'), 131.4 (C-10'), 130.9 (C-9'), 127.3 (C-11'), 104.9 (C-3), 94.3 (C-6), 61.0 (OCH_3), 31.3 (C-7'), 29.6 (C-4'), 29.2 and 27.9 (C-5' and C-6'), 18.1 (C-12'), 11.2 (2'-CH_3). **HRMS** (ESI) calc. $[C_{19}H_{24}O_4 + H]^+$ 317.1747, observed 317.1741.

A portion of **6** was purified by $AgNO_3$-impregnated silica flash column chromatography to obtain a pure *E,E*-isomer to use in subsequent analytical scale assays and large scale biotransformation with TedJ. Following methodology described by Li *et al.,*[28] $AgNO_3$ (5.50 g) in H_2O (40 mL) was combined with silica (40 – 63 µm, 50.0 g) and ground in a large pestle and mortar to evenly disperse the $AgNO_3$ solution throughout the silica. The slurry was transferred to a 500 mL round bottom flask wrapped in aluminum foil and dried in an oven at 160 °C for 16 h. The resulting free-flowing near-white powder was then stoppered and stored at room temperature until use. Flash column chromatography was conducted as usual silica columns without need to wrap column in dark paper or aluminum foil. $AgNO_3$-impregnated TLC plates were prepared by soaking TLC plates in a $AgNO_3$ solution (2.00 g $AgNO_3$ in 5.0 mL H_2O) for 1 min before gentle heating. $AgNO_3$-impregnated TLC plates were visualized using phosphomolybdic acid (PMA) stain with gentle heating. **156** was purified by this method (30% EtOAc: petroleum ether 40 – 60).

Diels-Alder reaction giving *trans*-decalin 8

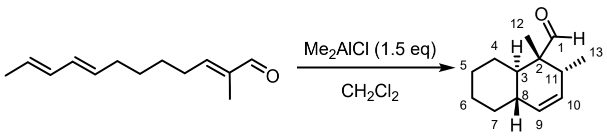

4 8

Under an atmosphere of nitrogen, aldehyde **4** (612 mg, 3.18 mmol, 1 eq) was dissolved in CH_2Cl_2 (63.7 mL) and cooled to -78 °C. A solution of Me_2AlCl (5.30 mL, 0.9 M in heptane, 4.77 mmol, 1.5 eq) was added dropwise over 15 minutes and then the solution stirred for a further 15 minutes. The reaction was warmed to -30 °C and stirred for a further 24 h. Sat. aq. $NaHCO_3$ (15 mL) and sat. aq. $Na_2S_2O_3$ (15 mL) were added and the reaction mixture warmed to room temperature. Additional H_2O (10 mL) was added and the organic layer was separated. The aqueous layer was extracted with Et_2O (4 × 20 mL), the combined organic layers washed sequentially with sat. aq. $NaHCO_3$ (10 mL) and brine (10 mL) then dried over $MgSO_4$, filtered, and concentrated *in vacuo*. Purification by column chromatography (2% Et_2O:petroleum ether 40 – 60) gave racemic *trans*-decalin **8** as a colourless oil (409 mg, 67% yield). $\boldsymbol{\delta_H}$ (400 MHz, d_8-toluene) 9.35 (1H, s, 1-H), 5.28 (1H, m, 10-H), 5.23 (1H, m, 9-H), 1.72 (1H, m, 11-H), 1.60 (3H, m, 5-*H*H, 6-*H*H, 7-*H*H) 1.51 (1H, m, 3-H), 1.45 (1H, m, 8-H), 1.37 (1H, m, 4-H*H*), 1.14 (2H, m, 5-H*H* and 6-H*H*), 1.00 (1H, m, 7-H*H*), 0.95 (3H, s, 12-H_3), 0.80 (1H, m, 4-*H*H), 0.82 (3H, d, *J* 7.0, 13-H_3). $\boldsymbol{\delta_C}$ (100 MHz, d_8-toluene) 206.7 (C-1), 130.3 and 130.2 (C-9 and C-10), 49.5 (C-2), 39.8 (C-11), 39.0 (C-3), 38.2 (C-8), 33.6 (C-7), 27.3 (C-4), 27.1 and 27.0 (C-5 and C-6), 17.3 (C-13), 14.7 (12-C). **IR** (ν_{max}/cm^{-1}) (neat) 2922, 2853, 1703, 1692. **HRMS** (EI) calc. $[C_{13}H_{20}O]^+$ 192.1509, observed 192.1509.

Data previously reported in the literature, but the solvent used for NMR was $CDCl_3$.[23] We found that signals were better resolved using d_8-toluene as the solvent.

Racemic *trans*-decalin tetronate 7

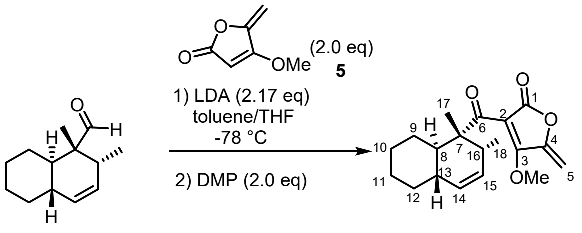

8 7

Under an atmosphere of nitrogen, diisopropylamine (0.74 mL, 5.24 mmol, 2.2 eq) was dissolved in toluene (31.8 mL) was cooled to -78 °C. *n*-BuLi (2.07 mL, 2.5 M in hexanes, 5.18 mmol, 2.17 eq) was added dropwise to the cooled solution over 10 minutes and the resultant mixture stirred for 1 h. A solution of methyl tetronate **5** (454 mg, 4.77 mmol, 2 eq) in a mixture of toluene (5.60 mL) and THF

(4.80 mL) was added dropwise to the LDA solution over 10 minutes. The reaction mixture was stirred for a further 5 minutes, during which a color change was observed from colorless to deep brown. A solution of aldehyde **8** (606 mg, 2.38 mmol, 1 eq) in toluene (9.27 mL) was added dropwise over 15 minutes before the resulting solution was stirred overnight at -78 °C. The reaction was quenched by slow addition of sat. aq. NH_4Cl (25 mL) and the mixture warmed to room temperature. H_2O (10 mL) was added, and the aqueous layer extracted with EtOAc (4 × 20 mL). The combined organic layers were washed with brine, dried over $MgSO_4$, and concentrated *in vacuo* to near dryness before addition of dry CH_2Cl_2 (20 mL). The mixture was purged with N_2 and DMP (2.02 g, 4.77 mmol, 2 eq) added. The reaction mixture was allowed to stir for a further 2 hours prior to reaction completion. Sat. aq. $Na_2S_2O_3$ solution (20 mL) and sat. aq. $NaHCO_3$ solution (20 mL) were added to the mixture and the aqueous layer extracted with Et_2O (4 × 25 mL). The combined organic phases were washed sequentially with sat. aq. $Na_2S_2O_3$ solution (15 mL) and brine (15 mL), then dried over $MgSO_4$, filtered, and concentrated *in vacuo*. Purification by column chromatography (15% Et_2O:petroleum ether 40 – 60) gave *trans*-decalin tetronate **7** as a colorless oil (438 mg, 64% yield over 2 steps). **δ_H** (500 MHz, d_8-toluene) 5.50 (1H, m, 15-H), 5.26 (1H, m, 14-H), 4.70 (1H, d, *J* 2.5, 5-H*H*), 4.62 (1H, d, *J* 2.5, 5-*H*H), 3.37 (3H, s, OCH_3), 3.33 (1H, m, 16-H), 1.91 (1H, td, *J* 11.0, 2.5, 8-H), 1.71-1.62 (3H, m, 10-H*H*, 11-H*H*, 12-H*H*), 1.53 (1H, m, *J* 11.0, 2.5, 13-H), 1.44 (1H, m, 9-H*H*), 1.36 (1H, m, 10-*H*H), 1.21-1.04 (2H, m, 11-*H*H and 12-*H*H), 1.19 (3H, s, 17-H_3), 1.02 (3H, d, *J* 7.0, 18-H_3), 0.70 (1H, app. qd, *J* 12.0, 3.5, 9-*H*H). **δ_C** (126 MHz, d_8-toluene) 202.6 (C-6), 166.9 (C-3), 165.1 (C-1), 149.9 (C-4), 130.9 (C-15), 129.1 (C-14), 108.8 (C-2), 93.1 (C-5), 62.3 (OCH_3), 54.3 (C-7), 40.3 (C-8), 38.9 (C-13), 38.1 (C-16), 34.1 (C-12), 27.5 (C-11), 27.2 (C-10), 27.1 (C-9), 19.3 (C-18), 17.2 (C-17). **IR** (ν_{max}/cm^{-1}) (neat) 2924. 2852, 1770, 1661, 1587. **HRMS** (ESI) calc. $[C_{19}H_{24}O_4 + H]^+$ 317.1753, observed 317.1743.

Epoxide 12

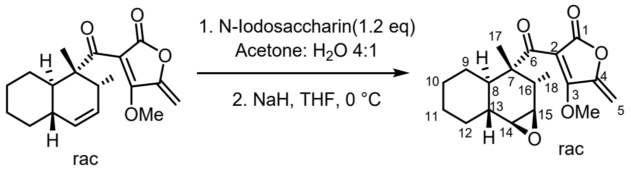

7 **12**

Racemic *trans*-decalin tetronate **7** (40 mg, 0.13 mmol, 1 eq) was dissolved in acetone (20.2 mL) and H_2O (5.1 mL) at room temperature. *N*-iodosaccharin (47.0 mg, 0.155 mmol, 1.2 eq) added with stirring. After 30 min, sat. aq. $NaHCO_3$ (3 mL) and sat. aq. $Na_2S_2O_3$ (3 mL) were added. The mixture was extracted with Et_2O (4 × 5 mL), then the combined organic phases washed with brine (4 mL) and dried over $MgSO_4$, filtered through a silica plug (100% Et_2O), and concentrated *in vacuo*. Crude iodohydrin was redissolved in dry THF (25 mL) and the solution cooled to 0 °C under an atmosphere of N_2. NaH

(33.0 mg, 0.137 mmol, 1.1 eq) was added and the reaction stirred for 1 h at room temperature. Sat. aq. NH_4Cl solution (5 mL) was added and the mixture extracted with EtOAc (3 × 10 mL). The organic layers combined, washed with brine (10 mL), dried over $MgSO_4$ and concentrated *in vacuo*. Purification by column chromatography (10% EtOAc:petroleum ether 40 – 60) gave racemic epoxide **12** as a white solid (27.0 mg, 64% yield). **δ_H** (500 MHz, d_8-toluene) 4.68 (1H, d, *J* 2.5, 5-H*H*), 4.60 (1H, d, *J* 2.5, 5-*H*H), 3.56 (1H, q, *J* 7.5, 16-H), 3.31 (3H, s, OCH_3), 2.77 (1H, d, *J* 4.0, 15-H), 2.51 (1H, d, *J* 4.0, 14-H), 1.72 (1H, app. td, *J* 11.0, 2.0, 8-H), 1.69-1.59 (3H, m, 10-H*H*, 11-H*H*, 12-H*H*), 1.58 (1H, m, 13-H), 1.40 (3H, s, 17-H_3), 1.30 (1H, m, 9-H*H*), 1.21-1.05 (3H, m, 10-*H*H, 11-*H*H and 12-*H*H), 0.92 (3H, d, *J* 7.5, 18-H_3), 0.60 (1H, m, 9-*H*H). **δ_C** (126 MHz, d_8-toluene) 202.4 (C-6), 167.3 (C-3), 164.9 (C-1), 149.8 (C-4), 108.6 (C-2), 93.2 (C-5), 62.1 (OCH_3), 59.0 (C-15), 55.3 (C-14), 54.1 (C-7), 39.8 (C-8), 37.5 (C-13), 33.8 (C-16), 33.0 (C-12), 28.2 (C-9), 27.3 and 27.2 (C-10 and C-11), 18.3 (C-17), 15.4 (C-18). **IR** (ν_{max}/cm^{-1}) (neat) 2923, 2853, 1770, 1663, 1586. **HRMS** (ESI) calc. $[C_{19}H_{24}O_5 + H]^+$ 333.1702, observed 333.1700.

The above reaction was repeated using enantiopure *trans*-decalin **(-)-7** (8.00 mg) from the bioassays with TedJ giving (−)-epoxide **12** (6.00 mg, 75% yield) $[\alpha]_D^{25}$ -17 (*c* 1.5, $CHCl_3$). The spectral data were the same as above.

Treatment of racemic *trans*-decalin 7 with *m*-CPBA to give racemic epoxide 9

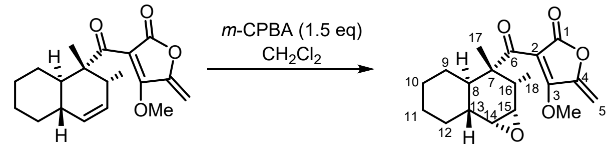

7 **9**

Trans-decalin **7** (40 mg, 0.13 mmol, 1 eq) was dissolved in CH_2Cl_2 (10 mL) at room temperature, and *m*-CPBA (33.0 mg, 0.191 mmol, 1.5 eq) was added portionwise with stirring. After 8 h, sat. aq. $NaHCO_3$ (10 mL) was added to the reaction mixture and the layers separated. The aqueous phase was extracted with further Et_2O (3 × 10 mL), the organic phases combined, washed with sat. aq. $NaHCO_3$ (10 mL), brine, dried over $MgSO_4$ and concentrated *in vacuo*. Purification by column chromatography (20% Et_2O:petroleum ether 40 – 60) gave epoxide **9** as a white solid (36.0 mg, 86% yield). **δ_H** (500 MHz, d_8-toluene) 4.70 (1H, d, *J* 2.5, 5-H*H*), 4.61 (1H, d, *J* 2.5, 5-*H*H), 3.32 (3H, s, OCH_3), 3.10 (1H, m, 16-H), 2.89 (1H, dd, *J* 5.5, 4.0, 15-H), 2.68 (1H, dd, *J* 4.0, 1.5, 14-H), 1.98 (1H, app. td, *J* 11.5, 2.5, 8-H), 1.67-1.62 (2H, m, 12-H*H* and 11- *H*H), 1.58 (1H, m, 10-*H*H), 1.43 (1H, m, 12-*H*H), 1.31 (1H, m, 10-H*H*), 1.26 (1H, m, 9-H*H*), 1.22 (1H, m, 13-H), 1.14 (3H, d, *J* 7.0, 18-H_3), 1.09 (1H, m, 11-H*H*), 1.08 (3H, s, 17-H_3), 0.59 (1H, qd, *J*, 12.0, 3.5, 9-*H*H). **δ_C** (126 MHz, d_8-toluene) 201.4 (C-6), 167.5 (C-3), 165.1 (C-1), 149.9 (C-4), 108.6 (C-2), 93.4 (C-5), 62.5 (OCH_3), 56.6 (C-14), 54.6 (C-15), 53.7 (C-7), 37.3 (C-13), 36.0 (C-8), 35.0 (C-

16), 30.8 (C-12), 27.0 (C-11), 27.0 (C-9), 26.9 (C-10), 17.9 (C-17), 14.7 (C-18). **IR** (ν_{max}/cm^{-1}) (neat) 2923, 2852, 1770, 1662, 1585. **HRMS** (ESI) calc. $[C_{19}H_{24}O_5 + Na]^+$ 355.1521, observed 355.1532.

13-Deoxytetrodecamycin 3

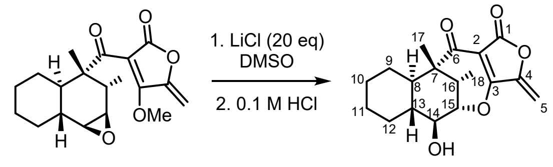

Racemic epoxide **12** (27.0 mg, 81.2 µmol, 1 eq) was dissolved in DMSO (4.00 mL) and LiCl (69.0 mg, 1.63 mmol, 20 eq) added and the solution was heated to 50 °C. After 1 h, the solution was cooled to room temperature and 0.1 M HCl (one drop) was added then the mixture stirred for a further hour. The solvent was removed *via* freeze-drying, the residue dissolved in isopropanol and filtered through a silica plug (in isopropanol) to remove residual LiCl. Purification by reverse-phase HPLC (5 – 95% MeCN:H_2O) followed by removal of solvent *in vacuo* gave racemic 13-deoxytetrodecamycin **3** as a white solid (8.50 mg, 33% yield over 2 steps). δ_H (700 MHz, $CHCl_3$) 5.36 (1H, d, *J* 2.7, 5-H*H*), 5.26 (1H, d, *J* 2.7, 5-H*H*), 4.76 (1H, dd, *J* 2.9, 1.1, 15-H), 3.59 (1H, d, *J* 7.0, 14-H), 2.28 (1H, m, 12-H*H*), 2.12 (1H, qd, *J* 7.4, 2.9, 16-H), 1.73 (1H, m, 11-H*H*), 1.72 (1H, m, 9-H*H*), 1.58 (1H, m, 10-H*H*), 1.22 (1H, m, 8-H), 1.21 (1H, m, 13-H), 1.16 (1H, m, 10-*H*H), 1.14 (1H, m, 9-*H*H), 1.14 (3H, s, 17-H_3), 1.05 (1H, m, 11-*H*H), 1.03 (3H, d, *J* 7.4), 0.96 (1H, m, 12-*H*H). δ_C (176 MHz, $CHCl_3$) 195.0 (C-6), 165.0 (C-1), 164.6 (C-3), 148.6 (C-4), 101.3 (C-2), 96.5 (C-5), 92.1 (C-15), 79.9 (C-14), 53.6 (C-7), 40.9 (C-8), 36.8 (C-13), 34.4 (C-16), 32.7 (C-12), 27.3 (C-10), 25.9 (C-11), 25.1 (C-9), 16.7 (C-17), 13.7 (C-18). **IR** (ν_{max}/cm^{-1}) (neat) 3401, 2921, 2852, 1781, 1670, 1589. **HRMS** (ESI) calc. $[C_{18}H_{22}O_5 + Na]^+$ 341.1365, observed 341.1360.

The above was repeated using (–)-epoxide **12** (5.00 mg) prepared by the chemoenzymatic approach**,** giving (–)-13-deoxytetrodecamycin **3** (2.00 mg) $[\alpha]_D^{23}$ -14° (*c* 0.75, $CHCl_3$), literature $[\alpha]_D^{20}$ -17.6° (*c* 0.65, $CHCl_3$).[29]

Table S4. Synthetic 13-deoxytetrodecamycin (**3**), ^{13}C (176 MHz) and ^{1}H NMR (700 MHz) in $CDCl_3$ compared to 13-deoxytetrodecamycin (**3**) derived from *Streptomyces* WAC04657.[29]

**1**	165	165.0		
**2**	101.3	101.3		
**3**	164.6	164.6		
**4**	148.5	148.56		
**5**	96.5	96.5	5.36, d, *J* 2.7<br>5.26, d, *J* 2.7	5.36, d, *J* 2.7<br>5.26, d, *J* 2.7
**6**	195.0	195.0		
**7**	53.6	53.6		
**8**	40.9	40.9	1.23, m	1.22, m
**9**	25.1	25.1	1.72, m<br>1.15, m	1.72, m<br>1.14, m
**10**	27.3	27.3	1.57, m<br>1.17, m	1.58, m<br>1.16, m
**11**	25.9	25.9	1.73, m<br>1.05, m	1.73, m<br>1.06, m
**12**	32.7	32.7	2.30, m<br>0.96 (tdd (13.2, 10.5, 3.6)	2.28, m<br>0.96, m
**13**	36.8	36.8	1.21, m	1.21, m
**14**	79.8	79.87	3.59 (1H, d, *J* 4.2)	3.59 (1H, d, *J* 7.0)
**15**	92.1	92.1	4.75 (1H, dd *J* 2.8, 1.8)	4.76 (1H, dd, *J* 2.9, 1.1)
**16**	34.4	34.4	2.13 (1H, dq, *J* 7.4, 2.9)	2.12 (1H, qd, *J* 7.4, 2.9)
**17**	16.7	16.7	1.14, s	1.14, s
**18**	13.7	13.7	1.02 (3H, d, *J* 7.4)	1.03 (3H, d, *J* 7.4)

4. Spectra

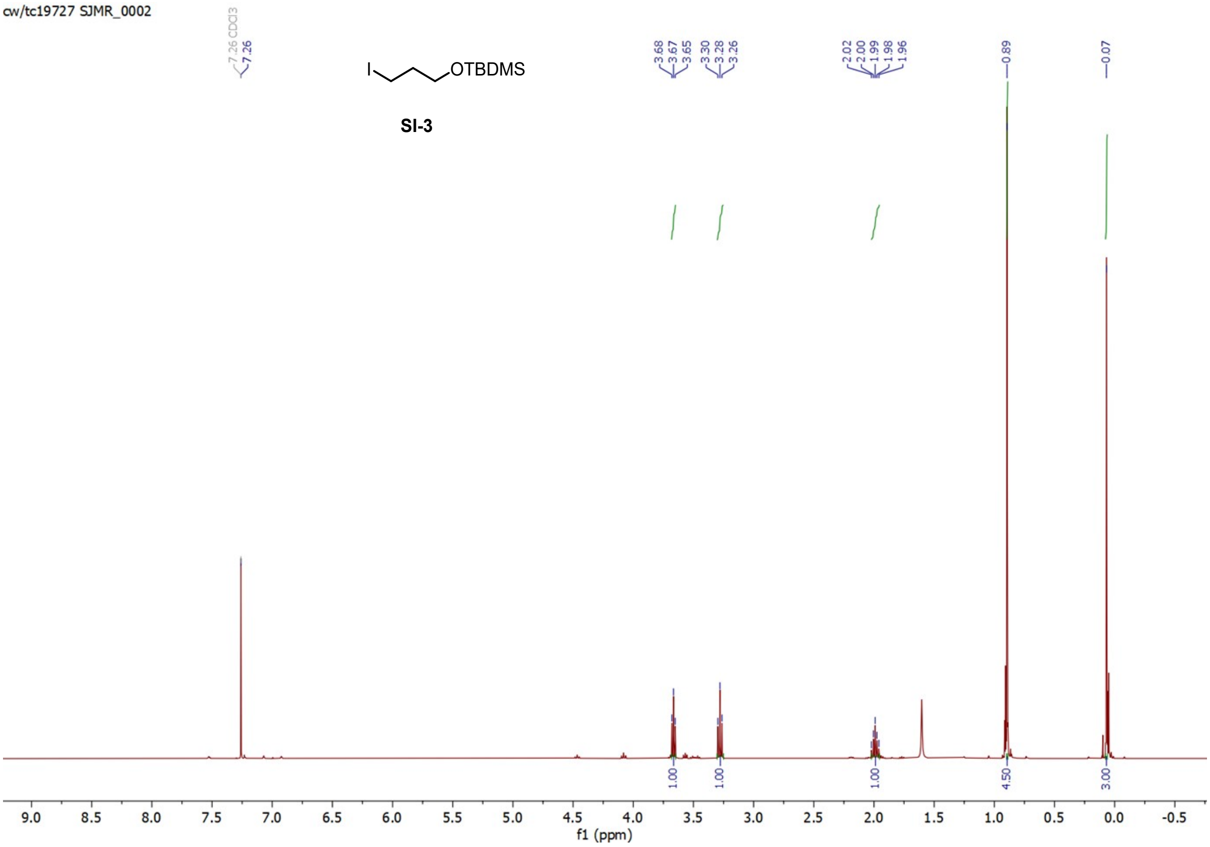
cw/tc19727 SJMR_0002
7.26 CDCl3
7.26
I OTBDMS
SI-3
3.68
3.67
3.65
3.30
3.28
3.26
2.02
2.00
1.99
1.98
1.96
0.89
0.07
1.00
1.00
1.00
4.50
3.00
f1 (ppm)

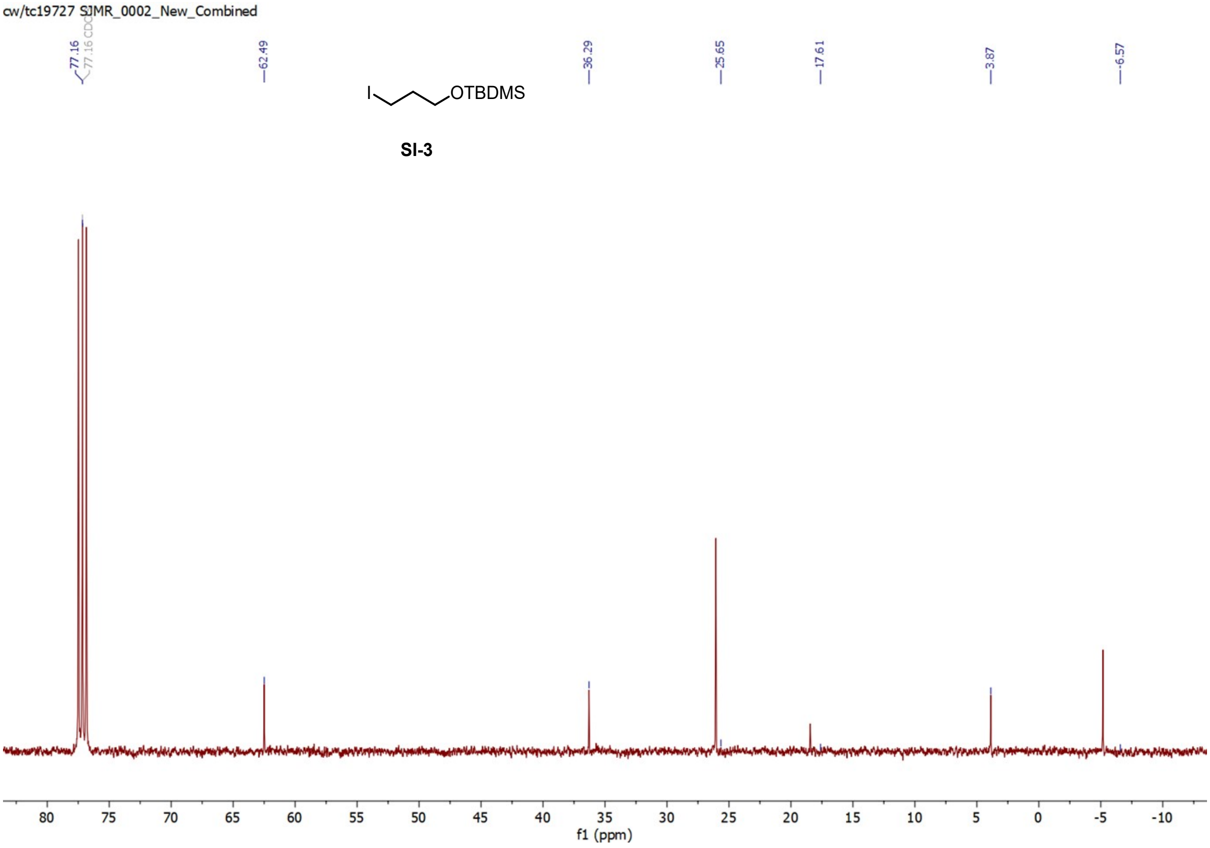
cw/tc19727 SJMR_0002_New_Combined
77.16
77.16 CDCl3
62.49
36.29
25.65
17.61
3.87
-6.57
I OTBDMS
SI-3
f1 (ppm)

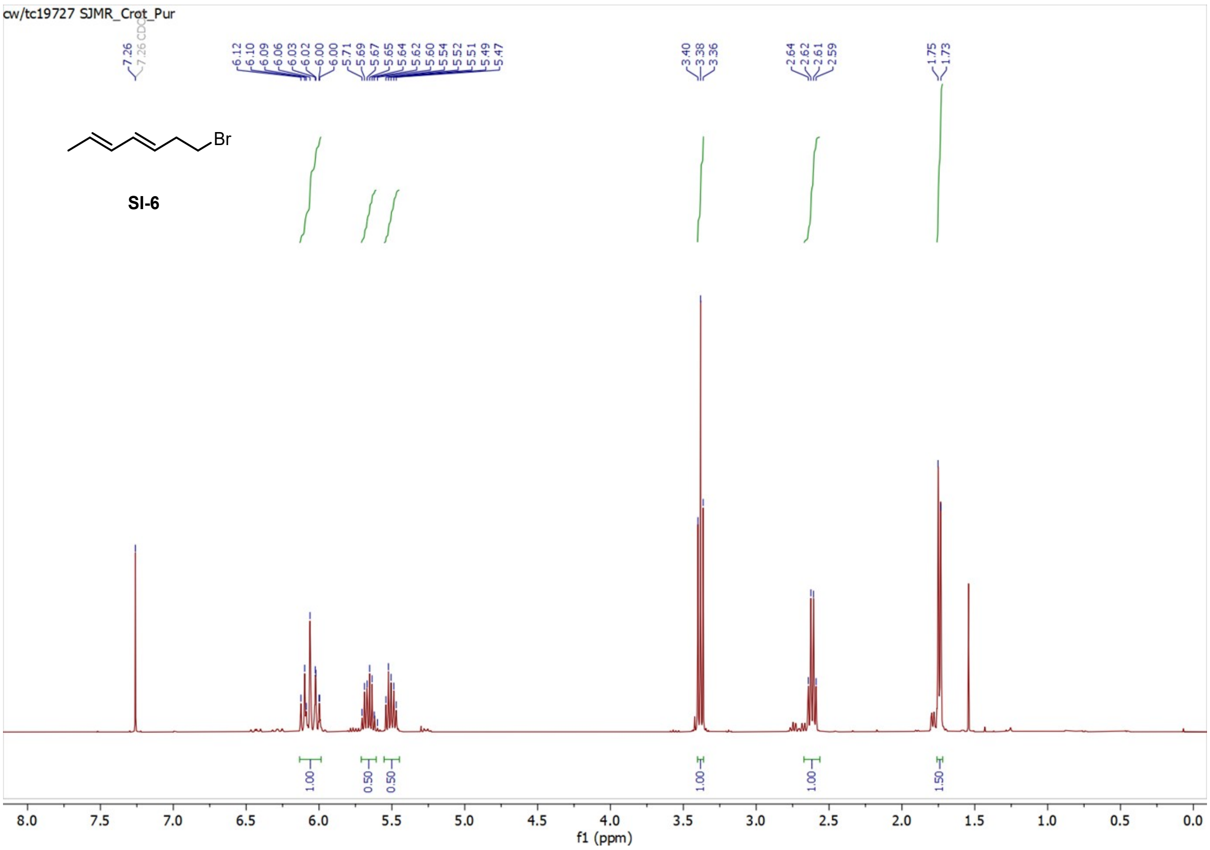
cw/tc19727 SJMR_Crot_Pur
Br
SI-6
f1 (ppm)

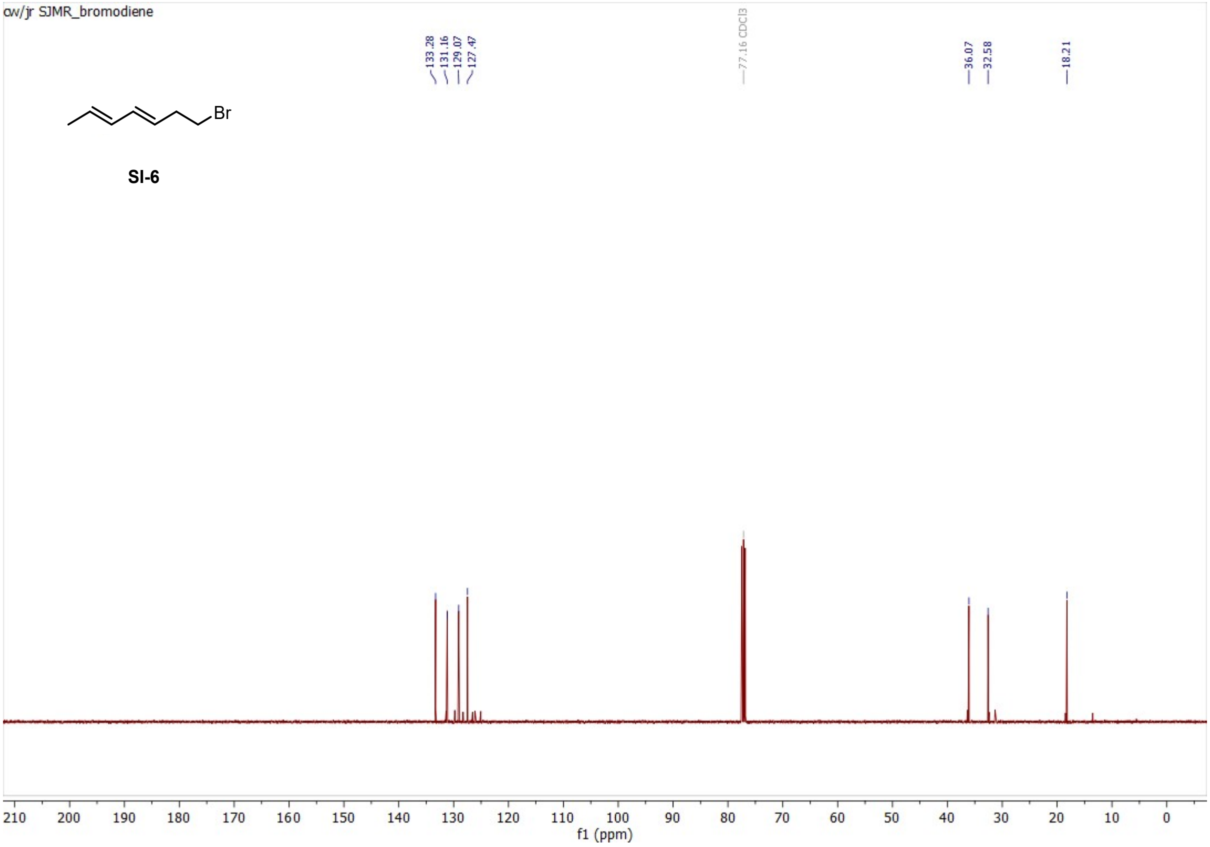
cw/jr SJMR_bromodiene
133.28
131.16
129.07
127.47
77.16 CDCl3
36.07
32.58
18.21
Br
SI-6
f1 (ppm)

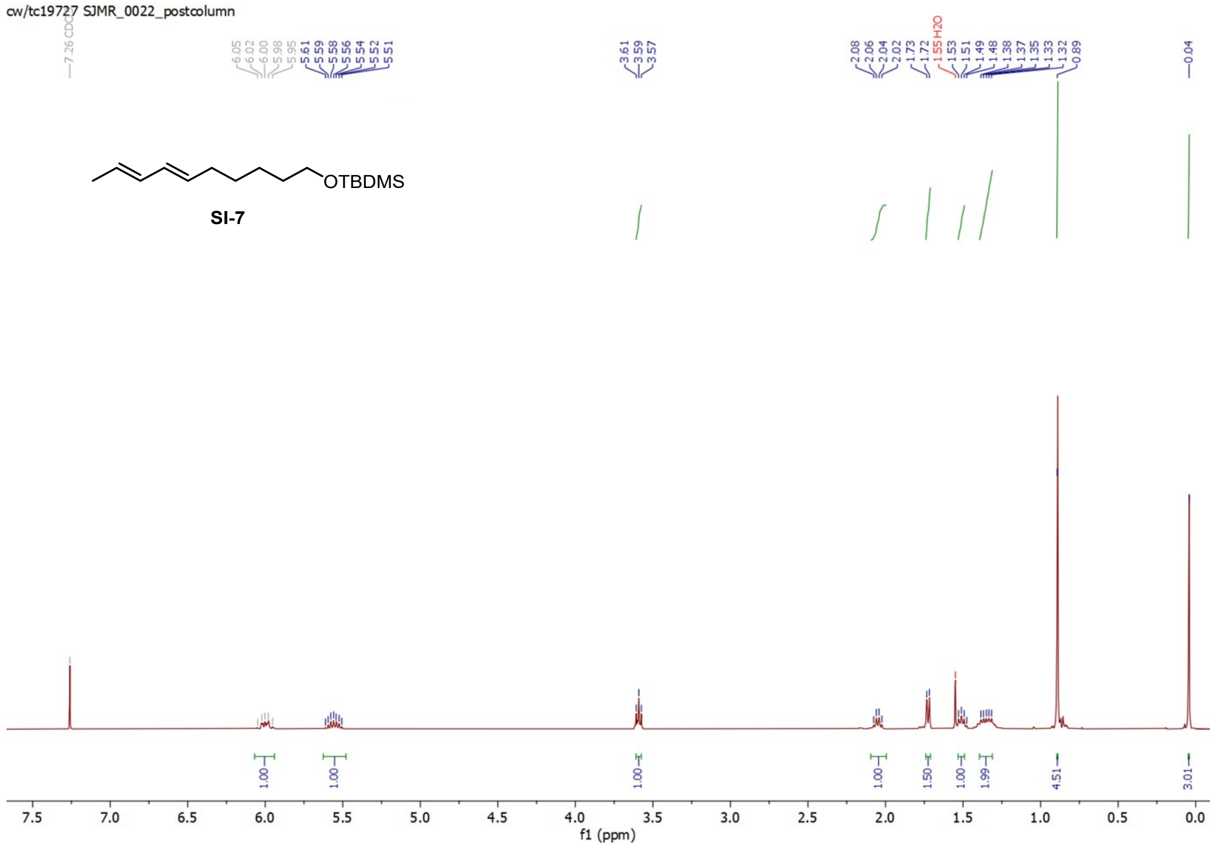
cw/tc19727 SJMR_0022_postcolumn
OTBDMS
SI-7
f1 (ppm)

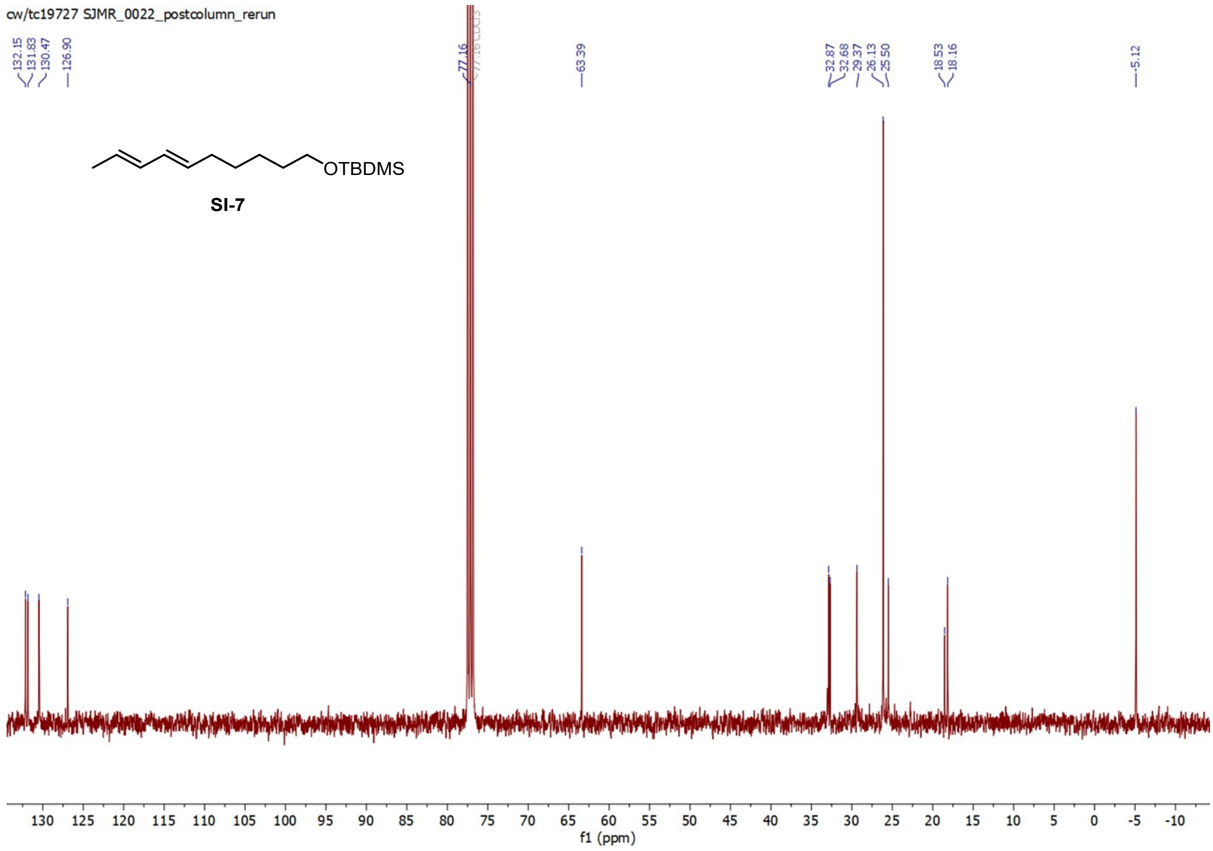
cw/tc19727 SJMR_0022_postcolumn_rerun
OTBDMS
SI-7
f1 (ppm)

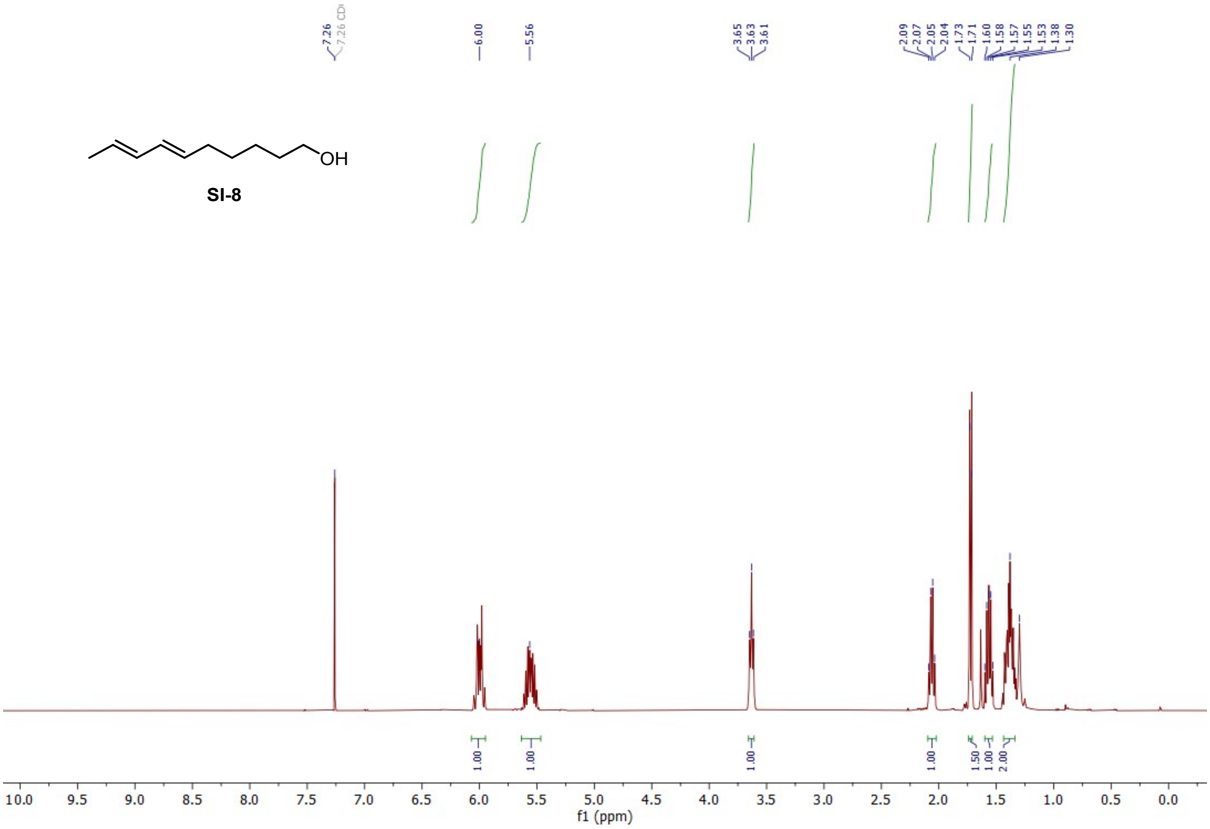

SI-8
OH
7.26
7.26 CDCl3
6.00
5.56
3.65
3.63
3.61
2.09
2.07
2.05
2.04
1.73
1.71
1.60
1.58
1.57
1.55
1.53
1.38
1.30
1.00
1.00
1.00
1.00
1.50
1.00
2.00
f1 (ppm)

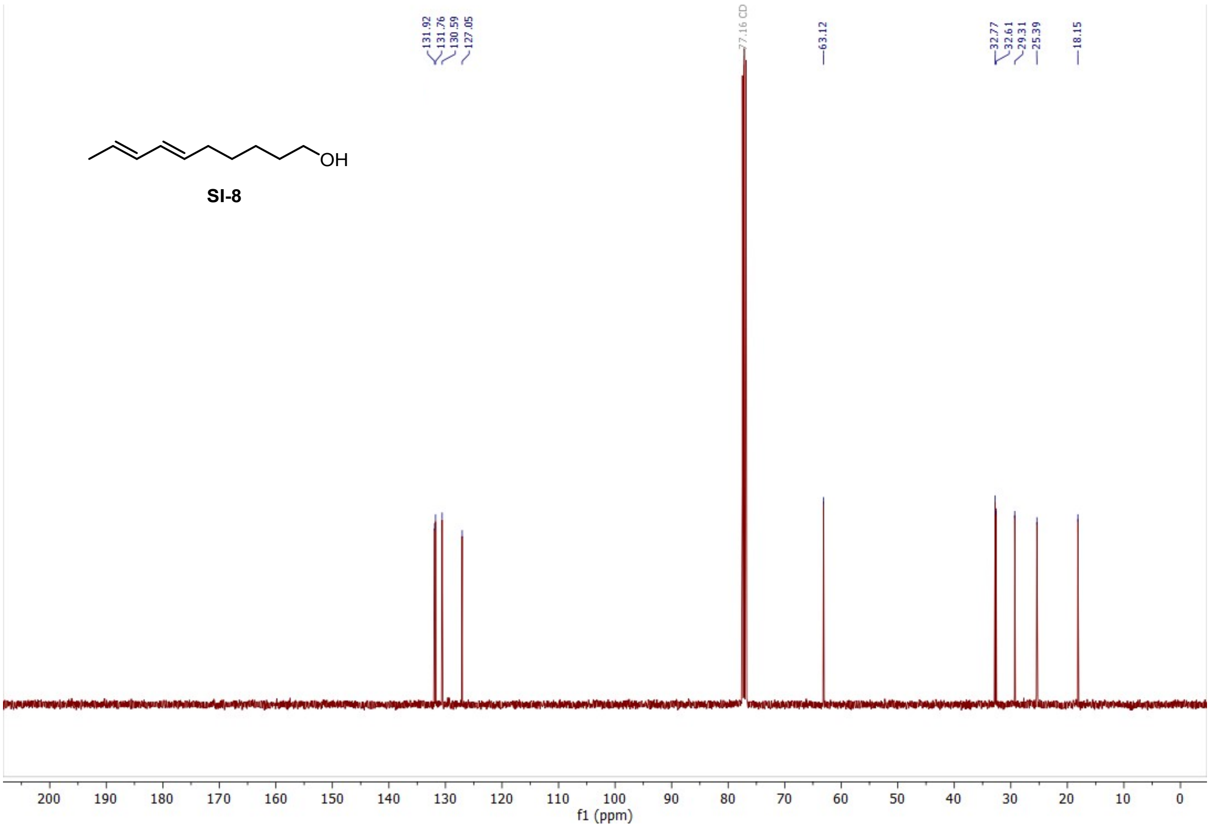

SI-8
OH
131.92
131.76
130.59
127.05
77.16 CD
63.12
32.77
32.61
29.31
25.39
18.15
f1 (ppm)

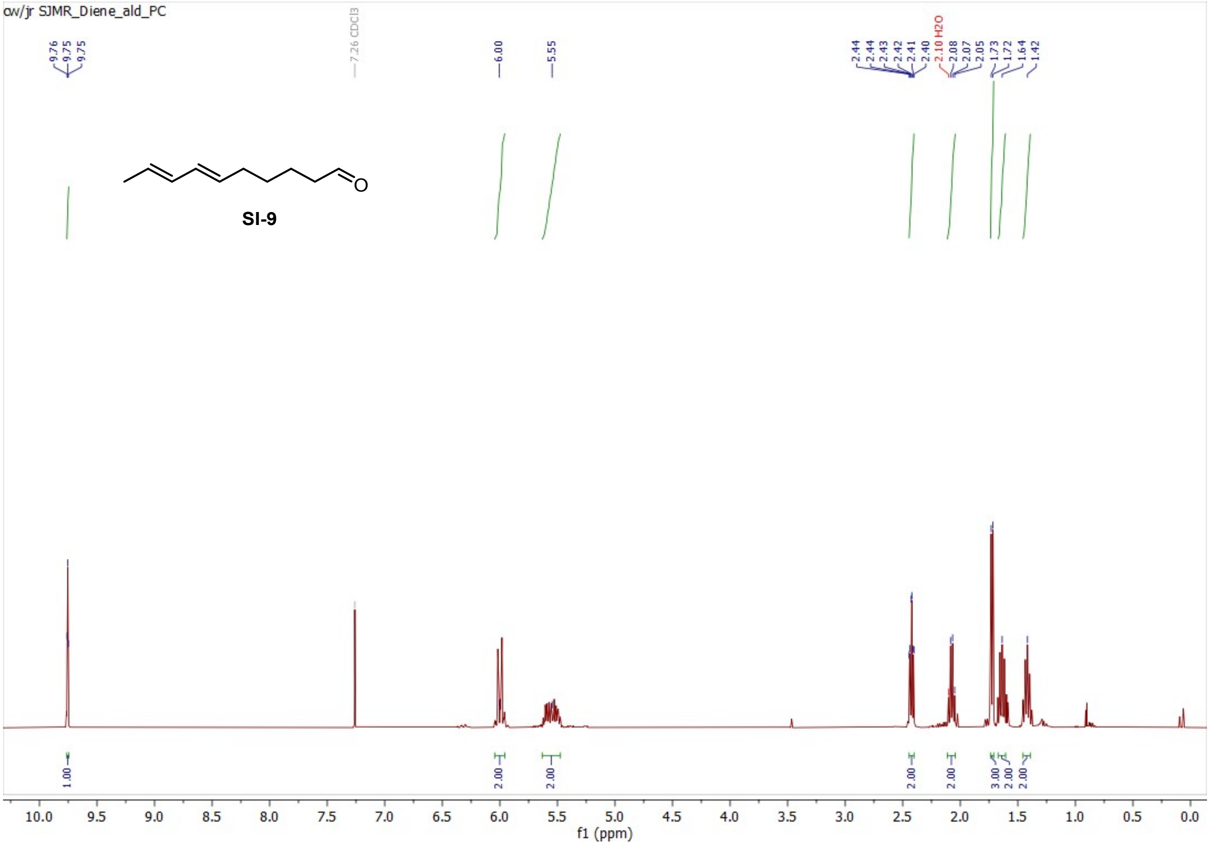
cw/jr SJMR_Diene_ald_PC
9.76
9.75
9.75
7.26 CDCl3
6.00
5.55
2.44
2.44
2.43
2.42
2.41
2.40
2.10 H2O
2.08
2.07
2.05
1.73
1.72
1.64
1.42
SI-9
1.00
2.00
2.00
2.00
2.00
3.00
2.00
2.00
10.0 9.5 9.0 8.5 8.0 7.5 7.0 6.5 6.0 5.5 5.0 4.5 4.0 3.5 3.0 2.5 2.0 1.5 1.0 0.5 0.0
f1 (ppm)

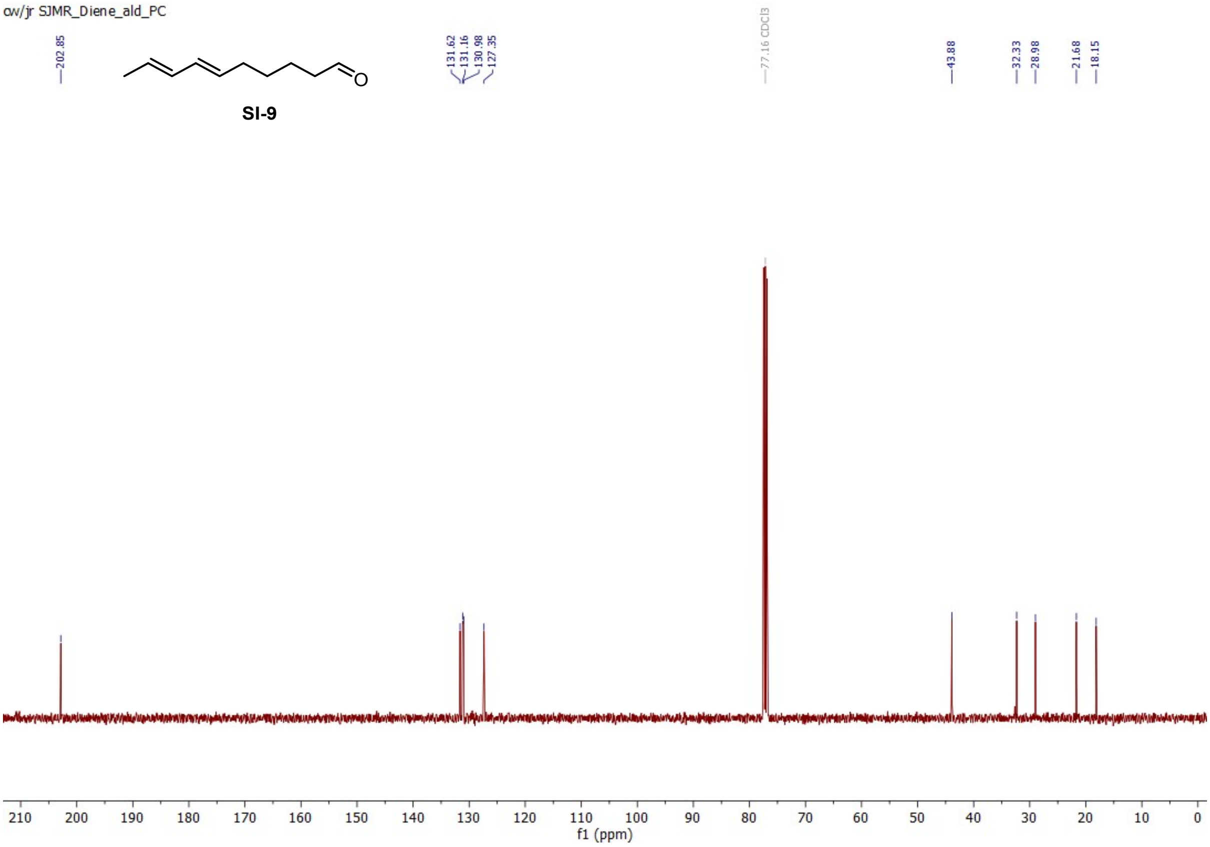
cw/jr SJMR_Diene_ald_PC
202.85
131.62
131.16
130.98
127.35
77.16 CDCl3
43.88
32.33
28.98
21.68
18.15
SI-9
210 200 190 180 170 160 150 140 130 120 110 100 90 80 70 60 50 40 30 20 10 0
f1 (ppm)

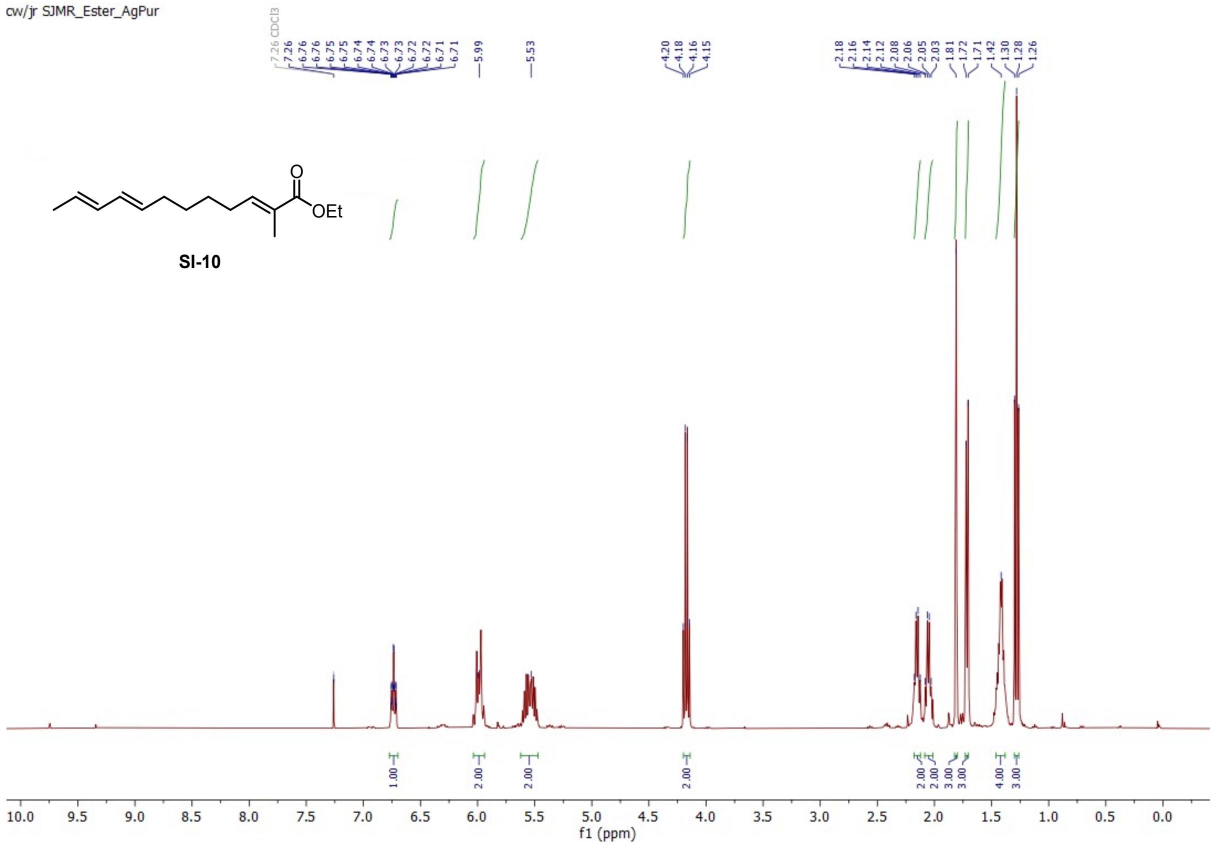

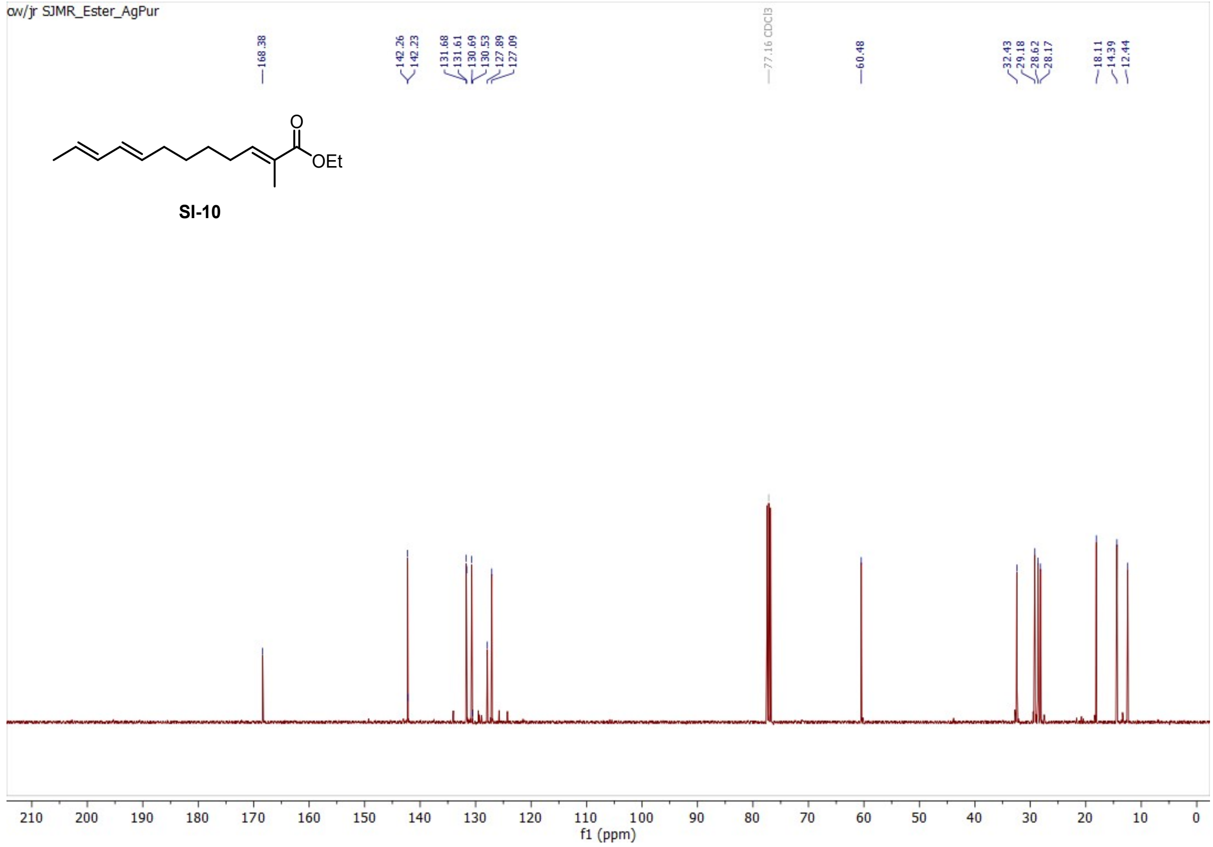

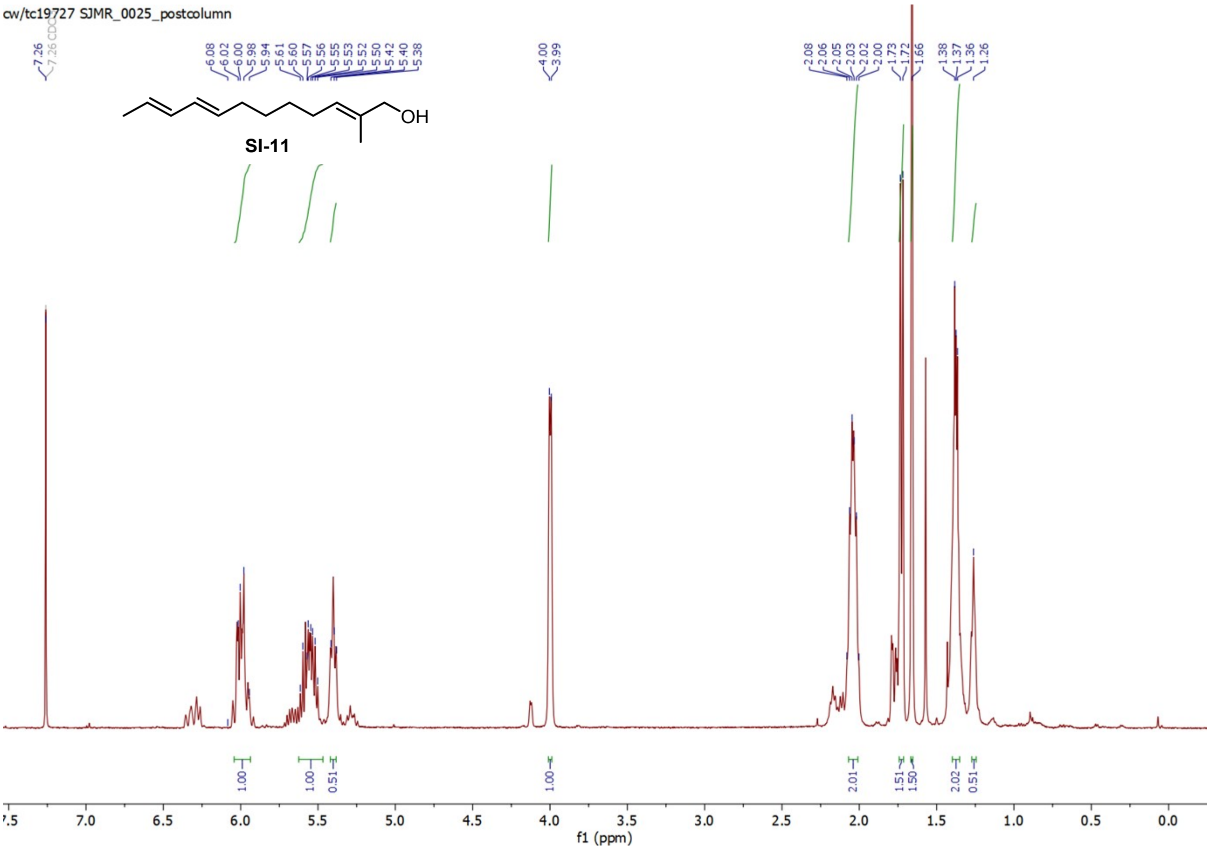
cw/tc19727 SJMR_0025_postcolumn
7.26
7.26 CDCl3
6.08
6.02
6.00
5.98
5.94
5.61
5.60
5.57
5.56
5.55
5.53
5.52
5.50
5.42
5.40
5.38
4.00
3.99
2.08
2.06
2.05
2.03
2.02
2.00
1.73
1.72
1.66
1.38
1.37
1.36
1.26
OH
SI-11
1.00
1.00
0.51
1.00
2.01
1.51
1.50
2.02
0.51
f1 (ppm)

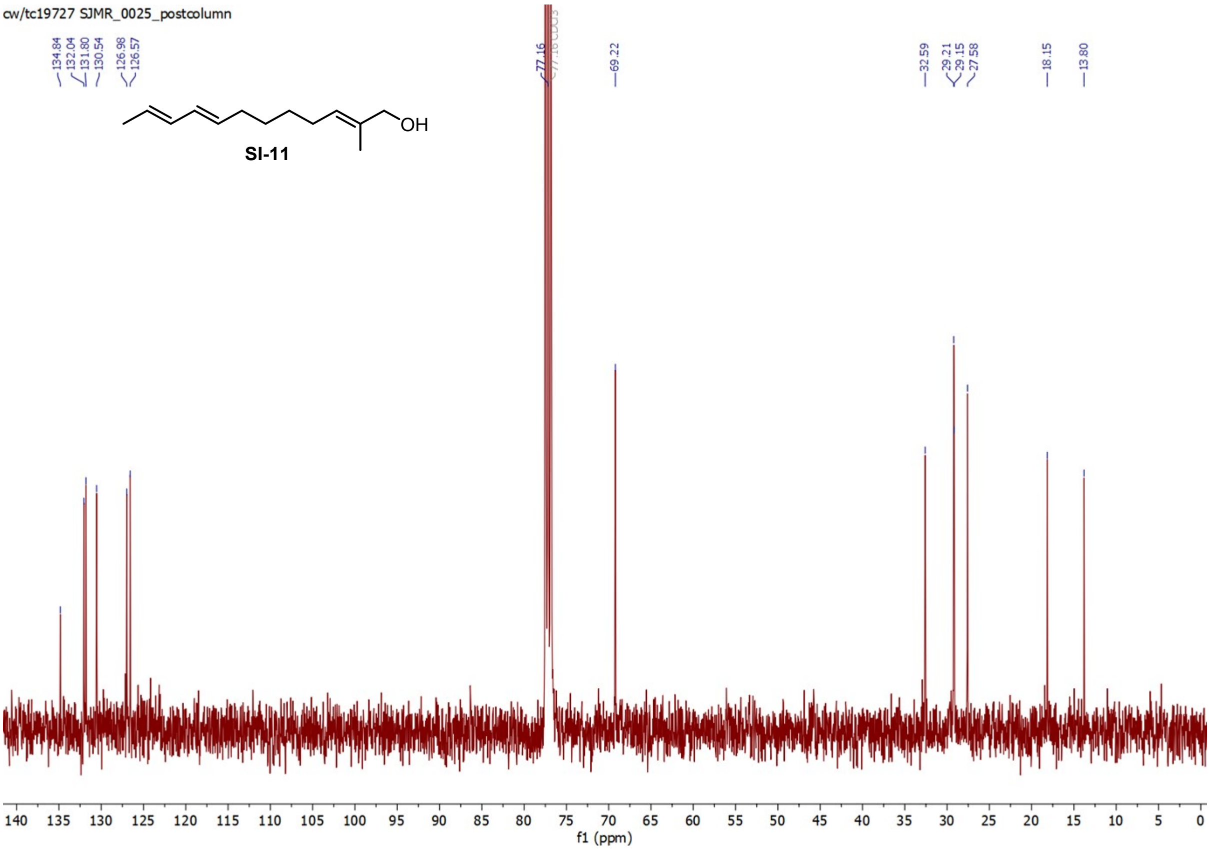
cw/tc19727 SJMR_0025_postcolumn
134.84
132.04
131.80
130.54
126.98
126.57
77.16
77.16 CDCl3
69.22
32.59
29.21
29.15
27.58
18.15
13.80
OH
SI-11
f1 (ppm)

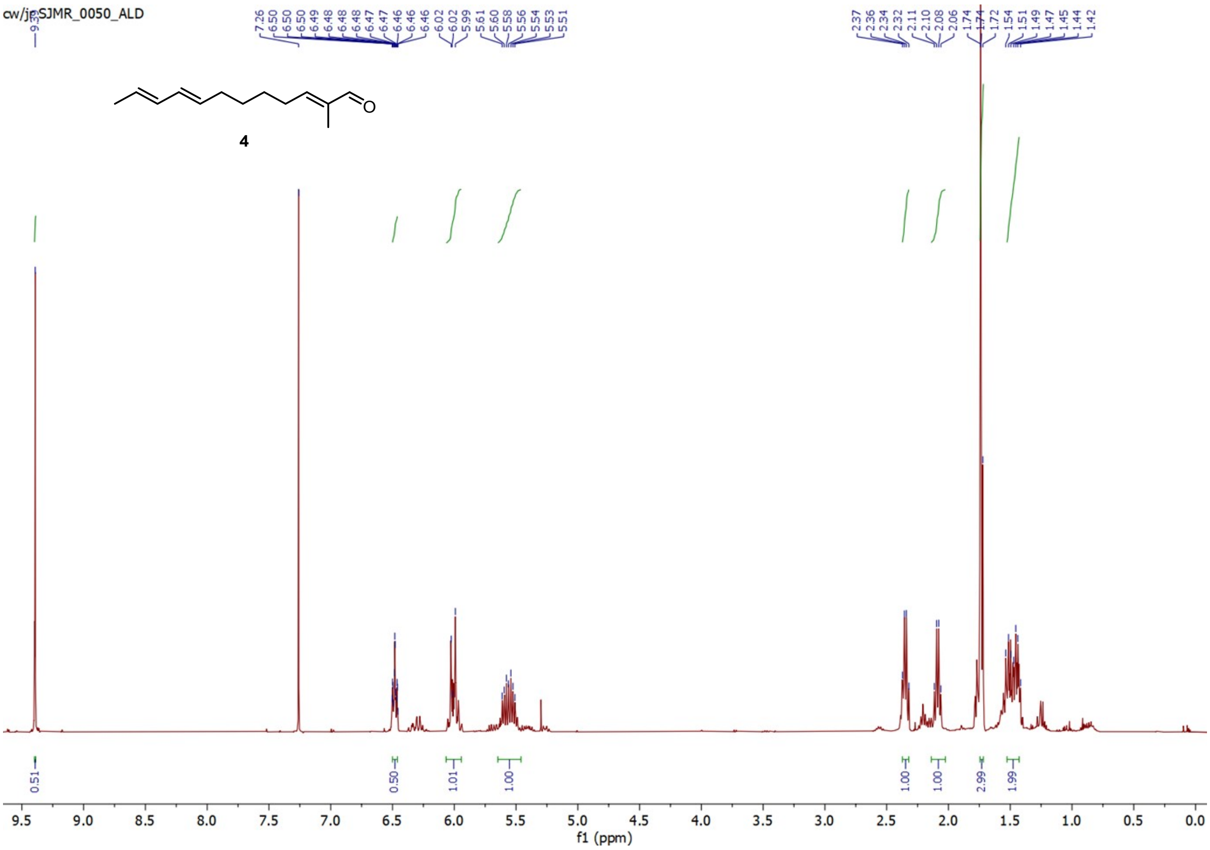
cw/jr SJMR_0050_ALD
4
7.26
6.50
6.50
6.49
6.48
6.48
6.48
6.47
6.46
6.46
6.46
6.02
5.99
5.61
5.60
5.58
5.56
5.54
5.53
5.51
2.37
2.36
2.34
2.32
2.11
2.10
2.08
2.06
1.74
1.74
1.72
1.54
1.51
1.49
1.47
1.45
1.44
1.42
0.51
0.50
1.01
1.00
1.00
1.00
2.99
1.99
9.5 9.0 8.5 8.0 7.5 7.0 6.5 6.0 5.5 5.0 4.5 4.0 3.5 3.0 2.5 2.0 1.5 1.0 0.5 0.0
f1 (ppm)

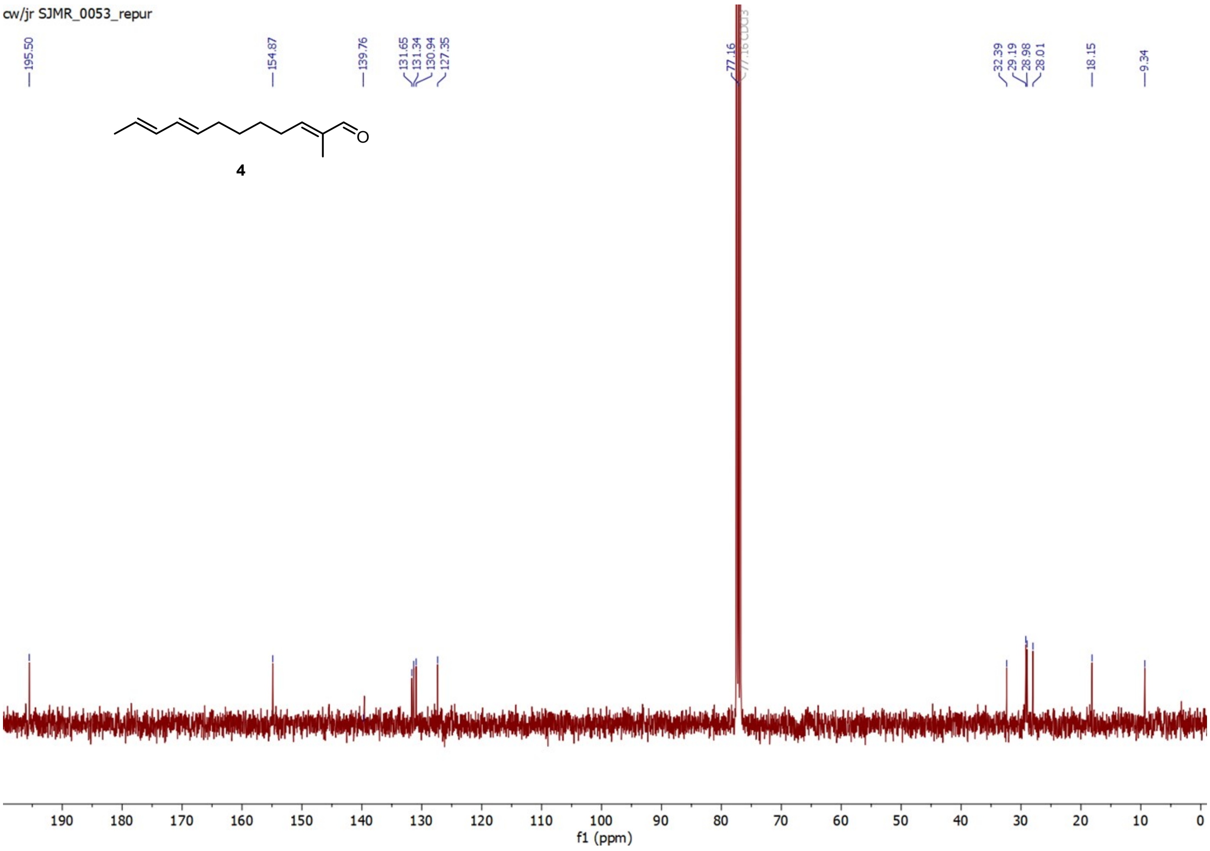
cw/jr SJMR_0053_repur
195.50
154.87
139.76
131.65
131.34
130.94
127.35
77.16
32.39
29.19
28.98
28.01
18.15
9.34
4
190 180 170 160 150 140 130 120 110 100 90 80 70 60 50 40 30 20 10 0
f1 (ppm)

cw/tc19727 SJMR_0028_conc

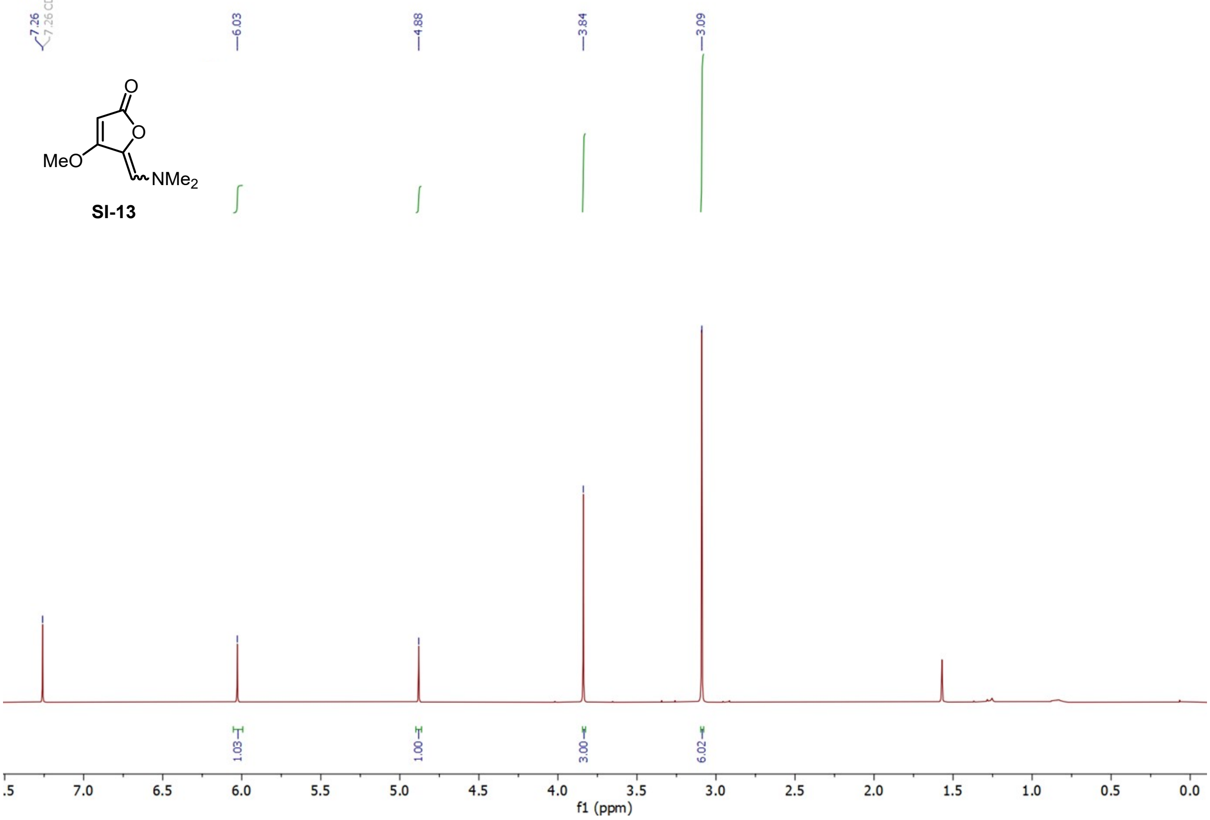

cw/tc19727 SJMR_0028_postcolumn

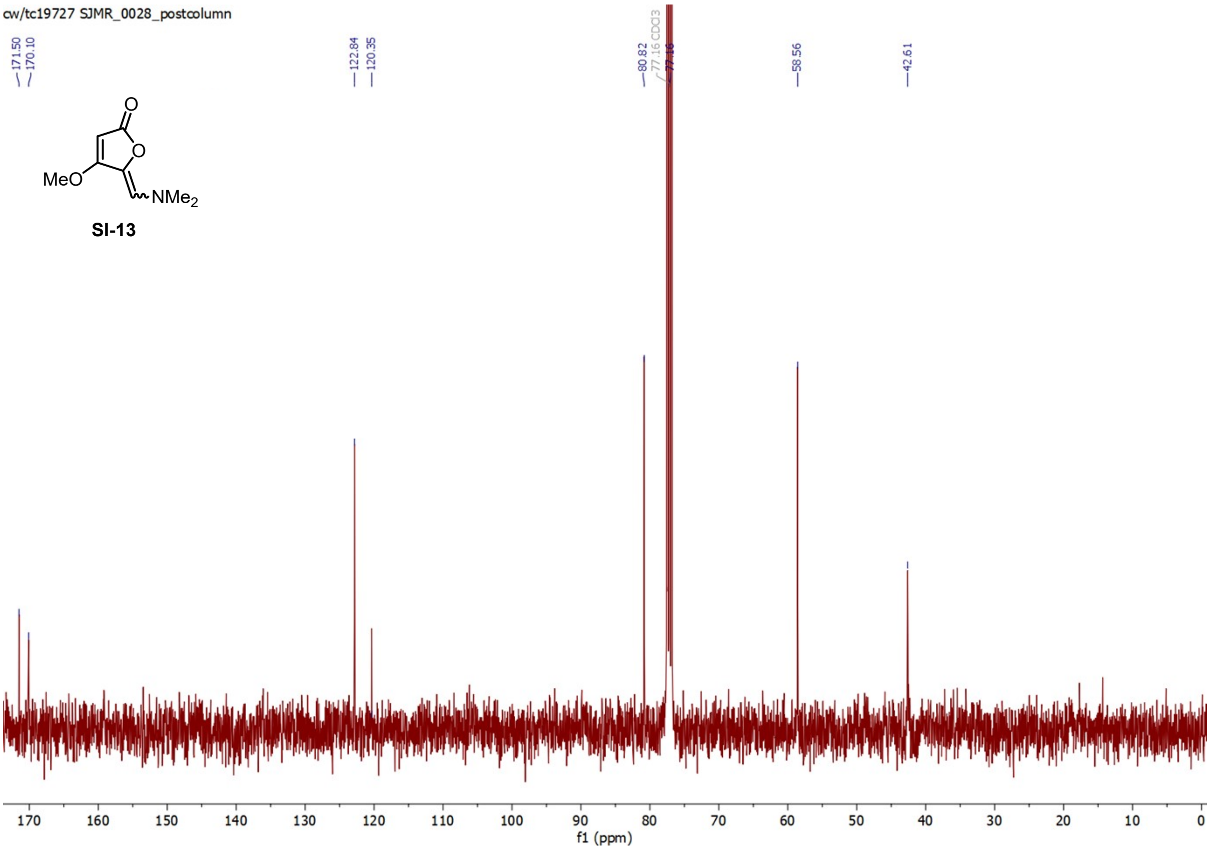

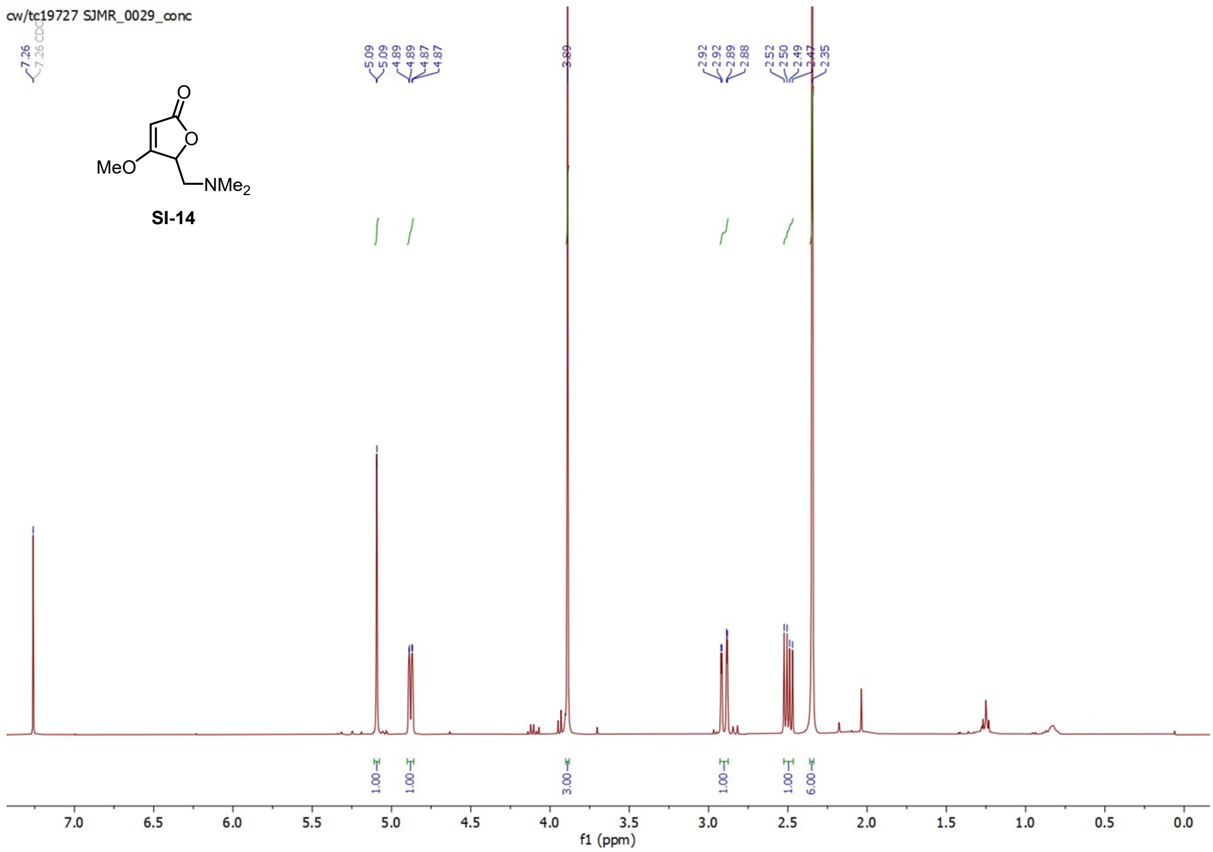
cw/tc19727 SJMR_0029_conc
SI-14
f1 (ppm)

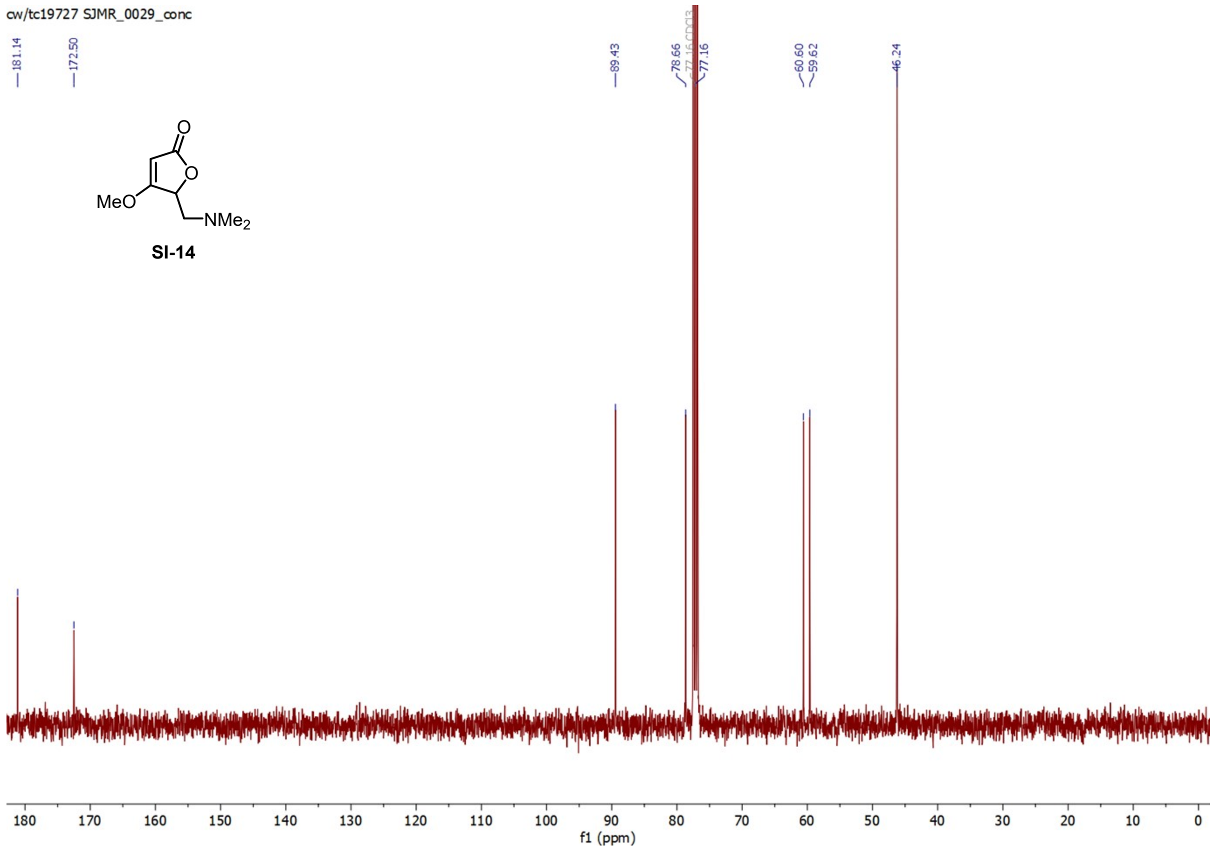
cw/tc19727 SJMR_0029_conc
SI-14
f1 (ppm)

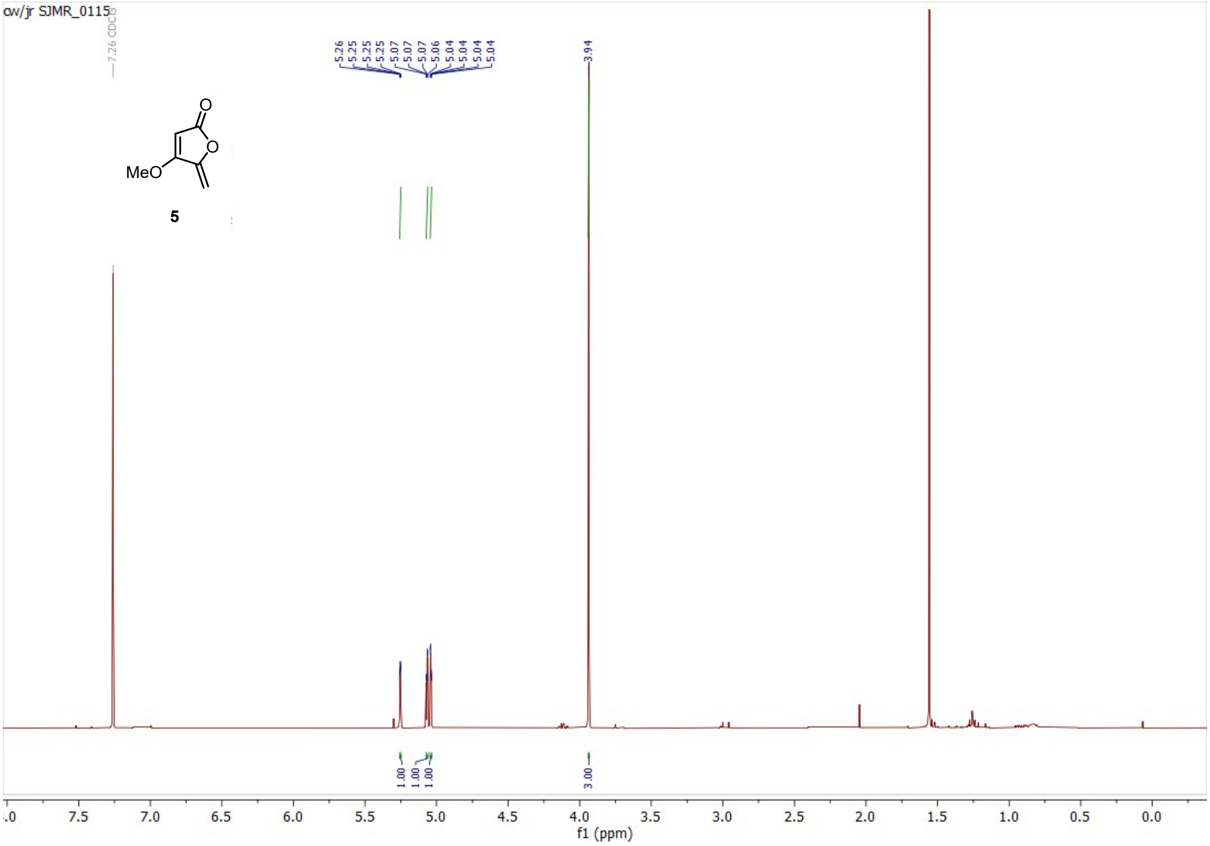
cw/jr SJMR_0115
7.26 CDCl3
5.26
5.25
5.25
5.25
5.07
5.07
5.07
5.06
5.04
5.04
5.04
5.04
3.94
MeO
O
O
5
1.00
1.00
1.00
3.00
f1 (ppm)

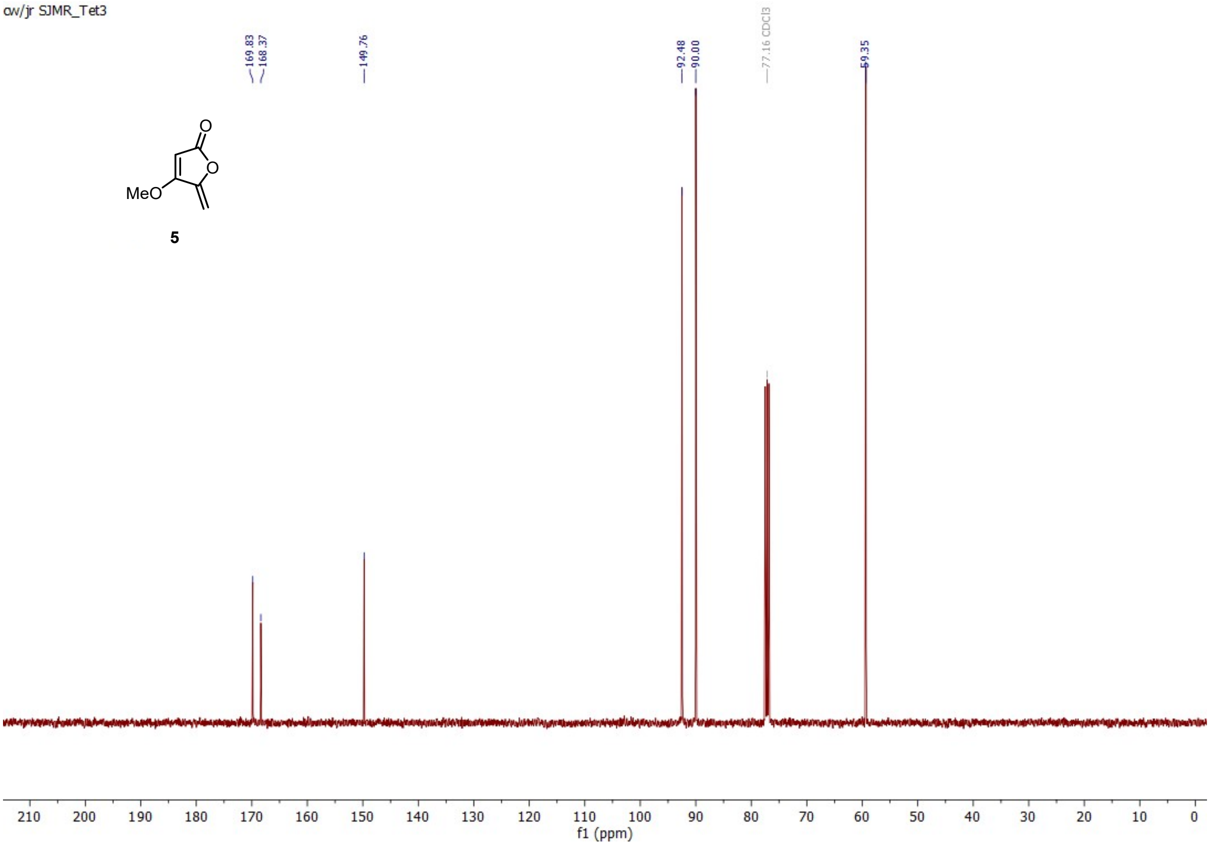
cw/jr SJMR_Tet3
169.83
168.37
149.76
92.48
90.00
77.16 CDCl3
59.35
MeO
O
O
5
f1 (ppm)

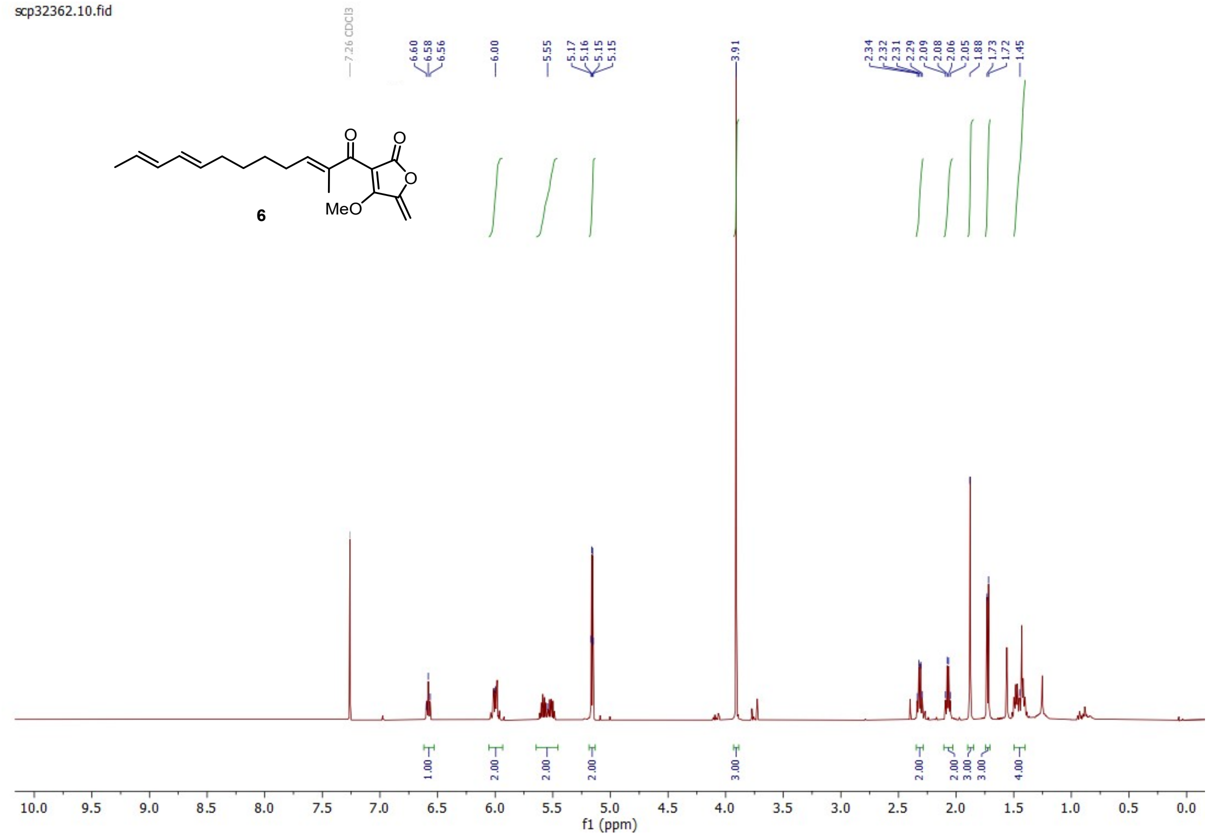
scp32362.10.fid
6
MeO
f1 (ppm)

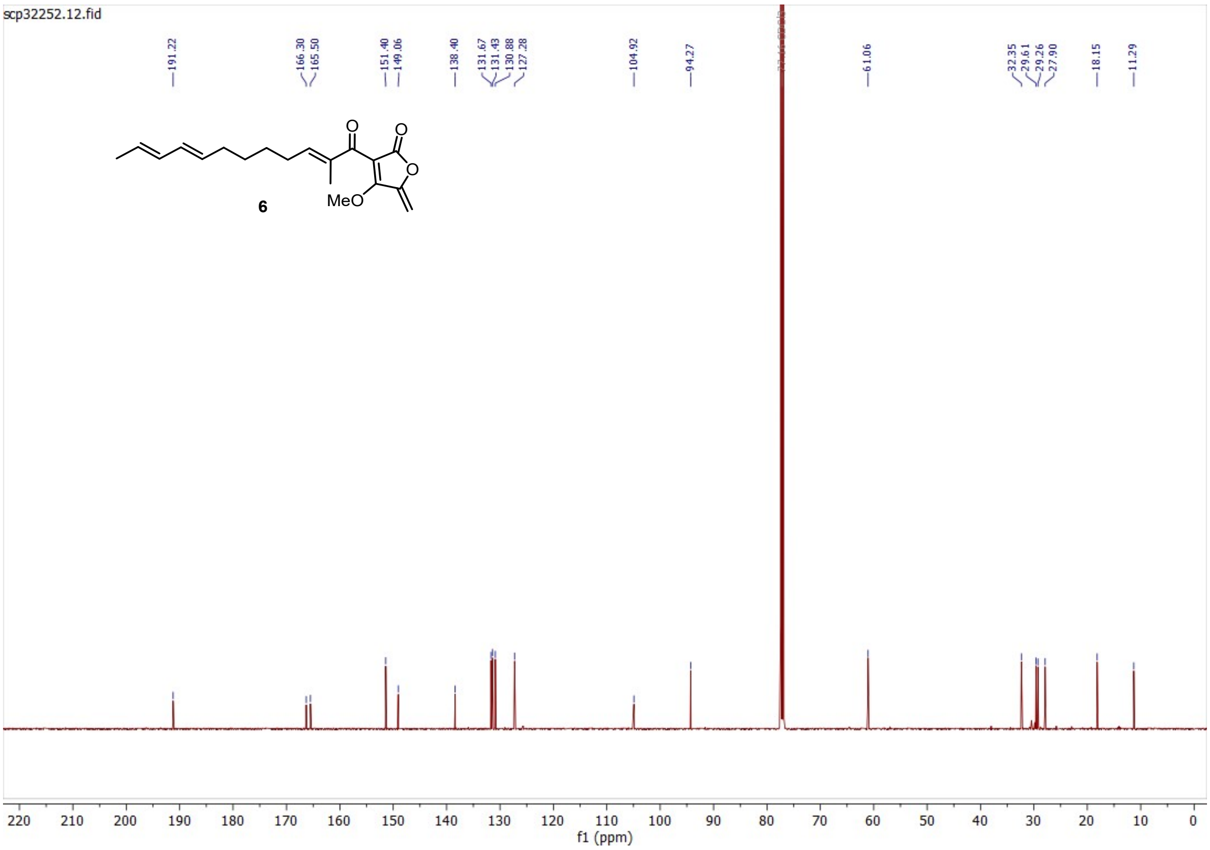
scp32252.12.fid
6
MeO
f1 (ppm)

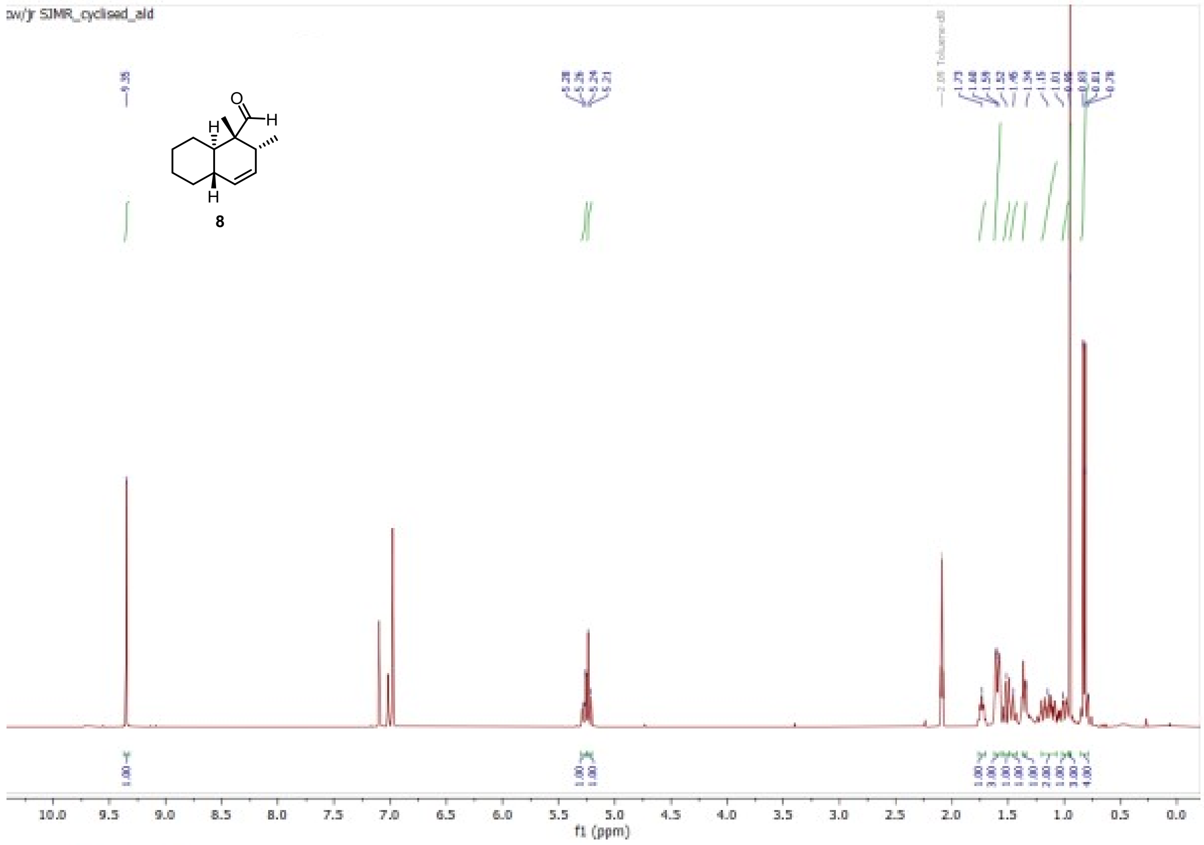
cw/jr SJMR_cyclised_ald
8
f1 (ppm)

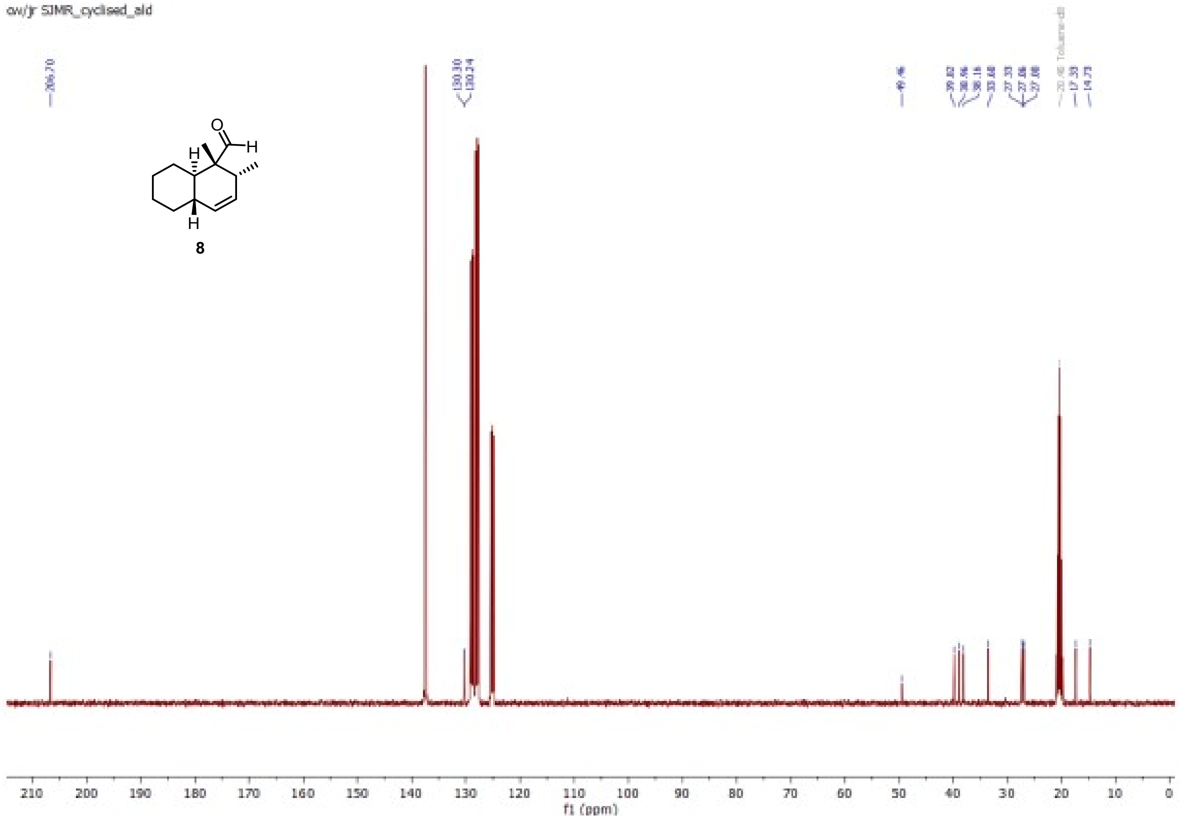
cw/jr SJMR_cyclised_ald
8
f1 (ppm)

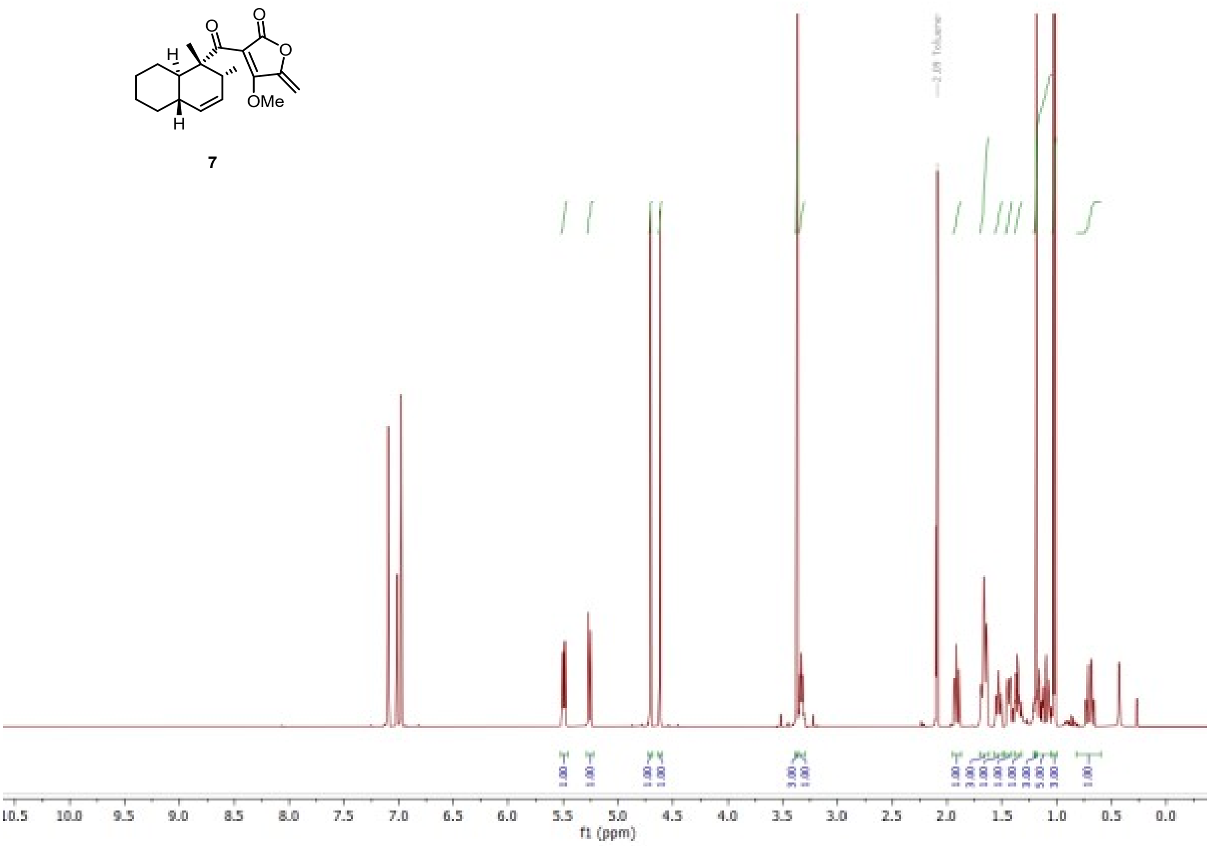
OMe
7
f1 (ppm)

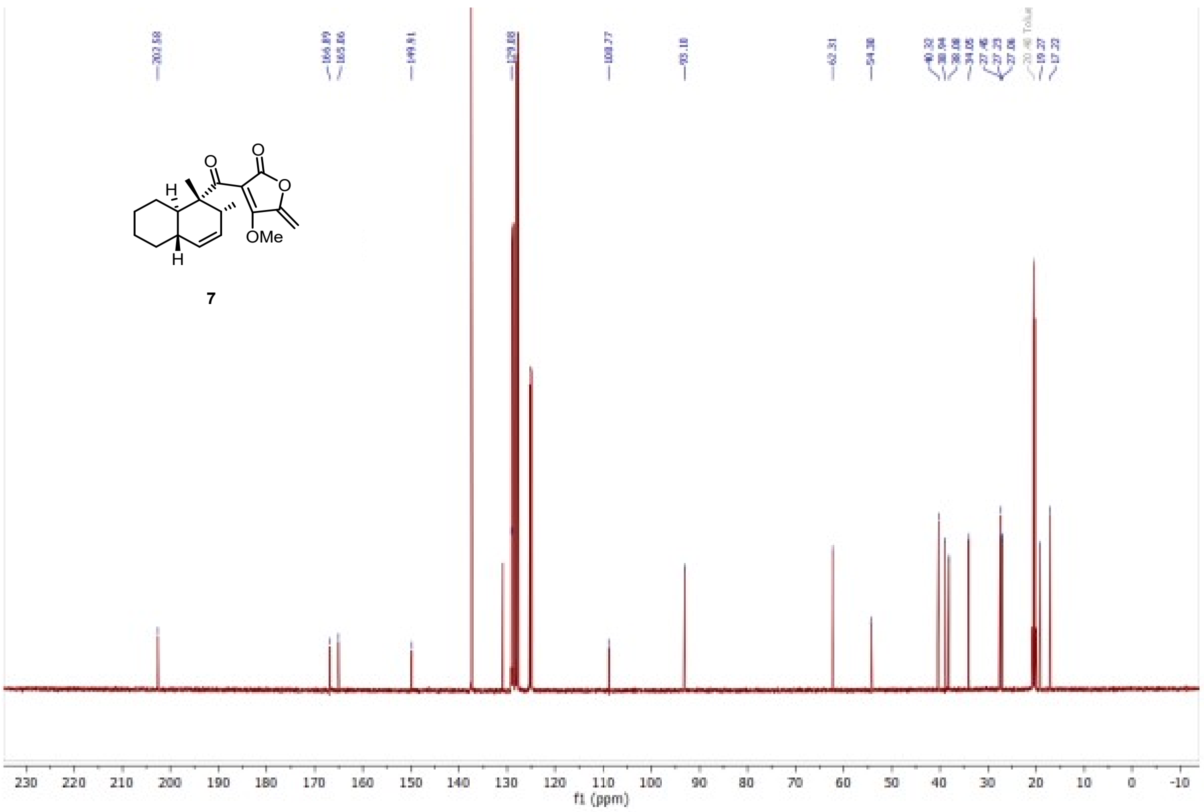
OMe
7
f1 (ppm)

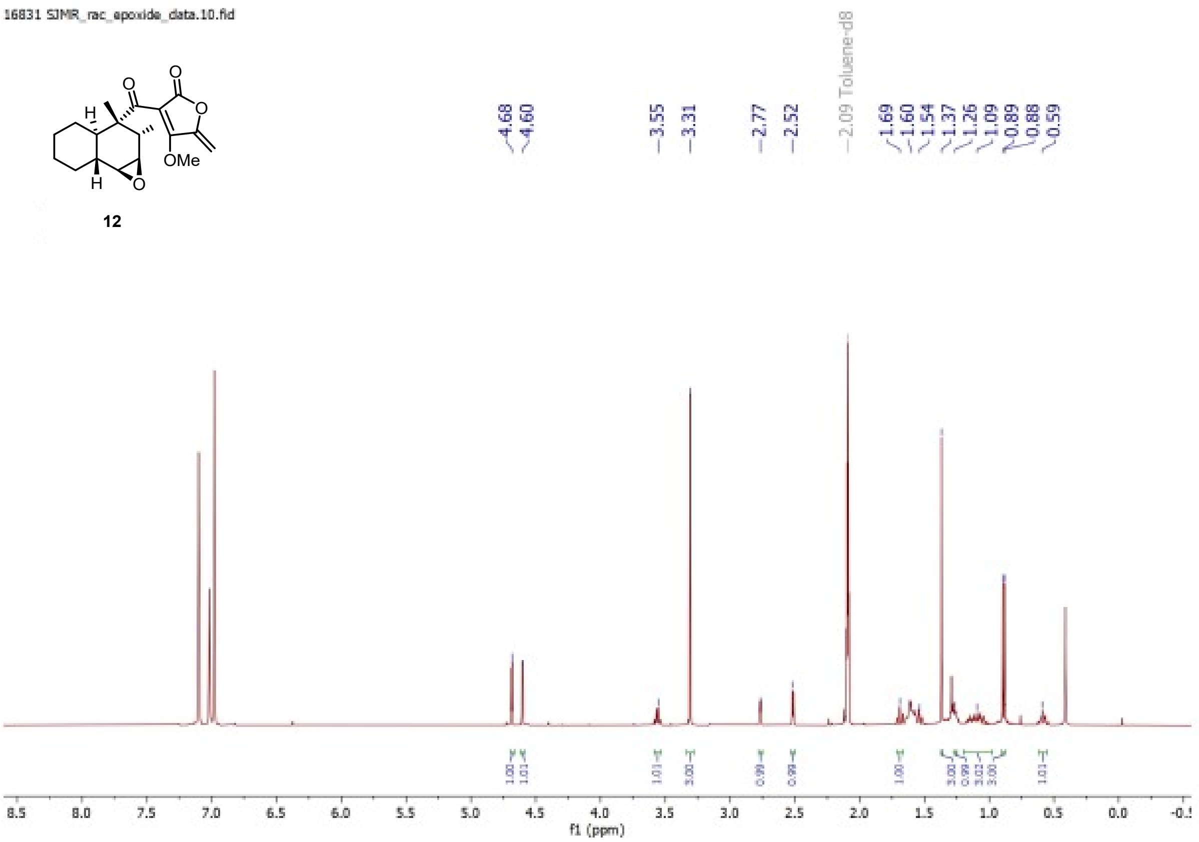
16831 SJMR_rac_epoxide_data.10.fid
O
O
O
H
OMe
H
O
12
4.68
4.60
3.55
3.31
2.77
2.52
2.09 Toluene-d8
1.69
1.60
1.54
1.37
1.26
1.09
0.89
0.88
0.59
1.00
1.01
1.01
3.00
0.99
0.99
1.00
3.00
0.99
3.01
3.00
1.01
8.5 8.0 7.5 7.0 6.5 6.0 5.5 5.0 4.5 4.0 3.5 3.0 2.5 2.0 1.5 1.0 0.5 0.0
f1 (ppm)

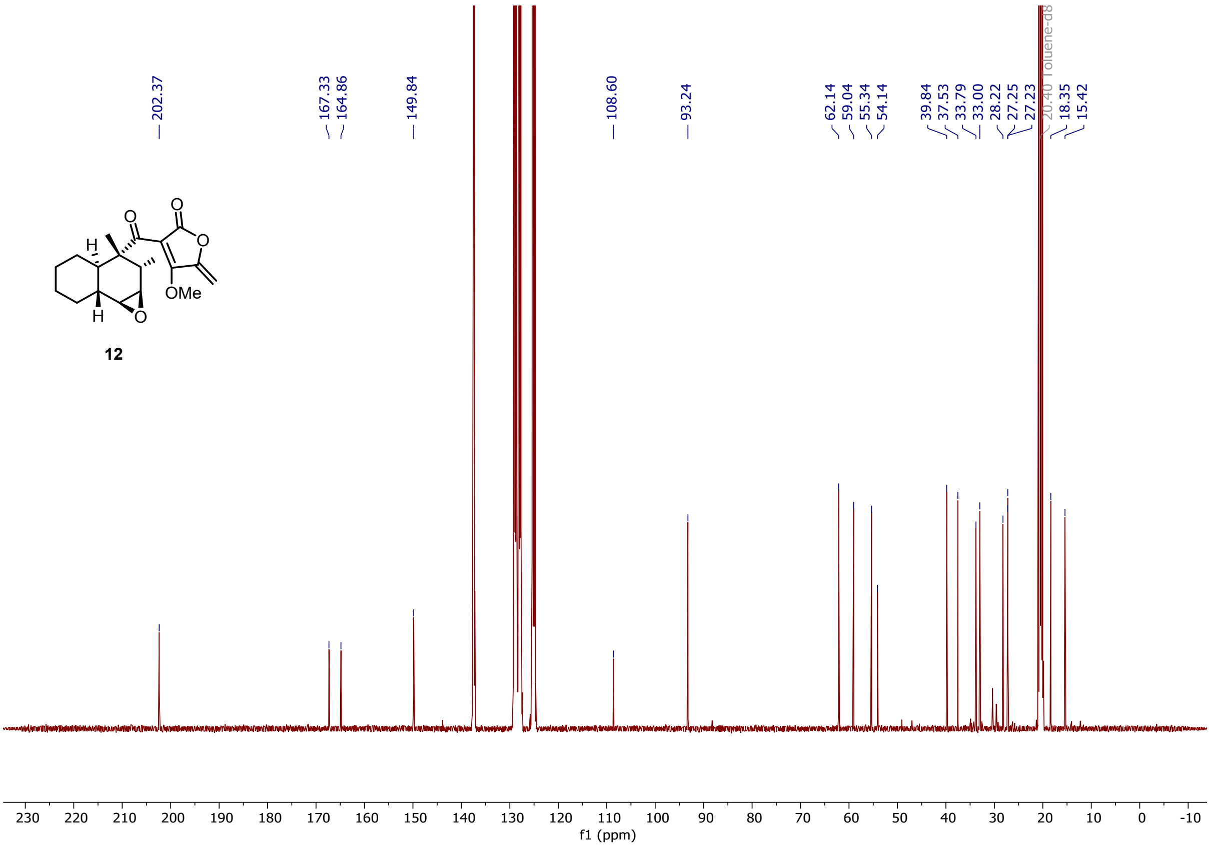
202.37
167.33
164.86
149.84
108.60
93.24
62.14
59.04
55.34
54.14
39.84
37.53
33.79
33.00
28.22
27.25
27.23
20.40 Toluene-d8
18.35
15.42
O
O
O
H
OMe
H
O
12
230 220 210 200 190 180 170 160 150 140 130 120 110 100 90 80 70 60 50 40 30 20 10 0 -10
f1 (ppm)

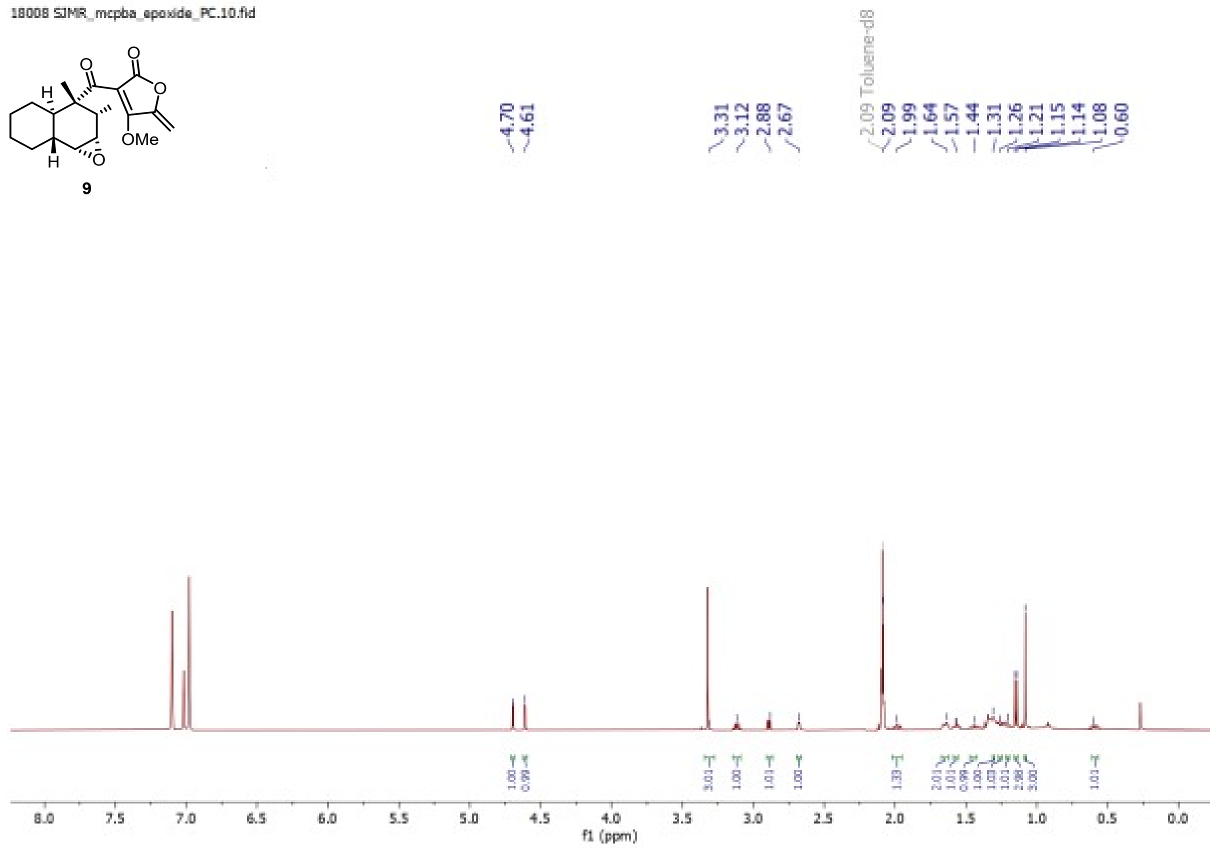
18008 SJMR_mcpba_epoxide_PC.10.fid
9
4.70
4.61
3.31
3.12
2.88
2.67
2.09 Toluene-d8
2.09
1.99
1.64
1.57
1.44
1.31
1.26
1.21
1.15
1.14
1.08
0.60
f1 (ppm)

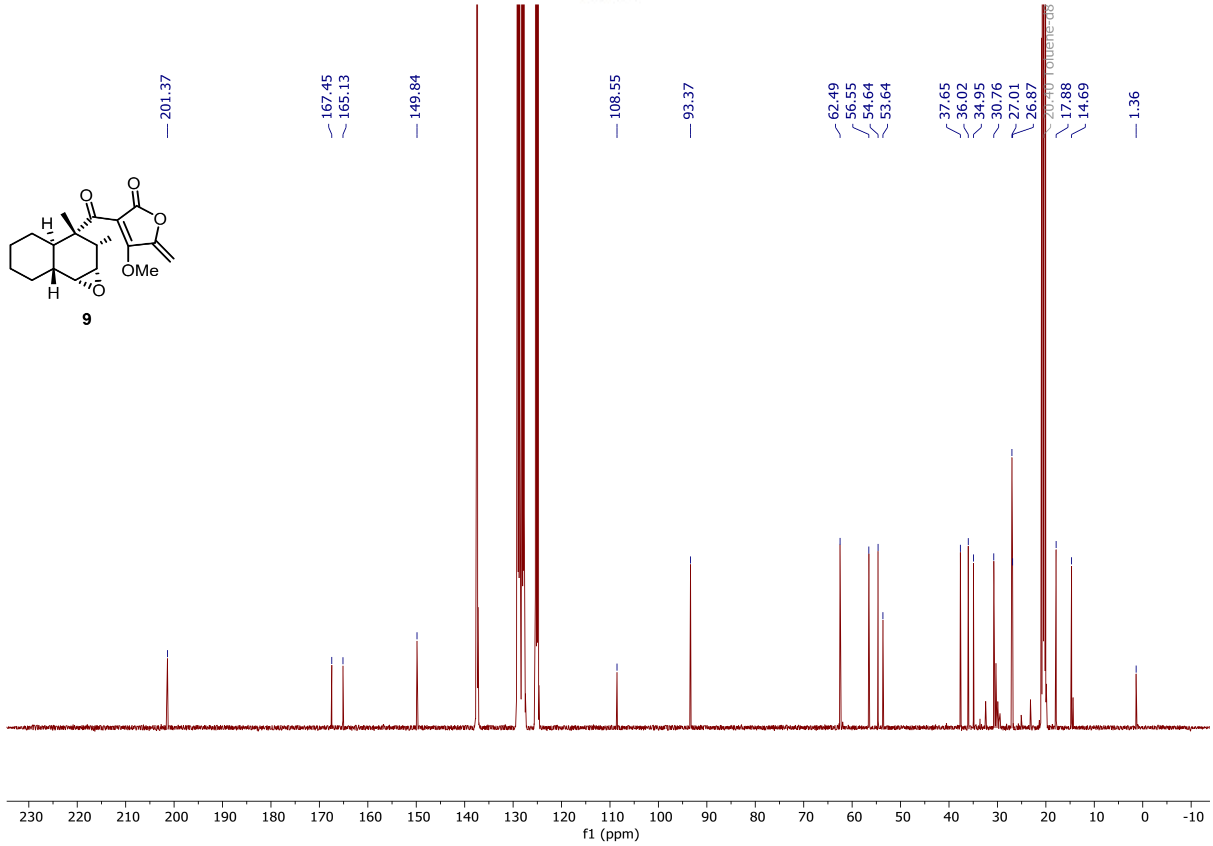
9
201.37
167.45
165.13
149.84
108.55
93.37
62.49
56.55
54.64
53.64
37.65
36.02
34.95
30.76
27.01
26.87
20.40 Toluene-d8
17.88
14.69
1.36
f1 (ppm)

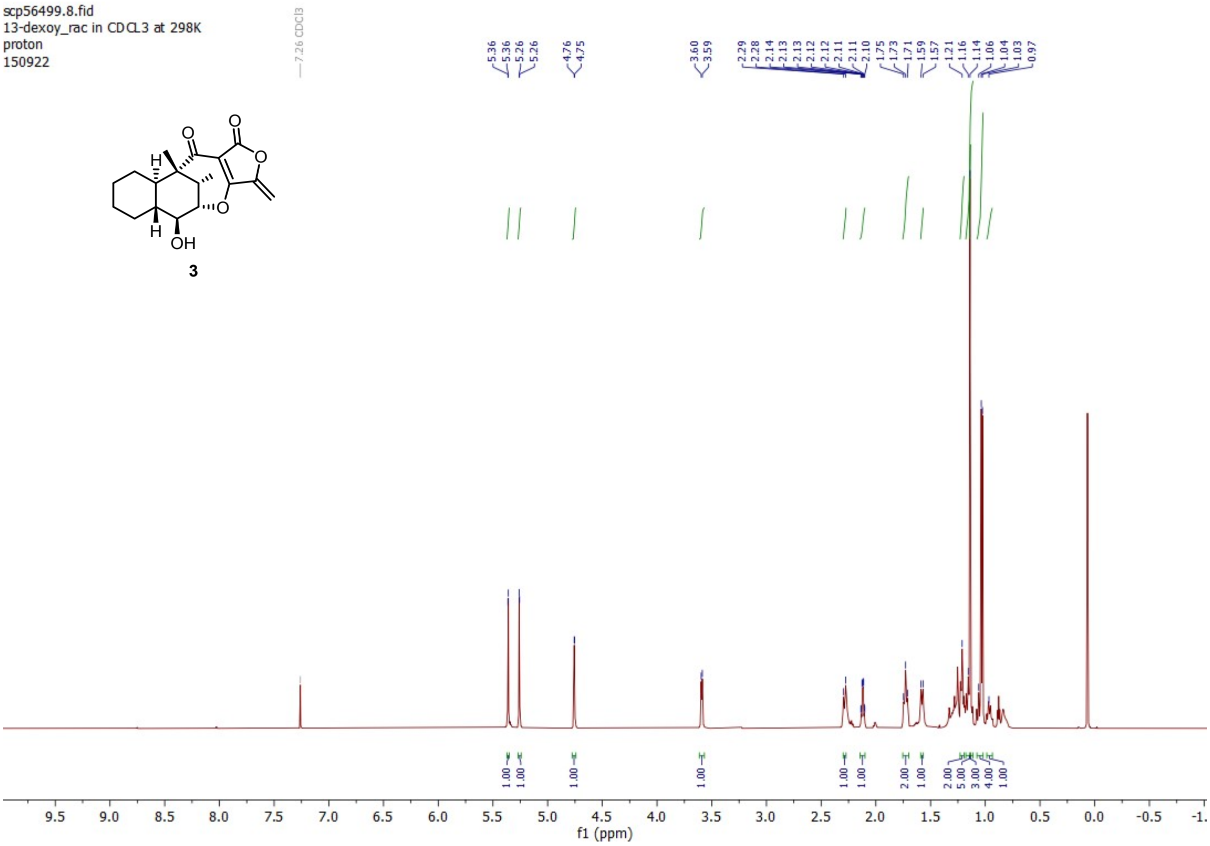
scp56499.8.fid
13-dexoy_rac in CDCL3 at 298K
proton
150922
3
f1 (ppm)

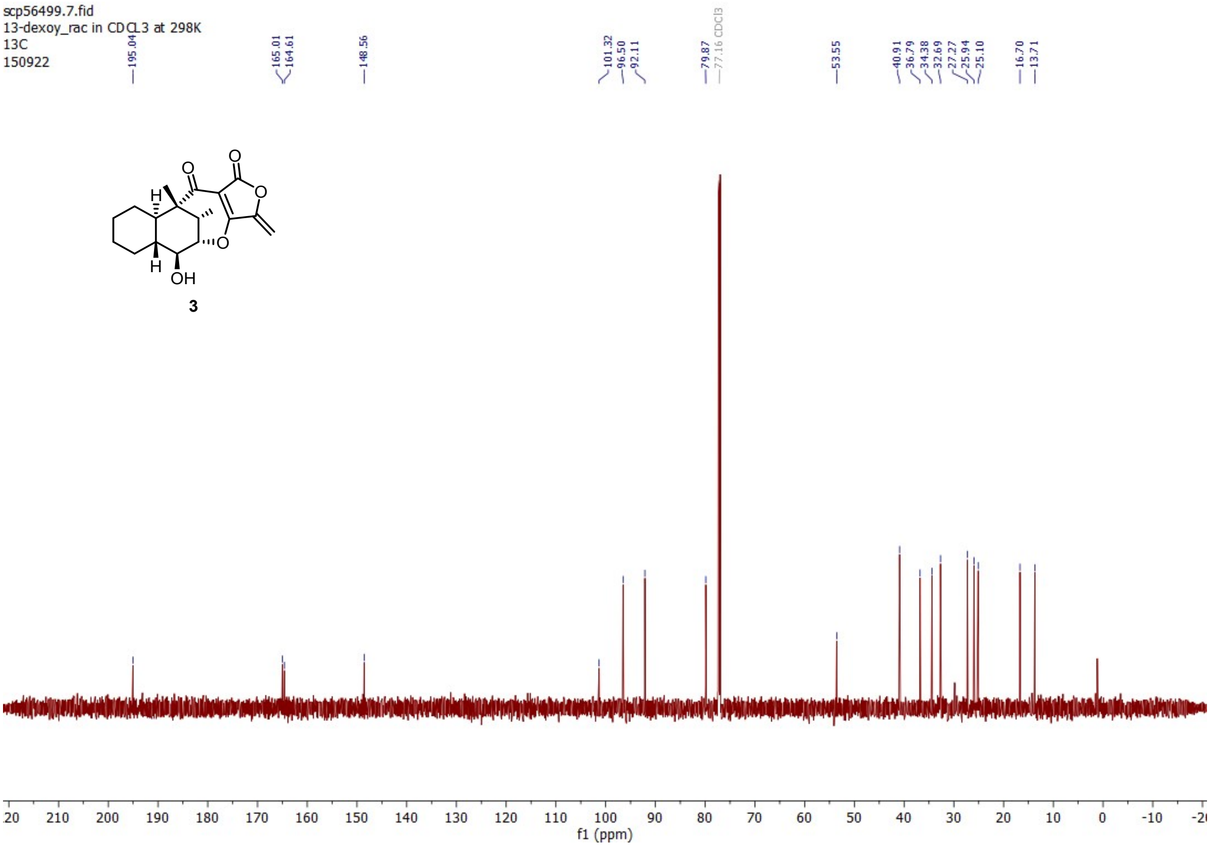
scp56499.7.fid
13-dexoy_rac in CDCL3 at 298K
13C
150922
3
f1 (ppm)

5. References

[1] N. S. Berrow, D. Alderton, S. Sainsbury, J. Nettleship, R. Assenberg, N. Rahman, D. I. Stuart, R. J. Owens, *Nucleic Acids Res.* **2007**, *35*, e45.

[2] X. Y. Jia, Z. H. Tian, L. Shao, X. D. Qu, Q. F. Zhao, J. Tang, G. L. Tang, W. Liu, *Chem. Biol.* **2006**, *13*, 575–585.

[3] W. Kabsch, *Acta Crystallogr. Sect. D Biol. Crystallogr.* **2010**, *66*, 125–132.

[4] G. Winter, C. M. C. Lobley, S. M. Prince, *Acta Crystallogr. Sect. D Biol. Crystallogr.* **2013**, *69*, 1260–1273.

[5] Q. Zheng, Y. Gong, Y. Guo, Z. Zhao, Z. Wu, Z. Zhou, D. Chen, L. Pan, W. Liu, *Cell Chem. Biol.* **2018**, *25*, 718-727.e3.

[6] A. Vagin, A. Teplyakov, *Acta Crystallogr. Sect. D Biol. Crystallogr.* **2010**, *66*, 22–25.

[7] L. Potterton, J. Agirre, C. Ballard, K. Cowtan, E. Dodson, P. R. Evans, H. T. Jenkins, R. Keegan, E. Krissinel, K. Stevenson, A. Lebedev, S. J. McNicholas, R. A. Nicholls, M. Noble, N. S. Pannu, C. Roth, G. Sheldrick, P. Skubak, J. Turkenburg, V. Uski, F. Von Delft, D. Waterman, K. Wilson, M. Winn, M. Wojdyr, *Acta Crystallogr. Sect. D Struct. Biol.* **2018**, *74*, 68–84.

[8] P. Emsley, B. Lohkamp, W. G. Scott, K. Cowtan, *Acta Crystallogr. Sect. D Biol. Crystallogr.* **2010**, *66*, 486–501.

[9] G. N. Murshudov, P. Skubák, A. A. Lebedev, N. S. Pannu, R. A. Steiner, R. A. Nicholls, M. D. Winn, F. Long, A. A. Vagin, *Acta Crystallogr. Sect. D Biol. Crystallogr.* **2011**, *67*, 355–367.

[10] M. J. Frisch, G. W. Trucks, H. B. Schlegel, G. E. Scuseria, M. A. Robb, J. R. Cheeseman, G. Scalmani, V. Barone, G. A. Petersson, H. Nakatsuji, X. Li, M. Caricato, A. V. Marenich, J. Bloino, B. G. Janesko, R. Gomperts, B. Mennucci, H. P. Hratchian, J. V. Ortiz, A. F. Izmaylov, J. L. Sonnenberg, D. Williams-Young, F. Ding, F. Lipparini, F. Egidi, J. Goings, B. Peng, A. Petrone, T. Henderson, D. Ranasinghe, V. G. Zakrzewski, J. Gao, N. Rega, G. Zheng, W. Liang, M. Hada, M. Ehara, K. Toyota, R. Fukuda, J. Hasegawa, M. Ishida, T. Nakajima, Y. Honda, O. Kitao, H. Nakai, T. Vreven, K. Throssell, J. A. Montgomery, Jr., J. E. Peralta, F. Ogliaro, M. J. Bearpark, J. J. Heyd, E. N. Brothers, K. N. Kudin, V. N. Staroverov, T. A. Keith, R. Kobayashi, J. Normand, K. Raghavachari, A. P. Rendell, J. C. Burant, S. S. Iyengar, J. Tomasi, M. Cossi, J. M. Millam, M. Klene, C. Adamo, R. Cammi, J. W. Ochterski, R. L. Martin, K. Morokuma, O. Farkas, J. B. Foresman, and D. J. Fox, Gaussian 16, Revision C.01, Gaussian, Inc., Wallingford CT, 2016.

[11] Y. Zhao, D. G. Truhlar, *Theor Chem Acc.* **2008**, *120*, 215–241.

[12] S. N. Pieniazek, F. R. Clemente, K. N. Houk, *Angew. Chem. Int. Ed.* **2008**, *47*, 7746–7749.

[13] M. Linder, T. Brinck, *Phys. Chem. Chem. Phys.* **2013**, *15*, 5108–5114.

[14] A. V. Marenich, C. J. Cramer, D. G. Truhlar, *J. Phys. Chem. B* **2009**, *113*, 6378–6396.

[15] Spartan'20 Wavefunction, Inc., Irvine, CA.

[16] J. W. Ochterski, Thermochemistry in Gaussian, **2000,** Available at: https://gaussian.com/wp-content/uploads/dl/thermo.pdf

[17] J. Eberhardt, D. Santos-Martins, A. F. Tillack, S. Forli, *J. Chem. Inf. Model.* **2021**, *61*, 3891–3898.

[18] K. Zinovjev, M. W. van der Kamp, *Bioinformatics* **2020**, *36*, 5104–5106.

[19] J. Wang, R. M. Wolf, J. W. Caldwell, P. A. Kollman, D. A. Case, *J. Comput. Chem.* **2004**, *25*, 1157–1174.

[20] J. Wang, W. Wang, P. A. Kollman, D. A. Case, *J. Mol. Graph. Model.* **2006**, *25*, 247–260.

[21] R. Shinohara, M. Morita, N. Ogawa, Y. Kobayashi, *Org. Lett.* **2019**, *21*, 3247–3251.

[22] G. A. Tolstikov, U. M. Dzhemilev, R. I. Khusnutdinov, *Chem. Nat. Compd.* **1978**, *14*, 101–102.

[23] J. He, J. E. Baldwin, V. Lee, *Synlett* **2018**, *29*, 1117–1121.

[24] M. Winterer, K. Kempf, R. Schobert, *J. Org. Chem.* **2016**, *81*, 7336–7341.

[25] S. W. Haynes, P. K. Sydor, A. E. Stanley, L. Song, G. L. Challis, *Chem. Commun.* **2008**, 1865–1867.

[26] K. Takeda, S. Yano, M. Sato, E. Yoshii, *J. Org. Chem.* **1987**, *52*, 4135–4137.

[27] L. J. Montgomery, G. L. Challis, *Synlett* **2008**, 2164–2168.

[28] T. S. Li, J. T. Li, H. Z. Li, *J. Chromatogr. A* **1995**, *715*, 372–375.

[29] T. Gverzdys, M. K. Hart, S. Pimentel-Elardo, G. Tranmer, J. R. Nodwell, *J. Antibiot.* **2015**, *68*, 698–702
